# Clinical and pathological associations of PTEN expression in ovarian cancer: a multicentre study from the Ovarian Tumour Tissue Analysis Consortium

**DOI:** 10.1038/s41416-020-0900-0

**Published:** 2020-06-18

**Authors:** Filipe Correia Martins, Dominique-Laurent Couturier, Anna Paterson, Anthony N. Karnezis, Christine Chow, Tayyebeh M. Nazeran, Adekunle Odunsi, Aleksandra Gentry-Maharaj, Aleksandra Vrvilo, Alexander Hein, Aline Talhouk, Ana Osorio, Andreas D. Hartkopf, Angela Brooks-Wilson, Anna DeFazio, Anna Fischer, Arndt Hartmann, Brenda Y. Hernandez, Bryan M. McCauley, Chloe Karpinskyj, Christiani B. de Sousa, Claus Høgdall, Daniel G. Tiezzi, Esther Herpel, Florin Andrei Taran, Francesmary Modugno, Gary Keeney, Gregg Nelson, Helen Steed, Honglin Song, Hugh Luk, Javier Benitez, Jennifer Alsop, Jennifer M. Koziak, Jenny Lester, Joseph H. Rothstein, Jurandyr M. de Andrade, Lene Lundvall, Luis Paz-Ares, Luis Robles-Díaz, Lynne R. Wilkens, Maria J. Garcia, Maria P. Intermaggio, Marie-Lyne Alcaraz, Mary A. Brett, Matthias W. Beckmann, Mercedes Jimenez-Linan, Michael Anglesio, Michael E. Carney, Michael Schneider, Nadia Traficante, Nadja Pejovic, Naveena Singh, Nhu Le, Peter Sinn, Prafull Ghatage, Ramona Erber, Robert Edwards, Robert Vierkant, Roberta B. Ness, Samuel Leung, Sandra Orsulic, Sara Y. Brucker, Scott H. Kaufmann, Sian Fereday, Simon Gayther, Stacey J. Winham, Stefan Kommoss, Tanja Pejovic, Teri A. Longacre, Valerie McGuire, Valerie Rhenius, Weiva Sieh, Yurii B. Shvetsov, Alice S. Whittemore, Annette Staebler, Beth Y. Karlan, Cristina Rodriguez-Antona, David D. Bowtell, Ellen L. Goode, Estrid Høgdall, Francisco J. Candido dos Reis, Jacek Gronwald, Jenny Chang-Claude, Kirsten B. Moysich, Linda E. Kelemen, Linda S. Cook, Marc T. Goodman, Peter A. Fasching, Robin Crawford, Suha Deen, Usha Menon, David G. Huntsman, Martin Köbel, Susan J. Ramus, Paul D. P. Pharoah, James D. Brenton

**Affiliations:** 1 Department of Obstetrics and Gynaecology, University of Cambridge, Cambridge, England; 2 Experimental Medicine Initiative, University of Cambridge, Cambridge, England; 3 Cancer Research UK Cambridge Institute, University of Cambridge, Li Ka Shing Centre, Robinson Way, Cambridge, England; 4 Department of Histopathology, Addenbrookes Hospital, Cambridge, England; 5 Department of Pathology and Laboratory Medicine, University of California Davis Medical Center, Sacramento, CA USA; 6 OVCARE, Vancouver Coastal Health Research Centre, Vancouver General Hospital and University of British Columbia, Vancouver, BC Canada; 7 Department of Molecular Oncology and Department of Pathology and Laboratory Medicine, BC Cancer Research Centre, BC Cancer, Vancouver, BC Canada; 8 Department of Cancer Prevention and Control, Roswell Park Cancer Institute, Buffalo, NY USA; 9 MRC CTU, Institute of Clinical Trials and Methodology, University College London, London, England; 10 Department of Ob/Gyn, Oregon Health & Science University, Portland, OR USA; 11 Department of Gynecology and Obstetrics, University Hospital Erlangen, Comprehensive Cancer Center Erlangen-EMN, Friedrich-Alexander University Erlangen-Nuremberg, Erlangen, Germany; 12 Department of Obstetrics and Gynecology, University of British Columbia, Vancouver, BC Canada; 13 Department of Molecular Oncology, BC Cancer Research Centre, BC Cancer, Vancouver, BC Canada; 14 Human Genetics Group, Spanish National Cancer Research Centre (CNIO), Madrid, Spain; 15 Centre for Biomedical Network Research on Rare Diseases (CIBERER), Instituto de Salud Carlos III, Madrid, Spain; 16 Department of Women’s Health, Tübingen University Hospital, Tübingen, Germany; 17 Canada’s Michael Smith Genome Sciences Centre, British Columbia Cancer Agency, Vancouver, BC Canada; 18 Centre for Cancer Research, The Westmead Institute for Medical Research, The University of Sydney, Sydney, NSW Australia; 19 Department of Gynaecological Oncology, Westmead Hospital, Sydney, NSW Australia; 20 Institute of Pathology, Tübingen University Hospital, Tübingen, Germany; 21 Institute of Pathology, University Hospital Erlangen, Comprehensive Cancer Center Erlangen-EMN, Friedrich-Alexander University Erlangen-Nuremberg, Erlangen, Germany; 22 Cancer Center, University of Hawaii, Honolulu, HI USA; 23 Department of Health Sciences Research, Mayo Clinic, Rochester, MN USA; 24 Department of Gynecology and Obstetrics, Ribeirão Preto Medical School, University of São Paulo, Ribeirão Preto, Brazil; 25 Department of Gynaecology, Rigshospitalet, University Hospital Copenhagen, Blegdamsvej 9, 2100 København Denmark; 26 NCT Tissue Bank, National Center for Tumour Diseases, Heidelberg, Germany; 27 Institute of Pathology, University Hospital Heidelberg, Heidelberg, Germany; 28 Department of Obstetrics, Gynecology and Reproductive Sciences, University of Pittsburgh School of Medicine, Pittsburgh, PA USA; 29 Department of Oncology, Division of Gynecologic Oncology, Cumming School of Medicine, University of Calgary, Calgary, AB Canada; 30 Department of Obstetrics and Gynecology, Division of Gynecologic Oncology, Royal Alexandra Hospital, Edmonton, AB Canada; 31 Department of Oncology, Strangeways Research Laboratory, University of Cambridge, Cambridge, England; 32 Epidemiology Program, University of Hawaii Cancer Center, Honolulu, HI USA; 33 Alberta Health Services–Cancer Care, Calgary, AB Canada; 34 Women’s Cancer Program, Samuel Oschin Comprehensive Cancer Institute, Cedars-Sinai Medical Center, Los Angeles, CA USA; 35 Department of Population Health Science and Policy and Department of Genetics and Genomic Sciences, Icahn School of Medicine at Mount Sinai, New York, NY USA; 36 Spanish National Cancer Research Center, CNIO Lung Cancer Clinical Research Unit, New York, NY USA; 37 Familial Cancer Unit and Medical Oncology Department, Hospital Universitario 12 de Octubre, Madrid, Spain; 38 School of Women’s and Children’s Health, University of New South Wales, Sydney, NSW Australia; 39 Department of Pathology and Laboratory Medicine, University of Calgary, Calgary, AB Canada; 40 John A. Burns School of Medicine, Department of Obstetrics and Gynecology, University of Hawaii, Honolulu, HI USA; 41 Sir Peter MacCallum Department of Oncology, University of Melbourne, Parkville, VIC Australia; 42 Peter MacCallum Cancer Centre, East Melbourne, VIC Australia; 43 School of Medicine, St. Louis University, St. Louis, MO 63103 USA; 44 Department of Cellular Pathology, Barts Health National Health Service Trust, London, England; 45 Cancer Control Research, British Columbia Cancer Agency, Vancouver, BC Canada; 46 University of Texas School of Public Health, Houston, TX USA; 47 Cedars-Sinai Center for Bioinformatics and Functional Genomics, Los Angeles, CA USA; 48 Department of Pathology, Stanford University School of Medicine, Stanford, CA USA; 49 Department of Health Research and Policy, Stanford University School of Medicine, Stanford, CA USA; 50 Department of Pathology, University of Melbourne, Melbourne, VIC Australia; 51 Department of Health Research and Policy and Department of Biomedical Data Science, Stanford University School of Medicine, Stanford, CA USA; 52 Hereditary Endocrine Cancer Group, Spanish National Cancer Research Center (CNIO), Madrid, Spain; 53 Centre for Biomedical Network Research on Rare Diseases (CIBERER), Instituto de Salud Carlos III Madrid, Madrid, Spain; 54 Sir Peter MacCallum Department of Oncology, University of Melbourne, Parkville, Parkville, VIC Australia; 55 The Garvan Institute, Sydney, NSW Australia; 56 Virus, Lifestyle and Genes, Danish Cancer Society Research Center, Copenhagen, Denmark; 57 Molecular Unit, Department of Pathology, Herlev Hospital, University of Copenhagen, Copenhagen, Denmark; 58 Cancer Genomics Program, Research Department, Molecular Unit, Department of Pathology, Herlev Hospital, University of Copenhagen, Copenhagen, Denmark; 59 International Hereditary Cancer Center, Department of Genetics and Pathology, Pomeranian Medical University, Szczecin, Poland; 60 Division of Cancer Epidemiology, German Cancer Research Center (DKFZ), Heidelberg, Germany; 61 University Cancer Center Hamburg, University Medical Center Hamburg-Eppendorf, Hamburg, Germany; 62 Department of Public Health Sciences, Medical University of South Carolina and Hollings Cancer Center, Charleston, SC USA; 63 Division of Epidemiology, Biostatistics and Preventative Medicine, University of New Mexico, Albuquerque, NM USA; 64 Samuel Oschin Comprehensive Cancer Institute, Cedars-Sinai Medical Center, Los Angeles, CA USA; 65 David Geffen School of Medicine, Department of Medicine Division of Hematology and Oncology, University of California at Los Angeles, Los Angeles, CA 90095 USA; 66 Division of Oncology, Addenbrookes Hospital, Cambridge, England; 67 Department of Histopathology, Queen’s Medical Centre, Nottingham University Hospitals NHS Trust, Nottingham, England; 68 Department of Public Health and Primary Care, Strangeways Research Laboratory, University of Cambridge, Worts Causeway, Cambridge, England; 69 Cancer Research UK Cambridge Centre, University of Cambridge, Cambridge, England; 70 Department of Oncology, Hutchison/MRC Research Centre, University of Cambridge, Cambridge, England

**Keywords:** Molecular medicine, Ovarian cancer

## Abstract

**Background:**

PTEN loss is a putative driver in histotypes of ovarian cancer (high-grade serous (HGSOC), endometrioid (ENOC), clear cell (CCOC), mucinous (MOC), low-grade serous (LGSOC)). We aimed to characterise PTEN expression as a biomarker in epithelial ovarian cancer in a large population-based study.

**Methods:**

Tumours from 5400 patients from a multicentre observational, prospective cohort study of the Ovarian Tumour Tissue Analysis Consortium were used to evaluate associations between immunohistochemical PTEN patterns and overall survival time, age, stage, grade, residual tumour, CD8+ tumour-infiltrating lymphocytes (TIL) counts, expression of oestrogen receptor (ER), progesterone receptor (PR) and androgen receptor (AR) by means of Cox proportional hazard models and generalised Cochran–Mantel–Haenszel tests.

**Results:**

Downregulation of cytoplasmic PTEN expression was most frequent in ENOC (most frequently in younger patients; *p* value = 0.0001) and CCOC and was associated with longer overall survival in HGSOC (hazard ratio: 0.78, 95% CI: 0.65–0.94, *p* value = 0.022). PTEN expression was associated with ER, PR and AR expression (*p* values: 0.0008, 0.062 and 0.0002, respectively) in HGSOC and with lower CD8 counts in CCOC (*p* value < 0.0001). Heterogeneous expression of PTEN was more prevalent in advanced HGSOC (*p* value = 0.019) and associated with higher CD8 counts (*p* value = 0.0016).

**Conclusions:**

PTEN loss is a frequent driver in ovarian carcinoma associating distinctly with expression of hormonal receptors and CD8+ TIL counts in HGSOC and CCOC histotypes.

## Background

Ovarian cancer is the fifth leading cause of female cancer mortality and advances in the last decades have not translated into increased survival.^1^ High-grade serous ovarian cancer (HGSOC) is the most common histotype of ovarian cancer and is characterised by extreme genomic instability and chromosomal rearrangements. HGSOC has ubiquitous *TP53* mutation, common mutations of *BRCA1* and *BRCA2* and amplification of *CCNE1*, *MYC* and *PIK3CA* (reviewed in ref. ^2^). Other common ovarian cancer histotypes are endometrioid (ENOC), clear cell (CCOC), mucinous (MOC) and low-grade serous (LGSOC) which have specific drivers and different cells of origin.^1^

Phosphatase and tensin homologue (PTEN) is a potent tumour suppressor classically known for its role in the inhibition of the phosphoinositide-3 kinase (PI3K) pathway. PTEN regulates cell proliferation, migration, survival, genomic stability and metabolism by phosphatase-dependent and phosphatase-independent activities.^3^ Loss of PTEN was associated with immunoresistance and worse response to programmed cell death protein 1 (PD-1) inhibitors in preclinical murine models of melanoma, by decreasing T cell trafficking into the tumours and T cell-mediated cell death in the tumour. Treatment with selective PI3Kbeta inhibitor improved the efficacy of both anti-PD-1 and anti-cytotoxic T-lymphocyte-associated protein 4 (anti-CTLA-4) antibodies in mouse models.^3,4^ Even subtle changes of PTEN expression may influence tumour initiation and progression as it is strongly regulated by critical molecular networks in which activation of hormonal receptors seems to have an important role.^5^

In ovarian cancer, mutation of *PTEN* has been a reported driver in endometrioid and clear cell subtypes.^6–10^ Homozygous loss of *PTEN* is found in 6% of HGSOC^11^ and loss-of-function mutations of *Pten* allow for accelerated tumour growth in mouse and in vitro models of HGSOC and ENOC.^12–14^ We previously demonstrated that *PTEN* loss is prevalent in HGSOC using bioinformatics and image analysis methods that corrected for cellularity in gene expression signatures from The Cancer Genome Atlas.^15^ *PTEN* and *AR* gene expression were significantly correlated and had positive survival effects. However, this and previous studies are limited by modest sample sizes and potential effects of different assay methods. Large-scale analysis of PTEN protein expression has not been carried out across the ovarian histotypes.

Therefore, we hypothesised that accurate estimates of PTEN loss across a large population would reveal histotype-specific associations with clinical and pathological factors using clinically relevant immunohistochemical (IHC) assays for PTEN, CD8 and the hormone receptors oestrogen receptor (ER), progesterone receptor (PR) and androgen receptor (AR).^16,17^ The prevalence of PTEN loss and the above associations will inform the likelihood of loss of PTEN being a driver.

## Methods

### Study design and participants

A total of 5400 women with ovarian cancer from 20 participating sites in the Ovarian Tumour Tissue Analysis (OTTA) Consortium with an average length of follow-up of 5.5 years were included in this study. We requested and received institutional review board/ethics board approval from the institutions participating in the OTTA Consortium. Patients were not provided compensation for their participation in the study at any site. Average length of follow-up per histotype were approximately 4.4 years for HGSOC, 7.6 for ENOC, 7.2 for CCOC, 6.6 for MOC and 5.8 for LGSOC (Table 1; Supplementary Table S1). One-third of the patients had delayed entry into the study, and for these cases, the mean enrolment time was 1.4 years post-diagnosis.

**Table 1:** Summary of the demographics of the study cohort stratified by cancer histotype.

	Cancer type					Total
	HGSOC	ENOC	CCOC	MOC	LGSOC	
*Age at diagnosis*						
Mean	60.4	55.4	56.6	55.2	53.9	58.5
SD	11.2	12.4	11.7	15.2	13.1	12.1
*Years followed*						
Mean	4.4	7.6	7.2	6.6	5.8	5.5
SD	3.6	4.8	5.6	5.3	4.3	4.5
*Delayed entry*						
Mean	0.4	0.6	0.5	0.6	0.6	0.5
Yes (%)	31.1	33.9	31.8	36.3	30.7	32
Mean if yes	1.2	1.9	1.7	1.8	1.9	1.4
SD if yes	1.7	2.3	2.5	2.2	1.9	2
*Number of cases (% per cancer subtype)*						
Total	3244 (100)	840 (100)	693 (100)	405 (100)	218 (100)	5400 (100)
Status at follow-up						
Data available	3185 (100)	810 (100)	673 (100)	396 (100)	213 (100)	5277 (100)
Alive	908 (29)	550 (68)	369 (55)	238 (60)	112 (53)	2177 (41)
Dead (disease)	1619 (51)	107 (13)	140 (21)	69 (17)	71 (33)	2006 (38)
Dead (treatment)	17 (1)	4 (0)	2 (0)	0 (0)	2 (1)	25 (0)
Dead (other)	113 (4)	40 (5)	28 (4)	22 (6)	3 (1)	206 (4)
Dead (unknown)	528 (17)	109 (13)	134 (20)	67 (17)	25 (12)	863 (16)
FIGO stage						
Data available	2882 (100)	667 (100)	591 (100)	324 (100)	179 (100)	4643 (100)
Stage I	236 (8)	342 (51)	295 (50)	217 (67)	45 (25)	1135 (24)
Stage II	284 (10)	189 (28)	154 (26)	36 (11)	18 (10)	681 (15)
Stage III	1994 (69)	122 (18)	130 (22)	60 (19)	104 (58)	2410 (52)
Stage IV	368 (13)	14 (2)	12 (2)	11 (3)	12 (7)	417 (9)
Differentiation						
Data available	2952 (100)	792 (100)	452 (100)	381 (100)	209 (100)	4786 (100)
Well	1 (0)	328 (41)	16 (4)	151 (40)	176 (84)	672 (14)
Moderate	376 (13)	251 (32)	57 (13)	173 (45)	3 (1)	860 (18)
Poor/none	2575 (87)	213 (27)	379 (84)	57 (15)	30 (14)	3254 (68)
Residual tumour						
Data available	2118 (100)	462 (100)	444 (100)	237 (100)	133 (100)	3394 (100)
Yes	1176 (56)	57 (12)	91 (20)	57 (24)	62 (47)	1443 (43)
No	942 (44)	405 (88)	353 (80)	180 (76)	71 (53)	1951 (57)
Cytoplasmic PTEN						
Data available	2915 (100)	775 (100)	644 (100)	360 (100)	185 (100)	4879 (100)
Negative	550 (19)	273 (35)	208 (32)	69 (19)	21 (11)	1121 (23)
Weak	1455 (50)	312 (40)	312 (48)	147 (41)	90 (49)	2316 (47)
Positive	733 (25)	157 (20)	107 (17)	124 (34)	64 (35)	1185 (24)
Heterogeneous	177 (6)	33 (4)	17 (3)	20 (6)	10 (5)	257 (5)
Nuclear PTEN						
Data available	2910 (100)	774 (100)	643 (100)	359 (100)	185 (100)	4871 (100)
0%	1211 (42)	483 (62)	288 (45)	171 (48)	68 (37)	2221 (46)
[0, 10]%	793 (27)	141 (18)	153 (24)	77 (21)	52 (28)	1216 (25)
[10, 50]%	679 (23)	114 (15)	146 (23)	72 (20)	53 (29)	1064 (22)
[50, 100]%	227 (8)	36 (5)	56 (9)	39 (11)	12 (6)	370 (8)
CD8 count						
Data available	2893 (100)	758 (100)	627 (100)	325 (100)	154 (100)	4757 (100)
0 TIL	479 (17)	193 (25)	295 (47)	157 (48)	40 (26)	1164 (24)
1–2 TIL	481 (17)	136 (18)	135 (22)	73 (22)	40 (26)	865 (18)
3–19 TIL	1278 (44)	301 (40)	119 (19)	82 (25)	63 (41)	1843 (39)
20+ TIL	655 (23)	128 (17)	78 (12)	13 (4)	11 (7)	885 (19)
AR expression						
Data available	2603 (100)	662 (100)	564 (100)	317 (100)	175 (100)	4321 (100)
Negative	1669 (64)	459 (69)	532 (94)	305 (96)	109 (62)	3074 (71)
Positive	934 (36)	203 (31)	32 (6)	12 (4)	66 (38)	1247 (29)
PR expression						
Data available	1674 (100)	590 (100)	504 (100)	267 (100)	86 (100)	3121 (100)
Negative	1068 (64)	167 (28)	469 (93)	235 (88)	34 (40)	1973 (63)
1–50% positive	457 (27)	111 (19)	25 (5)	18 (7)	26 (30)	637 (20)
>50% positive	149 (9)	312 (53)	10 (2)	14 (5)	26 (30)	511 (16)
ER expression						
Data available	1258 (100)	363 (100)	336 (100)	161 (100)	66 (100)	2184 (100)
Negative	289 (23)	91 (25)	283 (84)	133 (83)	9 (14)	805 (37)
1–50% positive	277 (22)	66 (18)	21 (6)	6 (4)	13 (20)	383 (18)
>50% positive	692 (55)	206 (57)	32 (10)	22 (14)	44 (67)	996 (46)

The same information in person-year units is available in Supplementary Table 1.

*TIL* tumour-infiltrating lymphocytes.

Demographic, clinical and pathological variables, including age at diagnosis, tumour stage and grade, presence or absence of post-operative residual tumour and scores for expression of PTEN, CD8, PR, AR and ER expression were used for the association analysis. Table 1 shows the absolute and relative number of histotype-specific participants with the variables mentioned above. Samples were obtained from pelvic disease and all the histological diagnosis were reviewed by experienced pathologists. Supplementary Table 1 provides information on variable-specific times of follow-up in person-years. Supplementary Fig. 1 summarises the analysis of missing data. Site dependency in missing data pattern was mostly due to different entry times into OTTA for different participant sites. Age at diagnosis was rarely missing (0.5%). The average age at diagnosis was slightly >60 years for HGSOC and around 55 years for other histotypes. Status at last follow-up, time of last follow-up and time of enrolment were mostly present (missed in 2.25, 1.5 and 0.65% of the cases, respectively). In all, 41% of the patients were still alive at the time of last follow-up. Causes of death were disease or treatment in 65.5% of the patients and unknown in 27.8% of the patients.

### IHC analysis and scoring

IHC for PTEN was carried out in Cambridge (UK) using tissue microarrays (TMAs) obtained from formalin-fixed, paraffin-embedded tissues and primary antibody for PTEN (Cell Signaling, Danvers, MA, USA; PTEN—Clone 138G6) as previously described.^15,18^ The PTEN expression assay was previously validated using external positive and negative controls including mouse tissues from a conditional *PTEN* knockout model.^18^ PTEN scoring used normal stroma as an internal control. Heat-induced antigen retrieval was carried out in 10 mmol l^−1^ citric acid (pH 6.0) in a pressure cooker at 120 °C for 10 min. Sections were incubated overnight with PTEN antibody diluted 1:100 in 5% goat serum, followed by a 1-h incubation with anti-rabbit biotinylated secondary antibody and peroxidase-conjugated avidin–biotin complexes (Elite ABC; Vector Laboratories, Burlingame, CA, USA). Formed immunocomplexes were visualised using diaminobenzidine (DAB; DAKO, Glostrup, Denmark, EU), and slides were counterstained with haematoxylin. Sections were rinsed in phosphate-buffered saline between each step. IHC staining for AR was performed in Vancouver using the Ventana Discovery Ultra machine (Ventana Medical Systems Inc., Tuscon, AZ, USA). Sections underwent 36 min of Cell Conditioning 1 (Ventana Medical Systems) before incubation with AR antibody (Santa Cruz sc-815) titrated at 1:50 for 60 min at 37 °C. Antibody expression was detected using the DAB Map Detection Kit (Ventana Medical Systems) with the Universal Secondary Antibody (Ventana Medical Systems). Updated data for CD8, ER and PR staining was obtained from the OTTA Consortium.^16,17^

IHC samples were stored at room temperature (RT) for at least 48 h before image analysis. For IHC, an Ariol scanning system (Leica, Wetzlar, Germany) was used to obtain the digital images. Scoring was performed according to intensity (using stromal cells as internal positive controls) and the percentage of stained cells. Reproducibility was scored by two independent observers (A.P., F.C.M.) from a randomly assigned data set of 678 cases taken from all TMAs. Cytoplasmic and nuclear staining in the tumour cells was assessed separately. Cytoplasmic staining was scored as negative (no staining in any tumour cell with internal stromal control present), weak positive (all tumour cells with weaker staining compared to stromal cells), positive (all tumour and stromal cells equally stained) or heterogeneous (combination of positive and negative/weak staining) staining. Nuclear staining was scored based on the percentage of cells with positive nuclei for PTEN (0%; 0–10%; 10–50%; >50%).

For AR IHC, positive was defined as nuclear expression in >1% of tumour cells.

### Statistical analysis

We used the generalised Cochran–Mantel–Haenszel (gCMH) tests to perform nominal and/or ordinal associations between cytoplasmic PTEN expression and relevant clinical, pathological and demographic variables within each histotype. For ordinal associations, only negative, weak and positive levels of PTEN expression were included.

The gCMH test is equivalent to the Pearson’s Chi-Square test for nominal–nominal associations, to the extended Cochran–Armitage test for nominal–ordinal associations and to the linear-by-linear association test for ordinal–ordinal associations.^19^ We assigned equidistant scores for ordinal levels. We used Holm’s multiplicity correction, valid under arbitrary assumptions, to achieve a global type I error of 5% per analysis and histotype. All *p* values presented in the manuscript are adjusted for multiplicity correction.

Significant associations were visualised using mosaic plots, which represent the different contingency table cells, in which the area of the tiles is proportional to the observed frequencies. Each tile is coloured depending on its contribution to the Pearson’s Chi-square statistics. For example, blue and red tiles correspond, respectively, to the cells showing frequencies smaller and greater than expected under the independence assumption. Further tests were performed to describe associations that were significant or clinically relevant.

In order to determine disease-specific survival associations with cytoplasmic PTEN expression, we used clinical data censored at 10 years from diagnosis and fitted Cox proportional hazard models stratified by site. With the exception of the cases with LGSOC, which had a small observed sample size, we controlled for age (and age squared), stage (four levels: I/II/III/IV), and post-operative residual tumour (2 levels: Yes/No). Survival times were left-truncated to obtain unbiased parameter estimates with delayed entries (refer to Section 3.5 of ref. ^20^ for detail), as well as right-censored for patients still alive after 10 years of follow-up or deceased owing to other causes. Deaths due to unknown causes were considered as disease related. We analysed the deviance and Schoenfeld residuals as model checks and performed sensitivity analyses by running the same analyses on data with imputed missing predictors^21^ and by controlling for the biomarkers CD8, ER, PR and AR independently or jointly (refer to Supplementary Material for detail). All IHC markers investigated were pre-specified before the analysis, and all statistical analyses were corrected for multiple testing. In the survival analyses, we used a multiplicity correction, which takes the dependence between the PTEN parameters of interest into account and allows for a global 5% type I error rate per histotype.^22^

Cohen’s *κ* and its weighted variant were, respectively, used to assess inter-rater reliability for categorical and ordinal outcomes, where −1, 0 and 1 represent complete disagreement, agreement by chance and complete agreement, respectively. The strength of association was described as recommended by Landis and Koch.^23^

## Results

### PTEN loss is prevalent across all histotypes of ovarian cancer

Classically PTEN acts as a cytoplasmic protein but is also expressed in the nucleus, where it may have several regulatory functions.^24^ In order to evaluate the reproducibility of both cytoplasmic and nuclear scoring, two observers compared 678 scores randomly assigned across all TMAs evaluated in this study (~4.4% of the original cohort). PTEN cytoplasmic scoring was reproducible between observers (Cohen’s weighted *κ* = 0.59 for the ‘negative’, ‘weak’ and ‘positive’ ordinal levels; 95% confidence interval (CI) 0.53–0.64) and inter-observer differences were mostly seen when assessing if a core had heterogeneous PTEN expression (Supplementary Fig. 2, Supplementary Table S2). Scoring of PTEN nuclear expression was less reproducible (Cohen’s weighted *κ* = 0.49 for the ‘0%’, ‘<10%’, ‘10–50%’ and ‘>50%’ ordinal levels; 95% CI 0.43–0.54). As PTEN is also infrequently expressed in the nucleus, only cytoplasmic staining was used for further analyses.

**Fig. 1:**
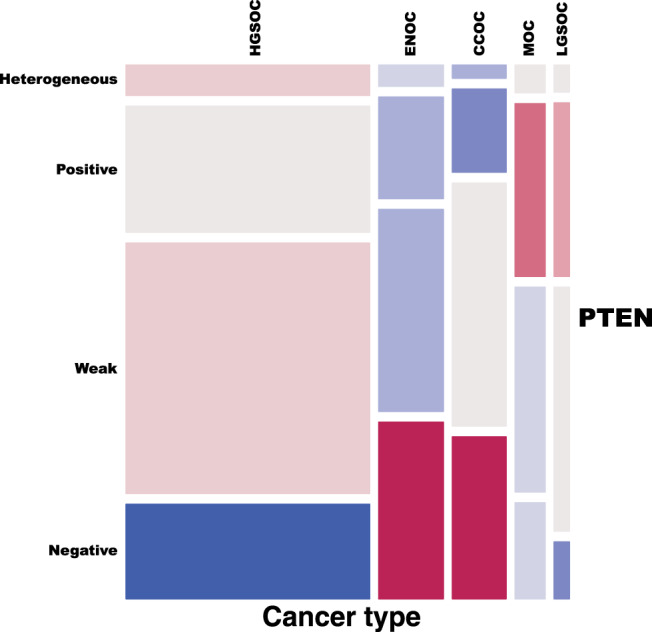
Prevalence of different levels of PTEN expression across histotypes of ovarian cancer. Mosaic representing the frequency of different scoring for PTEN expression using IHC per histotype with tiles of size proportional to their observed frequencies and colour coded according to their (signed) contributions to the Pearson’s Chi-square statistics. Blue and red tiles, respectively, correspond to cells showing frequencies smaller and greater than expected under the independence assumption. Refer to Supplementary Fig. 3 for the barplots of the absolute and relative frequency of the PTEN scores per histotype.

Loss of PTEN IHC cytoplasmic expression was statistically more frequent in endometrioid and clear cell histotypes, with 35% and 32% cases having complete absence of expression, respectively (Fig. 1, Supplementary Figure 3). In HGSOC, the proportion of PTEN loss or downregulation (scored as absent, weak or heterogeneous staining) was very similar to that observed in our previous smaller cohort (75% versus 77%, respectively).^15^

### Early clonal loss of PTEN is a common event and late sub-clonal loss of PTEN is more common in advanced stages in HGSOC

**Fig. 2:**
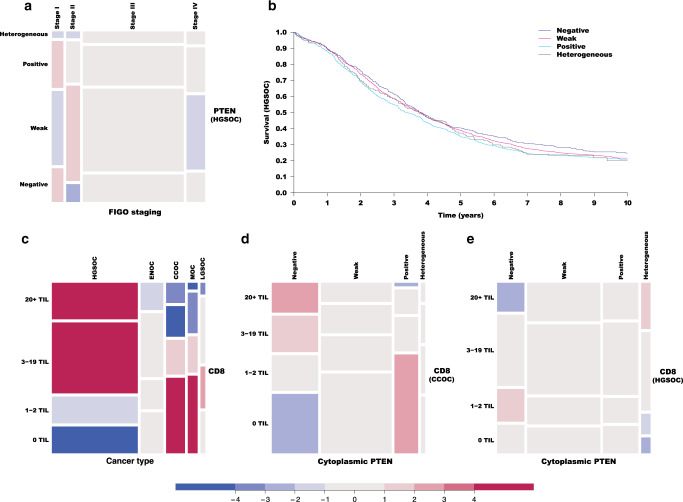
Association between levels of PTEN expression, stage, survival, and CD8 expression across ovarian cancer histotypes. **a** Mosaic plot corresponding to the association analysis between PTEN expression and stage of disease. **b** Kaplan–Meier survival curves for PTEN-negative, PTEN-positive, weak or heterogeneous staining for HGSOC from study cohort (multivariate hazard ratio 0.78, 95% CI 0.64–0.93, *N* = 1842, *p* = 0.0205 after multiplicity correction, when comparing PTEN negative vs PTEN positive). CI confidence interval. **c** Mosaic plot for the association between levels of CD8 expression and histotypes of ovarian cancer. Mosaic plots (**d**, **e**) summarise the association between PTEN expression and CD8 counts in CCOC and HGSOC, respectively. TIL tumour-infiltrating lymphocytes.

*PTEN* loss appears in pre-invasive lesions in the fallopian tube and therefore is a putative early driver in HGSOC.^25^ Our results show that loss of PTEN expression was similar across all stages of HGSOC (with the exception of moderately lower prevalence of PTEN negativity in stage II), consistent with previous evidence that PTEN is commonly lost early in tumour progression (Fig. 2a). Interestingly, heterogeneous expression of PTEN (suggestive of sub-clonal loss of PTEN) was more frequent in more advanced stages of the disease (from 4% in stage I to 8% in stage IV; gCMH *p* value = 0.0187 for a Cochran–Armitage trend test) and subgroup analysis shows that this association is present only in the context of absent BRCA1/2 pathogenic mutation (see Supplementary Information). In contrast to our previous reported results, loss of PTEN expression was not an adverse prognostic factor and had a modest positive effect on overall survival (hazard ratio (HR) 0.78, 95% CI 0.65–0.94; *p* value = 0.022 after multiplicity correction; Fig. 2b and Supplementary Table 3). Similar results were obtained with sensitivity analyses controlling for CD8, ER, PR and AR independently or jointly, suggesting that the effect of PTEN is additional to the effect of these biomarkers.

### HGSOC and CCOC show distinct associations between PTEN expression and CD8 counts

In HGSOC, CD8 counts were higher than in any other histotype of ovarian cancer (Fig. 2c). In preclinical models of melanoma, loss of PTEN was associated with decreased intra-tumour T cells.^4^ In this study, higher levels of PTEN expression were associated with lower CD8 counts in CCOC (gCHM test *p* value < 0.0001 for an ordinal/ordinal association; Fig. 2d and Supplementary Table 4). The average CD8 count per cytoplasmic PTEN level was also different in HGSOC (gCHM test *p* value = 0.0052 for a nominal/ordinal association, Fig. 2e and Supplementary Table 4). More strikingly, heterogeneous expression of PTEN in HGSOC (suggestive of sub-clonal loss of PTEN) was associated with significantly higher CD8 counts (gCHM test *p* value = 0.0016 for a binary/ordinal association corresponding to Cochrane–Armitage trend test; Fig. 2e).

### PTEN expression was strongly associated with AR, ER and PR expression in HGSOC

**Fig. 3:**
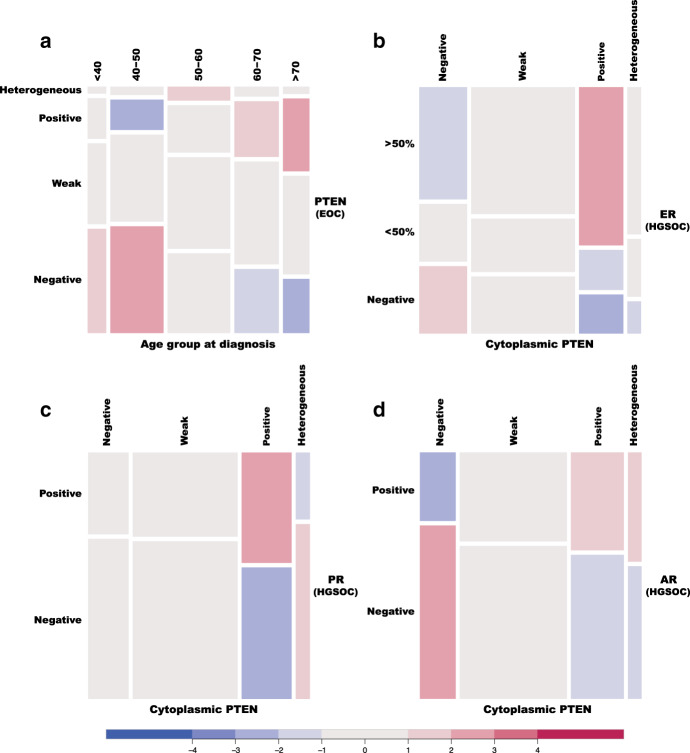
Association between levels of PTEN expression, age, and expression of hormonal receptors across ovarian cancer histotypes. **a** Mosaic plot summarising the association between PTEN expression and age groups in ENOC. **b**–**d** are mosaic plots summarising the association between levels of expression of PTEN and hormonal receptors (oestrogen receptor, progesterone receptor, and androgen receptor, respectively) in HGSOC.

In ENOC, PTEN loss was more prevalent in younger (<50 years) than in older patients (gCHM test *p* value < 0.0001 for an ordinal/ordinal association; Supplementary Table S3 and Fig. 3a), and this was likely related to the previously described hormonal regulation of PTEN expression. We also found that PTEN expression was strongly and positively associated with expression of ER, PR and AR in HGSOC (Supplementary Table 4; Fig. 3b–d).

## Discussion

The standard treatment of ovarian cancer has remained the same for the past two decades since the introduction of taxanes to platinum-based therapies, with very modest improvements in survival that are most likely related to improvements in supportive care. In order to define better therapeutic strategies, it is crucial to accurately describe the prevalence of common drivers and how they associate with clinical features and biomarkers of actionable pathways.^2^

Our large study of 5400 patients with ovarian cancer showed that complete loss of PTEN is highly prevalent across different histotypes of ovarian cancer ranging from 11% in LGSOC and 35% in CCOC, suggesting that it may be a driver of the disease. A small number of molecular alterations are generally sufficient to drive tumour initiation and progression.^26^ As HGSOC and its pre-cancerous lesions in the fallopian tubes have highly complex genomes^27–29^ and there is marked heterogeneity between patients, the identification of important drivers is challenging. Our results show a similar frequency of complete PTEN loss across all stages of HGSOC, which is consistent with loss of PTEN as a common early step in the progression of HGSOC. In BRCA1-associated breast cancer, which has molecular similarities with HGSOC (e.g., genomic instability, chromosomal complexity, frequent *TP53*, *BRCA1* and *BRCA2* mutation), PTEN loss was also the most frequent initiating event.^18^ Interestingly, we found here that the frequency of HGSOC cases with heterogeneous expression of PTEN (suggestive of a later sub-clonal loss of PTEN) is higher in more advanced disease in patients without any confirmed pathogenic *BRCA1/2* mutation. As stage is a surrogate for increased tumour mass, this suggests that PTEN loss confers a proliferative advantage to tumour cells and is positively selected over HGSOC cell expansion. PTEN loss is also one of the most frequent hits for increased cell viability in CRISPR screens.^30^ Considering the role of the PI3K pathway in the regulation of genomic stability, it is difficult to ascertain if loss of *PTEN* by chromosomal deletion is a result of baseline genomic instability or if it influences the latter.^31^ Therefore, despite the clinical and pathological associations we presented, it remains inconclusive if loss of PTEN is a driver of the disease initiation or progression. Previous evidence of PTEN loss in early pre-cancer lesions in the fallopian tubes,^25^ its role in the mouse models of HGSOC^12–14^ and high focal prevalence of PTEN loss compared to other neighbouring genes (Gistic *p* value for the likelihood of PTEN loss being a driver: 7.3e−17)^11,32^ in HGSOC are supportive of the role of PTEN loss in driving progression in HGSOC. Whole-genome characterisation of chemoresistant ovarian tumours also suggested that gene breakage of PTEN and other tumour-suppressor genes also contributes to chemoresistance.^33^

The majority of ENOC arise from malignant transformation of endometriosis, suggesting that pathways involved in the transformation of this ectopic endometrium into ENOC may overlap with the processes involved in the progression from normal endometrium to endometrial cancer.^34^ In this context, PTEN loss is commonly lost in the normal endometrium.^35^ In our study, the frequency of PTEN loss in ENOC was significantly higher in younger premenopausal patients whose higher levels of oestradiol and progesterone induce higher expression and phosphorylation of PTEN in the endometrium.^5^ This suggests that deletion of PTEN is therefore more likely to be positively selected and to provide a proliferative advantage in the context of a surrounding endometrium with upregulated PTEN than within the endometrium of older postmenopausal patient where lower hormonal levels already condition lower levels of PTEN expression. In this context, we found a strong association between expression of hormonal receptors (AR, ER and PR) and PTEN expression in HGSOC. Activity of the PTEN/PI3K pathway has also been associated with AR expression in prostate cancer,^20,36^ and mouse models suggest that a functional AR may increase PTEN inactivation-induced uterine cancer.^37^ These data suggest that PTEN inactivation is positively selected by functional androgen activity in these tumour types.

In a previous study, we found in two smaller cohorts that cases with any type of loss of PTEN expression was associated with shorter overall survival. Although this association was significant, the confidence intervals were relatively wide (*N* = 439; HR 1.5, 95% CI 1.1–2.0).^15^ In this much larger cohort, the frequency of loss was similar, but there was an opposite prognostic signal, reinforcing the need to use large cohorts for validation of putative prognostic biomarkers. The association between PTEN negative expression in HGSOC and longer overall survival (HR 0.78 95% CI 0.64–0.93) is counterintuitive considering that activation of the PI3K pathway is generally associated with proliferation and more aggressive tumours. We speculate that further genetic interactions may be important in determining survival. For instance, if PTEN loss appears stochastically in the context of existing homologous recombination deficiency, accelerated proliferation on a background chromosomal instability may increase genomic crisis and cell death. In addition, loss of PTEN may have treatment interactions particularly with platinum-based chemotherapy as higher proliferation and genomic instability predict better response.^38^

A multi-omics approach also demonstrated that immune-infiltrate of cytotoxic T cells can be recruited to the tumour by cytokines derived from the activation of the DNA damage response protein ATM in tumours that are genomically unstable and therefore enriched for chromosomal copy number alterations (CNAs),^39^ supporting previous associations between BRCA-like HGSOC (characterised by homologous recombination deficiency) and immune infiltrates.^11,40^ In previous studies, CD8 counts were indeed highest in HGSOC (the ovarian cancer histotype with higher prevalence of CNAs) as compared to other histotypes, and higher CD8 counts were associated with longer survival in HGSOC and ENOC.^16^ Despite the associations we found between expression of PTEN and CD8 counts in HGSOC, they seem to have opposite effects on survival. More specifically, PTEN loss and higher CD8 counts seem to have positive effects on prognosis, which may be explained by their association with CNA-enriched, genomically unstable tumours. This is further supported by the fact that the highest CD8 counts were found in tumours with heterogeneous expression of PTEN (and likely late sub-clonal loss of PTEN).

Preclinical murine models of melanoma suggest that PTEN loss promotes resistance to T cell-mediated immunotherapy by decreasing T cell trafficking into the tumours.^4^ Moreover, trials of PI3Kbeta inhibitor in this model improved the efficacy of both anti-PD-1 and anti-CTLA-4 antibodies. Recent in silico and IHC analysis of human prostate tumours showed that PTEN loss in primary prostate tumours was also associated with lower CD8+ TIL counts and an immunosuppressive tumour environment mediated by infiltrating FoxP3+ T cells.^41^ However, in the same study, PTEN-deficient metastasis to the lymph nodes showed increased counts of CD8+ T cells. Interestingly, these opposite associations are also demonstrated in our study, since PTEN loss was associated with low counts of CD8+ T cells in HGSOC cases and high counts in CCOC cases, supporting the hypothesis that not only PI3K activation may influence recruitment of cytotoxic T cells but also that these processes may be distinctly regulated in different subtypes of ovarian cancer.

PTEN loss is known to preferentially activate the PI3Kbeta subunit and we show that PTEN loss is a common event in all types of ovarian cancer and can appear in the context of chromosomal instability in HGSOC. These results suggest that combinations of small molecule inhibitors targeting the PI3K/AKT/mammalian target of rapamycin pathway (including inhibitors of the beta subunit), poly ADP-ribose polymerase or apoptotic pathways may have effective results in cases of HGSOC in the clinical setting. Preclinical studies using combinations of those agents have already shown promising results.^42–44^ Moreover, the associations we found between expression of PTEN and hormonal receptors suggest that molecular stratification based on expression of PTEN and hormonal receptors may be informative for tumours that are more likely to respond to hormonal therapies (high expression of PTEN and hormonal receptors) and those that could be preferentially treated with PI3Kbeta inhibitors (low expression of PTEN and hormonal receptors). Finally, future validation in primary cells from patients to assess if and how PTEN regulates recruitment of cytotoxic T cells may inform how the drug combinations above can be used to improve immunotherapy results. In summary, this work will inform future trials in ovarian cancer using combinations of immunotherapy and other targeted therapies and suggests that profiling for PTEN expression should also be done in the context of those trials.

## Supplementary information

Supplementary Material

Supplementary Figures and Tables

## Data Availability

The R markdown document containing the entire data set and allowing for reproducing all analyses performed in this manuscript is available as supplementary files.
