# Supplementary material for: Clinical and pathological associations of PTEN expression in ovarian cancer: a multicentre study from the Ovarian Tumour Tissue Analysis Consortium
Source: Br J Cancer. 2020 Jun 18;123(5):793–802. doi: 10.1038/s41416-020-0900-0 (PMC7463007; doi:10.1038/s41416-020-0900-0)
Supplement: Supplementary file 1 — Supplementary Material [file 41416_2020_900_MOESM1_ESM.pdf]

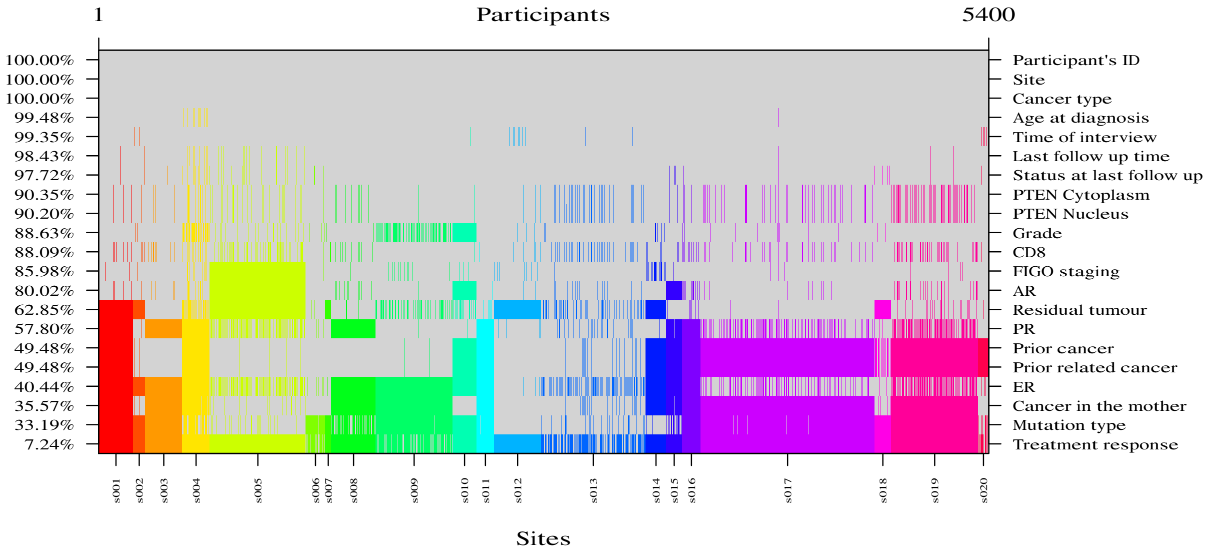

Supplementary Figure 1 **Missing data pattern in the OTTA dataset.** Missing data per variable ordered by missingness level (y-axis) for participants ordered by site (x-axis). Available data appear in grey. Missing data are color coded according to the participant site, highlighting that missing data is site-dependent, with some sites having few or no AR, PR, ER or FIGO measures.

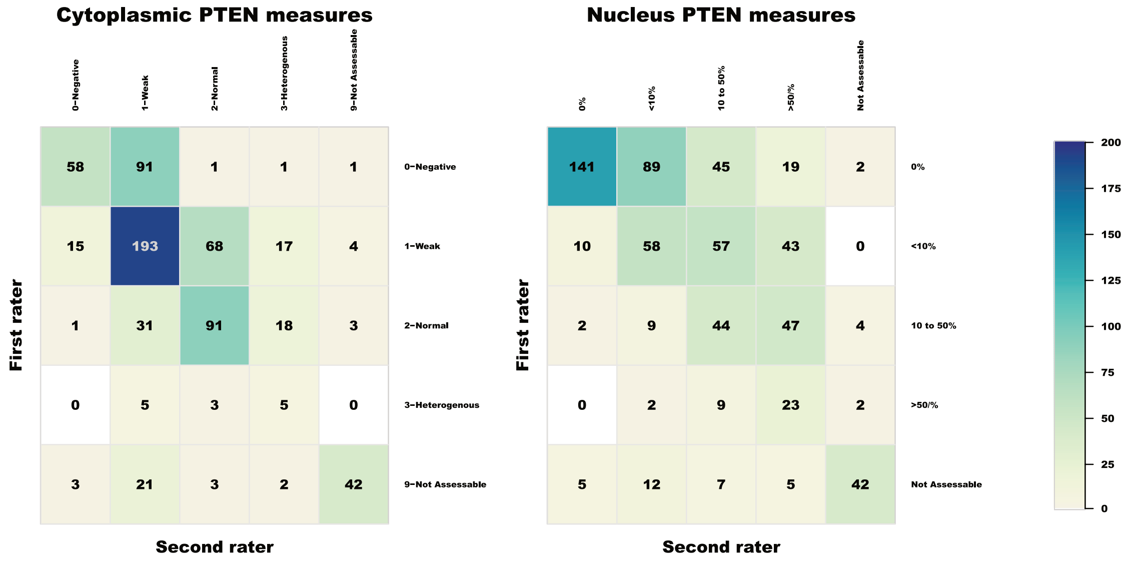

Cytoplasmic PTEN measures (rows: First rater; columns: Second rater)

	0−Negative	1−Weak	2−Normal	3−Heterogenous	9−Not Assessable
0−Negative	58	91	1	1	1
1−Weak	15	193	68	17	4
2−Normal	1	31	91	18	3
3−Heterogenous	0	5	3	5	0
9−Not Assessable	3	21	3	2	42

Nucleus PTEN measures (rows: First rater; columns: Second rater)

	0%	<10%	10 to 50%	>50/%	Not Assessable
0%	141	89	45	19	2
<10%	10	58	57	43	0
10 to 50%	2	9	44	47	4
>50/%	0	2	9	23	2
Not Assessable	5	12	7	5	42

Supplementary Figure 2 **Comparison of the cytoplasmic and nuclei PTEN scoring of 2 raters**

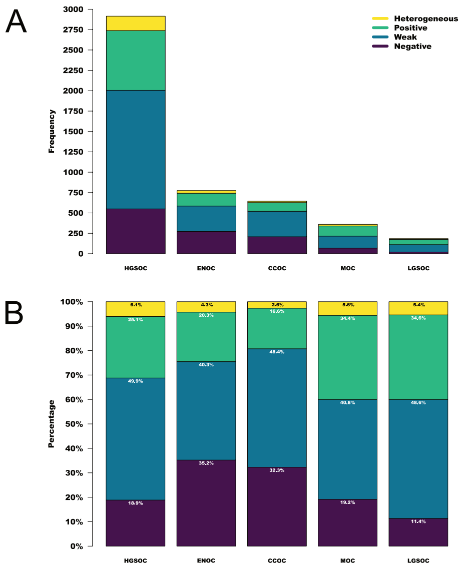

Supplementary Figure 3 **Prevalence of different levels of PTEN expression across histotypes of ovarian cancer.** (A) Frequency of different scoring for PTEN expression using IHC per histotype, (B) Proportion of PTEN scoring per histotype.

		Number of person-years (% per cancer subtype)					
		Cancer type					
		HGSOC	ENOC	CCOC	MOC	LGSOC	Total
	Total	14252 (100)	6227 (100)	4871 (100)	2616 (100)	1264 (100)	29230 (100)
Status at follow up	Data available	14052 (100)	6140 (100)	4780 (100)	2597 (100)	1242 (100)	28811 (100)
	Alive	6538 (47)	4874 (79)	3670 (77)	2157 (83)	808 (65)	18047 (63)
	Dead (disease)	5326 (38)	475 (8)	454 (9)	126 (5)	300 (24)	6681 (23)
	Dead (treatment)	11 (0)	23 (0)	15 (0)	0 (0)	1 (0)	50 (0)
	Dead (other)	586 (4)	322 (5)	225 (5)	126 (5)	16 (1)	1275 (4)
	Dead (unknown)	1591 (11)	446 (7)	416 (9)	188 (7)	117 (9)	2758 (10)
FIGO stage	Data available	12047 (100)	4711 (100)	3862 (100)	1967 (100)	985 (100)	23572 (100)
	Stage I	1501 (12)	2678 (57)	2275 (59)	1623 (83)	299 (30)	8376 (36)
	Stage II	1680 (14)	1375 (29)	1165 (30)	166 (8)	126 (13)	4512 (19)
	Stage III	7723 (64)	627 (13)	396 (10)	167 (8)	520 (53)	9433 (40)
	Stage IV	1143 (9)	31 (1)	26 (1)	11 (1)	40 (4)	1251 (5)
Differentiation	Data available	13128 (100)	5956 (100)	3120 (100)	2468 (100)	1234 (100)	25906 (100)
	Well	0 (0)	2652 (45)	155 (5)	1125 (46)	1085 (88)	5017 (19)
	Moderate	1588 (12)	2021 (34)	538 (17)	1090 (44)	8 (1)	5245 (20)
	Poor/None	11540 (88)	1283 (22)	2427 (78)	253 (10)	141 (11)	15644 (60)
Residual tumour	Data available	9025 (100)	3336 (100)	2902 (100)	1403 (100)	682 (100)	17348 (100)
	yes	3986 (44)	301 (9)	253 (9)	143 (10)	235 (34)	4918 (28)
	no	5039 (56)	3035 (91)	2649 (91)	1260 (90)	447 (66)	12430 (72)
Cytoplasmic PTEN	Data available	12857 (100)	5746 (100)	4500 (100)	2271 (100)	1060 (100)	26434 (100)
	negative	2770 (22)	2174 (38)	1462 (32)	452 (20)	131 (12)	6989 (26)
	weak	6314 (49)	2240 (39)	2248 (50)	881 (39)	505 (48)	12188 (46)
	positive	3043 (24)	1131 (20)	720 (16)	808 (36)	354 (33)	6056 (23)
	heterogeneous	730 (6)	201 (3)	70 (2)	130 (6)	70 (7)	1201 (5)
Nuclear PTEN	Data available	12817 (100)	5740 (100)	4485 (100)	2271 (100)	1060 (100)	26373 (100)
	0%	5475 (43)	3749 (65)	2168 (48)	1246 (55)	438 (41)	13076 (50)
	]0,10]%	3227 (25)	976 (17)	1013 (23)	422 (19)	299 (28)	5937 (23)
	]10,50]%	3101 (24)	789 (14)	875 (20)	370 (16)	272 (26)	5407 (21)
	]50,100]%	1014 (8)	226 (4)	429 (10)	233 (10)	51 (5)	1953 (7)
CD8 count	Data available	12734 (100)	5613 (100)	4396 (100)	2087 (100)	842 (100)	25672 (100)
	0 TIL	1791 (14)	1428 (25)	2120 (48)	939 (45)	220 (26)	6498 (25)
	1-2 TIL	2006 (16)	854 (15)	825 (19)	467 (22)	229 (27)	4381 (17)
	3-19 TIL	5619 (44)	2392 (43)	935 (21)	595 (29)	344 (41)	9885 (39)
	20+ TIL	3318 (26)	939 (17)	516 (12)	86 (4)	49 (6)	4908 (19)
AR expression	Data available	10808 (100)	4608 (100)	3664 (100)	1886 (100)	900 (100)	21866 (100)
	Negative	6772 (63)	3111 (68)	3454 (94)	1825 (97)	525 (58)	15687 (72)
	Positive	4036 (37)	1497 (32)	210 (6)	61 (3)	375 (42)	6179 (28)
PR expression	Data available	7897 (100)	4743 (100)	3910 (100)	1810 (100)	535 (100)	18895 (100)
	Negative	4904 (62)	1169 (25)	3645 (93)	1563 (86)	207 (39)	11488 (61)
	1-50 pos	2213 (28)	809 (17)	168 (4)	152 (8)	145 (27)	3487 (18)
	50+ pos	780 (10)	2765 (58)	97 (2)	95 (5)	183 (34)	3920 (21)
ER expression	Data available	6425 (100)	3155 (100)	2852 (100)	1209 (100)	446 (100)	14087 (100)
	Negative	1519 (24)	714 (23)	2494 (87)	986 (82)	79 (18)	5792 (41)
	1-50 pos	1524 (24)	561 (18)	126 (4)	61 (5)	72 (16)	2344 (17)
	50+ pos	3382 (53)	1880 (60)	232 (8)	162 (13)	295 (66)	5951 (42)

Table S1 Summary of the demographics of the study cohort stratified by cancer histotype.

	Cytoplasmic PTEN		Nuclei PTEN	
	**Estimate**	**95% CI**	**Estimate**	**95% CI**
**Global**	0.386	[0.332;0.439]	0.301	[0.256;0.347]
**Ordinal (weighted kappa)**	0.587	[0.534;0.636]	0.486	[0.430;0.541]
**Heterogenous vs homogenous**	0.157	[0.024;0.305]		
**Assessable versus non-assessable**	0.665	[0.561;0.763]	0.665	[0.561;0.763]

Table S2 Cohen's κ agreement coefficients for the cytoplasmic and nucleus measures when considering PTEN as nominal, ordinal, or when focusing on a given characteristic (Heterogenous, Assessable).

Variable	Test	HGSOC	ENOC	CCOC	MOC	LGSOC
**Tumour after treatment**	**Nominal/Nominal**	0.0521	1.0000	1.0000	0.1858	0.1983
	**Nominal/Ordinal**	0.0536	1.0000	1.0000	0.2058	0.1658
	**Ordinal/Ordinal**	1.0000	0.9175	0.8368	0.0978	0.1924
**Differentiation level**	**Nominal/Nominal**	0.0933	0.7317	1.0000	0.7987	1.0000
	**Nominal/Ordinal**	0.1400	0.5854	1.0000	0.8827	1.0000
	**Ordinal/Ordinal**	0.1051	0.6615	1.0000	1.0000	1.0000
**FIGO staging**	**Nominal/Nominal**	0.0521	1.0000	0.6228	0.7987	1.0000
	**Nominal/Ordinal**	0.2522	1.0000	0.3459	0.8827	1.0000
	**Ordinal/Ordinal**	1.0000	0.9175	0.1897	0.5556	1.0000
**Age group at diagnosis**	**Nominal/Nominal**	0.0933	0.0115	1.0000	0.7987	1.0000
	**Nominal/Ordinal**	0.2522	0.0001	1.0000	1.0000	1.0000
	**Ordinal/Ordinal**	0.7598	<0.0001	1.0000	1.0000	1.0000
**CD8**	**Nominal/Nominal**	0.0521	0.6474	0.0098	0.7987	1.0000
	**Nominal/Ordinal**	0.0052	0.1797	0.0001	1.0000	0.3630
	**Ordinal/Ordinal**	0.3074	0.0581	<0.0001	1.0000	1.0000
**AR**	**Nominal/Nominal**	0.0008	0.0587	1.0000	0.4956	1.0000
	**Nominal/Ordinal**	0.0007	0.0587	1.0000	0.5782	1.0000
	**Ordinal/Ordinal**	0.0008	0.0050	0.9603	0.5556	0.4657
**PR**	**Nominal/Nominal**	0.0114	1.0000	1.0000	0.1858	0.7482
	**Nominal/Ordinal**	0.0330	1.0000	1.0000	0.8827	0.1605
	**Ordinal/Ordinal**	0.0621	0.9175	0.8368	0.5556	0.0640
**ER**	**Nominal/Nominal**	0.0026	1.0000	1.0000	0.7987	0.1983
	**Nominal/Ordinal**	0.0004	0.4250	0.4617	0.8827	0.0264
	**Ordinal/Ordinal**	0.0002	0.4679	0.2290	0.5556	0.0125

Table S3 For multiplicity adjusted p-values of generalised Cochran-Mantel-Haenszel tests analysing the association between Cytoplasmic PTEN and different (ordinal) factors. Such tests correspond to (i) Pearson's Chi-Square tests when analysing the association between two nominal variables, (ii) extended Cochran-Armitage tests when analysing the association between a nominal and an ordinal variable, (iii) linear-by-linear association tests when analysing the relationship between two ordinal variables. Cytoplasmic PTEN is considered as a nominal variable in the 'nominal/nominal' and 'nominal/ordinal' analyses and is considered as ordinal when discarding the category 'heterogenous' in the 'ordinal/ordinal' analyses. The used Holm multiplicity correction allows to achieve a 5% global type I error at the cancer-type and analysis ('nominal/nominal', 'nominal/ordinal' and 'ordinal/ordinal') level.

		Hazard ratio			Inference	
**Histotype**	**PTEN Level**	**Low**	**Mid**	**High**	***p*-value**	**Sig.**
**HGSOC**	Heterogeneous	0.751	0.953	1.208	0.9628	
	Weak	0.806	0.925	1.061	0.5629	
	Negative	0.651	0.781	0.937	0.0219	*
**ENOC**	Heterogeneous	1.132	3.239	9.262	0.0733	.
	Weak	0.636	1.176	2.174	0.9147	
	Negative	0.843	1.582	2.968	0.3411	
**CCOC**	Heterogeneous	0.5	1.473	4.341	0.8183	
	Weak	0.544	0.867	1.382	0.8754	
	Negative	0.567	0.929	1.523	0.9822	
**MOC**	Heterogeneous	0.521	1.604	4.942	0.7636	
	Weak	0.684	1.278	2.387	0.7965	
	Negative	0.195	0.472	1.145	0.2406	
**LGSOC**	Heterogeneous	0.317	0.82	2.121	0.9635	
	Weak	0.529	0.863	1.409	0.9024	
	Negative	0.75	1.446	2.786	0.5886	

Table S4 Hazard ratio estimates, 95% CI and for-multiplicity-corrected p-values of Cox proportional hazard models fitted on the complete cases of the OTTA dataset per histotype. The outcome of interest is the disease-specific survival within 10 years of diagnosis with ovarian cancer. Survival times were considered as left-truncated due to delayed entries, as well as right-censored for patients still alive at time of last follow-up or dead due to other causes. For all histotypes except LGSOC, we controlled for the variables age, stage, grade and presence of residual disease post-surgery. Analyses were stratified by site. The used multiplicity correction takes the dependence between the PTEN parameters of interest into account and allows to get a global 5% type I error per histotype.

Script 1 - Importation

D.-L. Couturier / F. Martins / J. Brenton / P. Pharoah

Last modified: 13 Mar 2020

```
#   Input data files  :    data/otta_noid.csv
#                          data/pten_scores_rater1.csv
#                          data/pten_scores_rater2.csv
#
#   Output data files :    results/rdata/1-raters.rd
#                          results/rdata/1-otta-raw.rd
#                          results/rdata/1-otta-amended.rd
#                          results/tables/1-otta-amended.csv
#                          results/tables/1-otta-raw.csv
#                          results/tables/1-otta-samplesize-participants.csv
#                          results/tables/1-otta-samplesize-personyears.csv
#
#   Required R packages :  colorspace, psych
```

1 OTTA datset

In this section, we import the raw **OTTA** dataset, prepare the relevant variables and propose a few descriptive analyses.

1.1 Import the raw OTTA dataset

```
OTTA = read.csv(file="data/otta_noid.csv", header=TRUE, stringsAsFactors=FALSE)
rownames(OTTA) = OTTA$OTTA.ID

# correct coding of missing for variable ca125_pretx
OTTA[,"ca125_pretx"][OTTA[,"ca125_pretx"]==""] = NA

# print sample
head(OTTA)
```

The dataset has 6208 rows (patients) and 76 columns (variables).

1.2 Prepare relevant variables

The code of the following sections creates the data.frame **otta**, containing the information required for further analyses.

```
otta = data.frame(pos=1:nrow(OTTA), id=OTTA$OTTA.ID, site = factor(OTTA$site))
```

1.2.1 Cancer type

The analysis focuses on the *HGSOC, Endometrioid, Clear cell, Mucinous* , *LGSOC* cancer types. Here we define the 5-level factors *cancer_type* (abbreviations) *cancer_type2* (full names) and 6-level factor *cancer_type3* (with level *other*). The coding of this variable in the OTTA dataset is

- 1H = HGSOC,
- 1L = LGSOC,
- 1 = serous; unknown grade,
- 2 = mucinous,
- 3 = endometrioid,
- 4 = clear cell,
- 5 = mixed cell, 6=other specified epithelial ovarian cancer (e.g. Brenner),
- 7 = undifferentiated/poorly differentiated epithelial,
- 8 = unknown, but known to be epithelial,
- 9 = Non-epithelial, 0=Other (use when 6, 7, or 9 is not distinguished) or unknown if epithelial,
- 99 = serous LMP 999=not OC; 77=normal Fallopian tube; 888=borderline tumor with microinvasion,
- 88 = DK.

```
otta$cancer_type = factor(OTTA$Histology_Revised,levels=c("1H","3","4","2","1L"),
                          labels=c("HGSOC","EOC","CCOC","MOC","LGSOC"))
otta$cancer_type2 = factor(OTTA$Histology_Revised,levels=c("1H","3","4","2","1L"),
                          labels=c("HGSOC","Endometrioid","Clear cell","Mucinous","LGSOC"))
    # checks
    table(otta$cancer_type,otta$cancer_type2,useNA="always")
```

```
##
##          HGSOC Endometrioid Clear cell Mucinous LGSOC <NA>
##    HGSOC  3244            0          0        0     0    0
##    EOC       0          840          0        0     0    0
##    CCOC      0            0        693        0     0    0
##    MOC       0            0          0      405     0    0
##    LGSOC     0            0          0        0   218    0
##    <NA>      0            0          0        0     0  808
```

1.2.2 PTEN cytoplasmic scoring

The PTEN cytoplasmic scoring are available in the column *PTEN_MAX* of the **OTTA** dataset with following levels

- 0 = negative,
- 1 = weak staining,
- 2 = normal intensity staining ie equivalent to the stroma,
- 3 = heterogeneous staining,
- 9 = not assessable.

It can be deduced from the columns *PTEN_1* to *PTEN_12* by means of the function `pten_cyto_i.fun()` as follows

```
pten_cyto.i = apply(apply(OTTA[,paste0("PTEN_",1:12)],2,function(x)as.numeric(substr(x,1,1))),
                    1,pten_cyto_i.fun)
    # comment: warnings correspond to NAs
```

A look at the differences between the two vectors of scores suggests that the scoring of the column *PTEN_MAX* is not always consistent so that the other ones are preferred.

```r
table(OTTA$PTEN_MAX,pten_cyto.i,useNA="always")# [ = Table 2 of report]
```

```
##       pten_cyto.i
##           0    1    2    3    9 <NA>
##   0    1269    0    1    2    0    0
##   1       1 2666    4    3    0    0
##   2       0    0 1397   16    0    0
##   3       0    0    0  271    0    0
##   9       1    0    0    0  577    0
##   <NA>    0    0    0    0    0    0
```

```r
    differences = cbind(dlc=pten_cyto.i,
                        otta=OTTA$PTEN_MAX,OTTA[,paste0("PTEN_",1:12)])[pten_cyto.i!=OTTA$PTEN_MAX,]
    colnames(differences) = c("new","PTEN_MAX",paste0("Core",1:12))
    differences
```

We defined the following 4-, 3- and 2-level factors

```r
otta$pten4_cytoplasm = factor(pten_cyto.i,levels=c(0,1,2,3),
                              labels=c("Negative","Weak","Positive","Heterogeneous"))
otta$pten3_cytoplasm = factor(c(1,1,2,3)[as.numeric(otta$pten4_cytoplasm)],levels=c(1,2,3),
                              labels=c("Non-positive","Positive","Heterogeneous"))
otta$pten2_cytoplasm = factor(c(1,1,2,1)[as.numeric(otta$pten4_cytoplasm)],levels=c(1,2),
                              labels=c("Other","Positive"))
table(otta$cancer_type2,otta$pten4_cytoplasm,useNA="always")
```

```
##
##                 Negative Weak Positive Heterogeneous <NA>
##   HGSOC              550 1455      733           177  329
##   Endometrioid       273  312      157            33   65
##   Clear cell         208  312      107            17   49
##   Mucinous            69  147      124            20   45
##   LGSOC               21   90       64            10   33
##   <NA>               150  350      217            35   56
```

The left (**Figure 1A of the article**) and right (**Figure 1B of the article**) plots of the following figure respectively show the absolute and relative number of cases per level of the variable *pten4_cytoplasm* and cancer type.

```r
mx.freq.ptenXtype = sapply(split(otta$pten4_cytoplasm,otta$cancer_type),table)
mx.pc.ptenXtype   = apply(mx.freq.ptenXtype,2,function(x)x/sum(x)*100)
barplotw          = barplot(mx.freq.ptenXtype,plot=FALSE)
nlevelw           = nlevels(otta$pten4_cytoplasm)
par(mfrow=c(1,2),mar=c(2.5,5,1,0))
# absolute
barplot(mx.freq.ptenXtype,axes=FALSE,ylim=c(0,3250),cex.names=.75,
        ylab="",col=col1.fun(nlevelw))
axis(2,seq(0,3250,250),las=2)
axis(2,3000/2,"Frequency",tick=FALSE,padj=-5)
legend("topright",legend=levels(otta$pten4_cytoplasm)[nlevelw:1],lwd=4,
       col=col1.fun(nlevelw)[nlevelw:1],lty=1,box.lwd=NA)
# relative
barplot(mx.pc.ptenXtype,axes=FALSE,ylim=c(0,100),cex.names=.75,
        ylab="",col=col1.fun(nlevelw))
axis(2,seq(0,100,10),paste0(seq(0,100,10),"%"),las=2)
axis(2,50,"Percentage",tick=FALSE,padj=-5)
```

```
formatw = matrix(paste0(format(round(mx.pc.ptenXtype,1)),"%"),ncol=5)
for(i in 1:ncol(mx.pc.ptenXtype)){
    text(barplotw[i],cumsum(mx.pc.ptenXtype[,i])-1.25,
         formatw[,i],cex=.6,col=rep(c("white","black"),c(nlevelw-1,1)))
    }
```

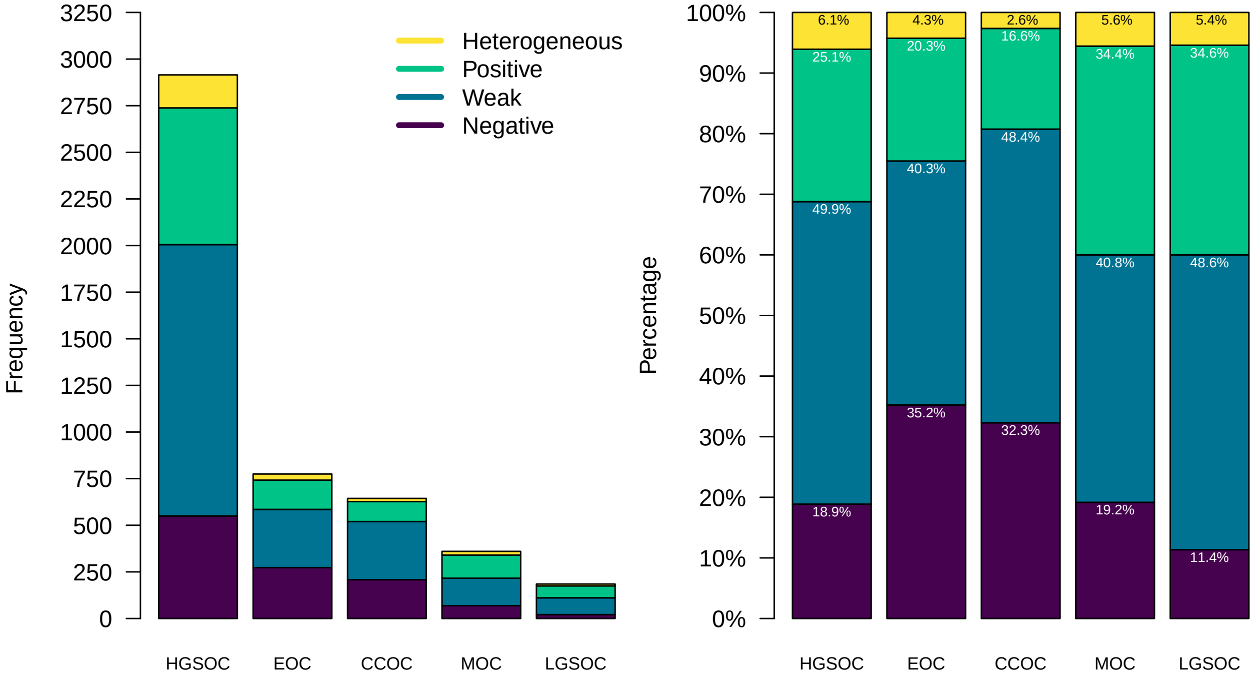

1.2.3 PTEN nucleus scoring

The proportion of the tumour nuclei with significant staining was coded as follows by raters

- 0 = none,
- 1 = $<$ 10%,
- 2 = 10-50%,
- 3 = $>$ 50%.

The PTEN nucleus scoring can be deduced from the columns *PTEN_1* to *PTEN_12* by means of the function `pten_nucl_i.fun()` as follows

```
pten_nucl.i = apply(apply(OTTA[,paste0("PTEN_",1:12)],2,extract.pten_nucl_c.fun),
                    1,pten_nucl_i.fun)
    # comment: warnings correspond to NAs
```

We defined the following 4-, and 3-level factors

```
otta$pten4_nucleus = factor(pten_nucl.i,levels=c(0,1,2,3),
                            labels=c("0%","]0,10]%","]10,50]%","]50,100]%"))
otta$pten3_nucleus = factor(c(1,1,2,3,9)[as.numeric(otta$pten4_nucleus)],levels=c(1,2,3),
                            labels=c("[0,10]%","]10,50]%","]50,100]%"))
table(otta$cancer_type,otta$pten4_nucleus,useNA="always")
```

```
##
##              0% ]0,10]% ]10,50]% ]50,100]% <NA>
```

```
##    HGSOC 1211      793       679        227  334
##    EOC    483      141       114         36   66
##    CCOC   288      153       146         56   50
##    MOC    171       77        72         39   46
##    LGSOC   68       52        53         12   33
##    <NA>   255      157       214        126   56
```

The left and right plots of the following figure respectively show the absolute and relative number of cases per level of the variable *pten4_nucleus* and cancer type.

```
mx.freq.ptenXtype = sapply(split(otta$pten4_nucleus,otta$cancer_type),table)
mx.pc.ptenXtype   = apply(mx.freq.ptenXtype,2,function(x)x/sum(x)*100)
barplotw          = barplot(mx.freq.ptenXtype,plot=FALSE)
nlevelw           = nlevels(otta$pten4_nucleus)
par(mfrow=c(1,2),mar=c(2.5,5,1,0))
# absolute
barplot(mx.freq.ptenXtype,axes=FALSE,ylim=c(0,3250),cex.names=.75,
        xlab="",ylab="",col=col1.fun(nlevelw))
axis(2,seq(0,3250,250),las=2)
axis(2,3000/2,"Frequency",tick=FALSE,padj=-5)
legend("topright",legend=levels(otta$pten4_nucleus)[nlevelw:1],lwd=4,
       col=col1.fun(nlevelw)[nlevelw:1],lty=1,box.lwd=NA)
# relative
barplot(mx.pc.ptenXtype,axes=FALSE,ylim=c(0,100),cex.names=.75,
        xlab="",ylab="",col=col1.fun(nlevelw))
axis(2,seq(0,100,10),paste0(seq(0,100,10),"%"),las=2)
axis(2,50,"Percentage",tick=FALSE,padj=-5)
formatw = matrix(paste0(format(round(mx.pc.ptenXtype,1)),"%"),ncol=5)
for(i in 1:ncol(mx.pc.ptenXtype)){
    text(barplotw[i],cumsum(mx.pc.ptenXtype[,i])-1.25,
         formatw[,i],cex=.6,col=rep(c("white","black"),c(nlevelw-1,1)))
    }
```

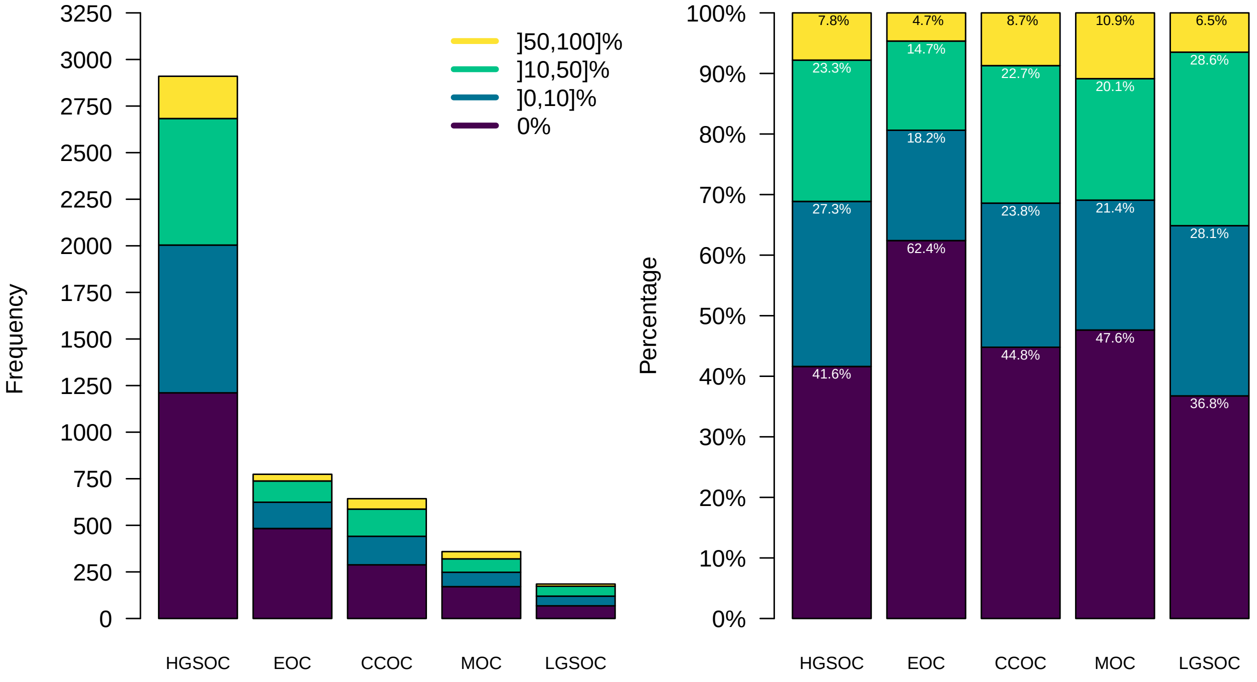

1.2.4 CD8

In the **OTTA** dataset, the information related to CD8 lies in 2 columns

- in column *CD8_MAX_s005* for site *s005*,
- in column *CD8_MAX* for other sites,

and was coded as follows

- 0 = no intraepithelial lymphocytes (IEL),
- 1 = 1-2 IEL/40 x HPF,
- 2 = 3-19 IEL/40 x HPF,
- 3 = 20 or more IEL/40 x HPF,
- 9 = Not representative (core should contain at least 25% tumor tissue).

The CD8 scores can also be deduced from the scores of the participant's cores of columns *CD8_1* to *CD8_12* by means of the function `cd8_i.fun()`.

A look at the differences between the two vectors of scores suggest that the scoring of the column *CD8_MAX* is not always consistent so that the other ones are preferred.

```
OTTA$CD8_MAX_COMBINED = OTTA[,"CD8_MAX"]
OTTA$CD8_MAX_COMBINED[!is.na(OTTA[,"CD8_MAX_s005"])] = OTTA[,"CD8_MAX_s005"][!is.na(OTTA[,"CD8_MAX_s005
cd8.i = apply(OTTA[,paste0("CD8_",1:12)],1,cd8_i.fun)
table(OTTA[,"CD8_MAX"],cd8.i,useNA="always")
```

```
##       cd8.i
##           0    1    2    3    9 <NA>
##   0    1170    7   18    8    0    0
##   1       0  854    0    0    0    0
##   2       0    0 1900    0    0    0
##   3       0    0    0  894    0    0
##   9       4    5    4    2  654    0
##   <NA>    0    0    0    0    0  688
```

```
# check differences
cbind(cd8.i,OTTA[,c("CD8_MAX",paste0("CD8_",1:12))])[cd8.i!=OTTA[,"CD8_MAX"]&!is.na(cd8.i),]
```

```
cd8.i[OTTA$site=="s005"] = OTTA[OTTA$site=="s005","CD8_MAX_s005"]
```

We defined the following 4-, and 2-level factors

```
otta$cd84 = factor(cd8.i,levels=c(0:3),
                   labels=c("0 IEL","1-2 IEL","3-19 IEL","20+ IEL"))
otta$cd82 = factor(c(1,1,2,2)[as.numeric(otta$cd84)],levels=c(1:2),
                   labels=c("0-2 IEL","3+ IEL"))
table(otta$cancer_type,otta$cd84,useNA="always")
```

```
##
##          0 IEL 1-2 IEL 3-19 IEL 20+ IEL <NA>
##   HGSOC    479     481     1278     655  351
##   EOC      193     136      301     128   82
##   CCOC     295     135      119      78   66
##   MOC      157      73       82      13   80
##   LGSOC     40      40       63      11   64
##   <NA>     182     105      253      87  181
```

The left and right plots of the following figure respectively show the absolute and relative number of cases per level of the variable *cd84* and cancer type.

```r
mx.freq.ptenXtype = sapply(split(otta$cd84,otta$cancer_type),table)
mx.pc.ptenXtype   = apply(mx.freq.ptenXtype,2,function(x)x/sum(x)*100)
barplotw          = barplot(mx.freq.ptenXtype,plot=FALSE)
nlevelw           = nlevels(otta$cd84)
par(mfrow=c(1,2),mar=c(2.5,5,1,0))
# absolute
barplot(mx.freq.ptenXtype,axes=FALSE,ylim=c(0,3250),cex.names=.75,
        xlab="",ylab="",col=col1.fun(nlevelw))
axis(2,seq(0,3250,250),las=2)
axis(2,3000/2,"Frequency",tick=FALSE,padj=-5)
legend("topright",legend=levels(otta$cd84)[nlevelw:1],lwd=4,
       col=col1.fun(nlevelw)[nlevelw:1],lty=1,box.lwd=NA)
# relative
barplot(mx.pc.ptenXtype,axes=FALSE,ylim=c(0,100),cex.names=.75,
        xlab="",ylab="",col=col1.fun(nlevelw))
axis(2,seq(0,100,10),paste0(seq(0,100,10),"%"),las=2)
axis(2,50,"Percentage",tick=FALSE,padj=-5)
formatw = matrix(paste0(format(round(mx.pc.ptenXtype,1)),"%"),ncol=5)
for(i in 1:ncol(mx.pc.ptenXtype)){
    text(barplotw[i],cumsum(mx.pc.ptenXtype[,i])-1.25,
         formatw[,i],cex=.6,col=rep(c("white","black"),c(nlevelw-1,1)))
    }
```

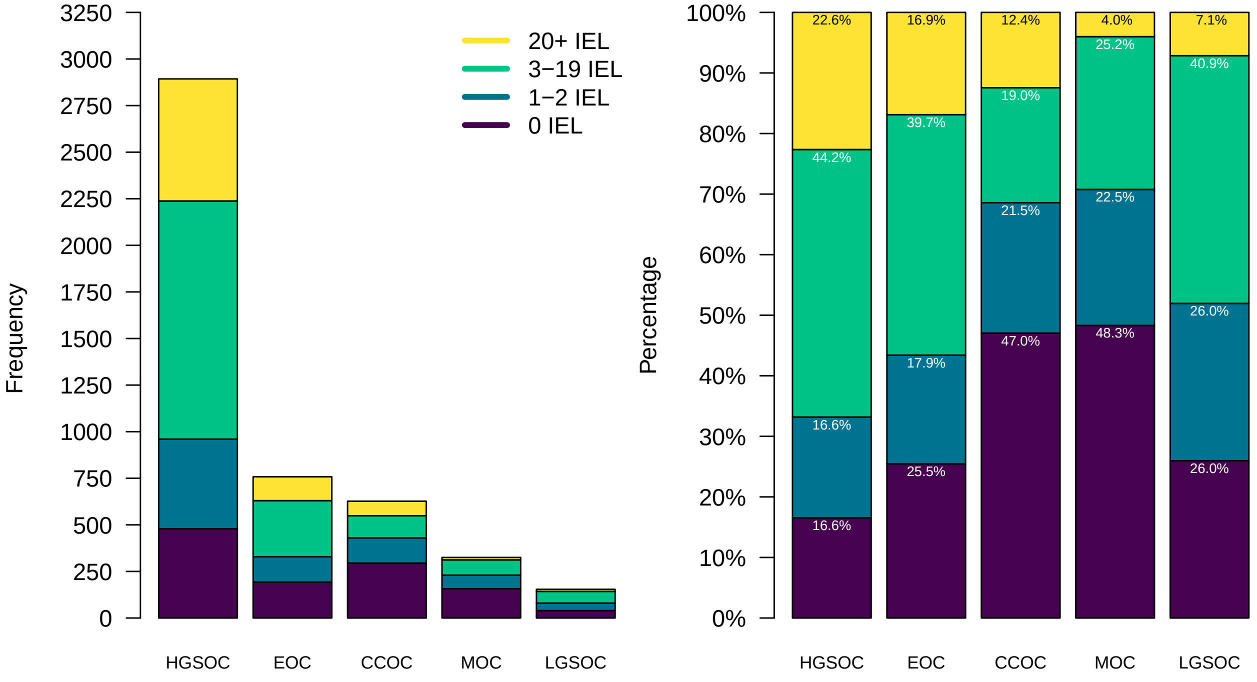

1.2.5 Androgen Receptor (AR)

The AR scores are available in the column *AR_MAX* and were coded as follows

- 0 = No Tissue, Non tumoural tissue, Necrosis, Hemorrhage,
- 1 = No staining in tumoral cells or just cytoplasmic staining (Negative),
- 2 = Just stromal cells staining (Positive),

- 3 = Just tumoral (epithelial) cells staining (Positive),
- 4 = Both tumoural and stromal cells staining(Positive).

Scores can also be deduced from the scores of the participant's cores of columns *AR_1* to *AR_12* by means of the function `ar_i.fun()`. A look at the small number of differences between the two vectors of scores suggest that the scoring of the column *AR_MAX* is not always consistent so that the other ones are preferred.

```
ar.i = apply(OTTA[,paste0("AR_",1:12)],1,ar_i.fun)
# check differences
table(OTTA[,"AR_MAX"],ar.i,useNA="always")
```

```
##       ar.i
##           0    1    2    3    4 <NA>
##   0     278    0    0    0    0    0
##   1       0 3317    0    0    0    0
##   2       0    0  222    0    0    0
##   3       0    0    0 1338    6    0
##   4       0    0    0    0  104    0
##   <NA>    0    0    0    0    0  943
```

```
cbind(ar.i,OTTA[,c("AR_MAX",paste0("AR_",1:12))])[ar.i!=OTTA[,"AR_MAX"]&!is.na(ar.i),]
```

We defined the following 4-, and 2-level factors

```
otta$ar4 = factor(ar.i,levels=c(1:4),labels=c("Negative","Stroma","Tumour","Both"))
otta$ar2 = factor(c(1,1,2,2)[as.numeric(otta$ar4)],levels=c(1:2),
                  labels=c("Negative","Positive"))
table(otta$cancer_type,otta$ar4,useNA="always")
```

```
##
##         Negative Stroma Tumour Both <NA>
##   HGSOC     1635     34    891   43  641
##   EOC        435     24    177   26  178
##   CCOC       503     29     23    9  129
##   MOC        258     47     10    2   88
##   LGSOC      105      4     62    4   43
##   <NA>       381     84    175   26  142
```

The left and right plots of the following figure respectively show the absolute and relative number of cases per level of the variable *ar2* and cancer type.

```
mx.freq.ptenXtype = sapply(split(otta$ar2,otta$cancer_type),table)
mx.pc.ptenXtype   = apply(mx.freq.ptenXtype,2,function(x)x/sum(x)*100)
barplotw          = barplot(mx.freq.ptenXtype,plot=FALSE)
nlevelw           = nlevels(otta$ar2)
par(mfrow=c(1,2),mar=c(2.5,5,1,0))
# absolute
barplot(mx.freq.ptenXtype,axes=FALSE,ylim=c(0,3250),cex.names=.75,
        xlab="",ylab="",col=col1.fun(nlevelw))
axis(2,seq(0,3250,250),las=2)
axis(2,3000/2,"Frequency",tick=FALSE,padj=-5)
legend("topright",legend=levels(otta$ar2)[nlevelw:1],lwd=4,
       col=col1.fun(nlevelw)[nlevelw:1],lty=1,box.lwd=NA)
# relative
barplot(mx.pc.ptenXtype,axes=FALSE,ylim=c(0,100),cex.names=.75,
        xlab="",ylab="",col=col1.fun(nlevelw))
axis(2,seq(0,100,10),paste0(seq(0,100,10),"%"),las=2)
```

```
axis(2,50,"Percentage",tick=FALSE,padj=-5)
formatw = matrix(paste0(format(round(mx.pc.ptenXtype,1)),"%"),ncol=5)
for(i in 1:ncol(mx.pc.ptenXtype)){
    text(barplotw[i],cumsum(mx.pc.ptenXtype[,i])-1.25,
         formatw[,i],cex=.6,col=rep(c("white","black"),c(nlevelw-1,1)))
    }
```

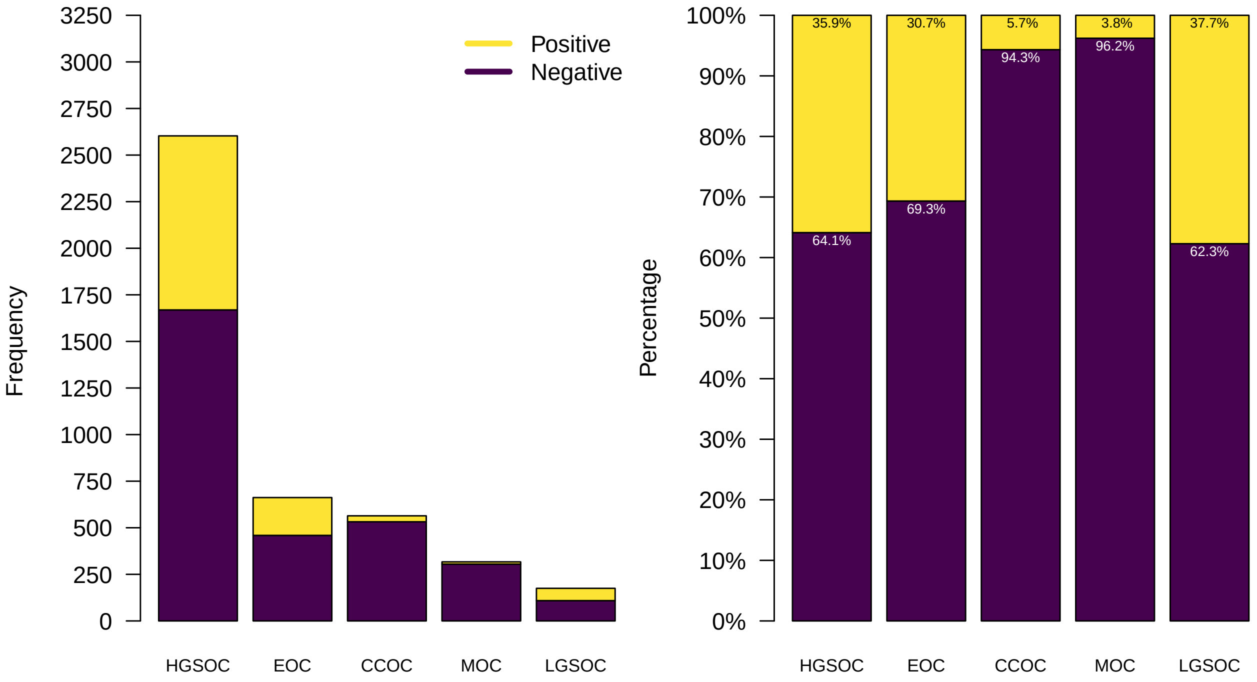

1.2.6 Progesterone Receptors (PR)

In the **OTTA** dataset, the information related to PR lies in the columns *PR* and *PR_2017_Max* and was coded as follows

- 0 = negative (< 1% of tumor cells),
- 1 = 1 to 50% of tumor cell nuclei positive,
- 2 = > 50% of tumor cells positive.

When both PR measures are available for a participant (52 cases only), they are often different (40%). Note that for 1256 participants, PR scores per cores are also available. Here we create a vector combining *PR* and *PR_2017_Max* and keep the information of *PR_2017_Max*, i.e., the most recent one, for participants with both measures.

```
OTTA$PR_COMBINED = OTTA$PR
OTTA$PR_COMBINED[!is.na(OTTA$PR_2017_Max)] = OTTA$PR_2017_Max[!is.na(OTTA$PR_2017_Max)]
```

We defined the following 3-, and 2-level factors

```
otta$pr3 = factor(c(0,1,2,rep(NA,20))[OTTA$PR_COMBINED+1],
                  levels=c(0:2),labels=c("Negative","1-50 pos.","50+ pos"))
otta$pr2 = factor(c(1,2,2)[as.numeric(otta$pr3)],levels=c(1:2),
```

```
                  labels=c("Negative","Positive"))
table(otta$cancer_type,otta$pr3,useNA="always")
```

```
##
##          Negative 1-50 pos. 50+ pos <NA>
##   HGSOC      1068       457     149 1570
##   EOC         167       111     312  250
##   CCOC        469        25      10  189
##   MOC         235        18      14  138
##   LGSOC        34        26      26  132
##   <NA>        238        85     120  365
```

The left and right plots of the following figure respectively show the absolute and relative number of cases per level of the variable *pr3* and cancer type.

```
mx.freq.ptenXtype = sapply(split(otta$pr3,otta$cancer_type),table)
mx.pc.ptenXtype   = apply(mx.freq.ptenXtype,2,function(x)x/sum(x)*100)
barplotw          = barplot(mx.freq.ptenXtype,plot=FALSE)
nlevelw           = nlevels(otta$pr3)
par(mfrow=c(1,2),mar=c(2.5,5,1,0))
# absolute
barplot(mx.freq.ptenXtype,axes=FALSE,ylim=c(0,3250),cex.names=.75,
        xlab="",ylab="",col=col1.fun(nlevelw))
axis(2,seq(0,3250,250),las=2)
axis(2,3000/2,"Frequency",tick=FALSE,padj=-5)
legend("topright",legend=levels(otta$pr3)[nlevelw:1],lwd=4,
       col=col1.fun(nlevelw)[nlevelw:1],lty=1,box.lwd=NA)
# relative
barplot(mx.pc.ptenXtype,axes=FALSE,ylim=c(0,100),cex.names=.75,
        xlab="",ylab="",col=col1.fun(nlevelw))
axis(2,seq(0,100,10),paste0(seq(0,100,10),"%"),las=2)
axis(2,50,"Percentage",tick=FALSE,padj=-5)
formatw = matrix(paste0(format(round(mx.pc.ptenXtype,1)),"%"),ncol=5)
for(i in 1:ncol(mx.pc.ptenXtype)){
    text(barplotw[i],cumsum(mx.pc.ptenXtype[,i])-1.25,
         formatw[,i],cex=.6,col=rep(c("white","black"),c(nlevelw-1,1)))
    }
```

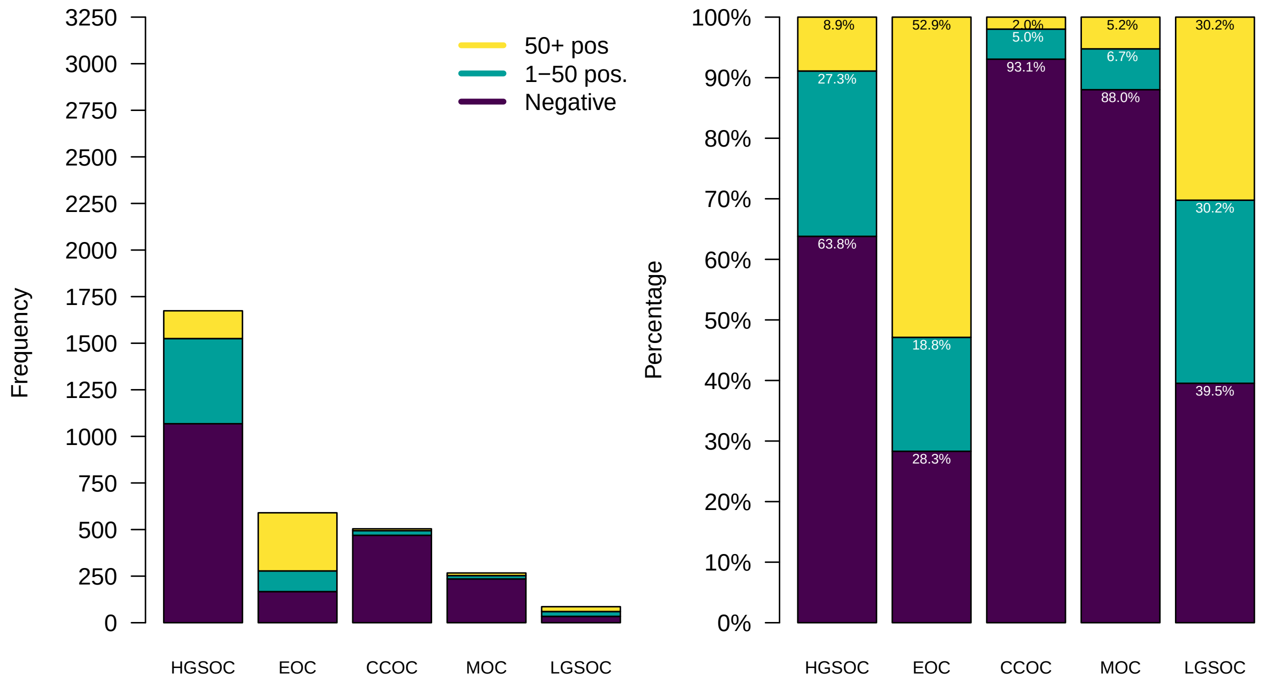

1.2.7 Oestrogen Receptors (ER)

The ER scores are in the column *ER* and were coded as follows

- 0 = negative (< 1% of tumor cells),
- 1 = 1 to 50% of tumor cell nuclei positive,
- 2 = > 50% of tumor cells positive.

We defined the following 3-, and 2-level factors

```
otta$er3 = factor(OTTA$ER,levels=c(0:2),
                  labels=c("Negative","1-50 pos.","50+ pos"))
otta$er2 = factor(c(1,2,2)[as.numeric(otta$er3)],levels=c(1:2),
                  labels=c("Negative","Positive"))
table(otta$cancer_type,otta$pr3,useNA="always")
```

```
##
##          Negative 1-50 pos. 50+ pos <NA>
##   HGSOC      1068       457     149 1570
##   EOC         167       111     312  250
##   CCOC        469        25      10  189
##   MOC         235        18      14  138
##   LGSOC        34        26      26  132
##   <NA>        238        85     120  365
```

The left and right plots of the following figure respectively show the absolute and relative number of cases per level of the variable *er3* and cancer type.

```
mx.freq.ptenXtype = sapply(split(otta$er3,otta$cancer_type),table)
mx.pc.ptenXtype   = apply(mx.freq.ptenXtype,2,function(x)x/sum(x)*100)
```

```
barplotw          = barplot(mx.freq.ptenXtype,plot=FALSE)
nlevelw           = nlevels(otta$er3)
par(mfrow=c(1,2),mar=c(2.5,5,1,0))
# absolute
barplot(mx.freq.ptenXtype,axes=FALSE,ylim=c(0,3250),cex.names=.75,
        xlab="",ylab="",col=col1.fun(nlevelw))
axis(2,seq(0,3250,250),las=2)
axis(2,3000/2,"Frequency",tick=FALSE,padj=-5)
legend("topright",legend=levels(otta$er3)[nlevelw:1],lwd=4,
       col=col1.fun(nlevelw)[nlevelw:1],lty=1,box.lwd=NA)
# relative
barplot(mx.pc.ptenXtype,axes=FALSE,ylim=c(0,100),cex.names=.75,
        xlab="",ylab="",col=col1.fun(nlevelw))
axis(2,seq(0,100,10),paste0(seq(0,100,10),"%"),las=2)
axis(2,50,"Percentage",tick=FALSE,padj=-5)
formatw = matrix(paste0(format(round(mx.pc.ptenXtype,1)),"%"),ncol=5)
for(i in 1:ncol(mx.pc.ptenXtype)){
    text(barplotw[i],cumsum(mx.pc.ptenXtype[,i])-1.25,
         formatw[,i],cex=.6,col=rep(c("white","black"),c(nlevelw-1,1)))
    }
```

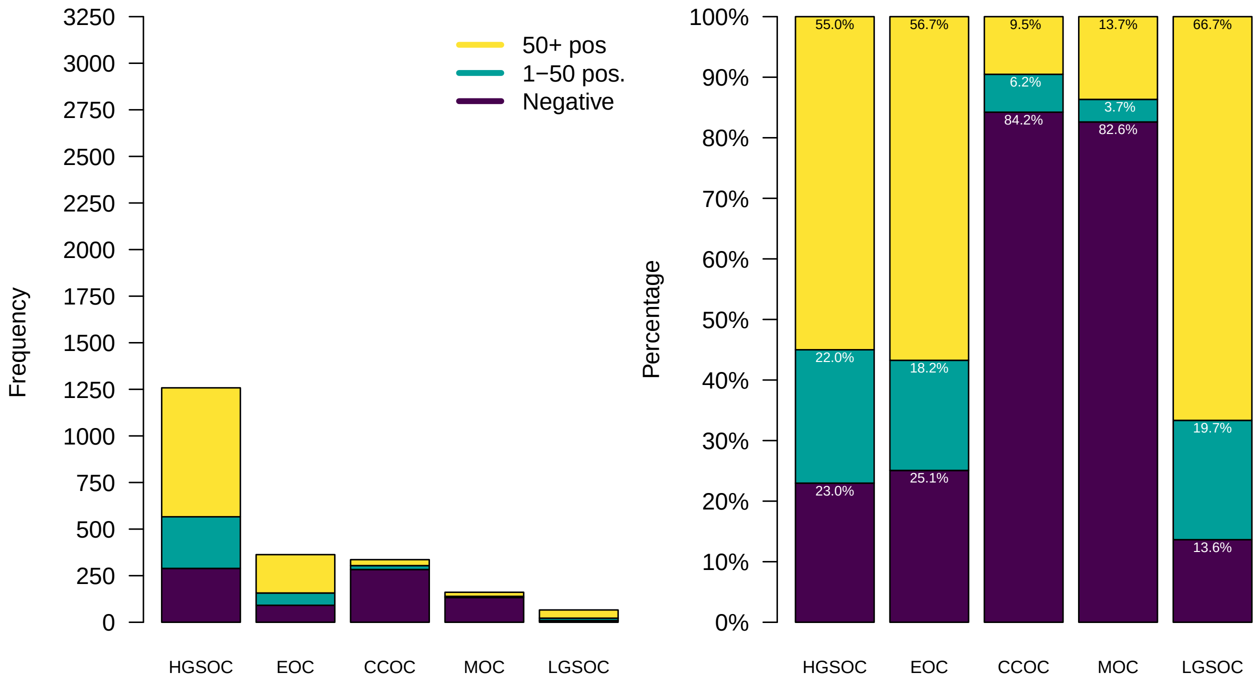

1.2.8 Response to treatment

Response to treatment was defined as follows :

- Difference between the CA125 levels after and before treatment (i.e., after minus before),
- Ratio of the CA125 levels after and before treatment (i.e, after divided by before).

```
after  = as.numeric(OTTA$ca125_posttx)
before = as.numeric(OTTA$ca125_pretx)
```

```r
    # COMMENT: warnings correspond to NAs
otta$treatmentresponse_diff  = after-before
otta$treatmentresponse_ratio = after/before
```

The upper left plot of the following figure, displaying the percentage of missing CA125 measures before, after, and before or after treatment, shows a high percentage of missing for the variable *Response to treatment* (80% of the sites delivered no CA125 measures). The remaining plots respectively display the boxplot of the *Response to treatment* on different scales. The most interesting one is the bottom right plot displaying the log of the ratio of the differences, showing a symmetrical distribution and a strong CA125 decrease for most participants (with an average CA125 level after treatment roughly equal to 23% of the CA125 level before treatment).

```r
par(mfrow=c(2,2),mar=c(2.5,2,3,1))
barplot(c(mean(is.na(before)),mean(is.na(after)),
          mean(is.na(otta$treatmentresponse_diff)))*100,col="light gray",
          ylim=c(0,100),
        names=c("Before","After","Before\nor After"),
        ylab="% missing data",main="% missing data"
        )
boxplot(otta$treatmentresponse_diff,col="light gray",main="After - Before")
abline(h=0,col="blue")
boxplot(I(otta$treatmentresponse_ratio),col="light gray",main="After / Before")
boxplot(log(otta$treatmentresponse_ratio),col="light gray",main="log(After / Before)")
abline(h=0,col="blue")
```

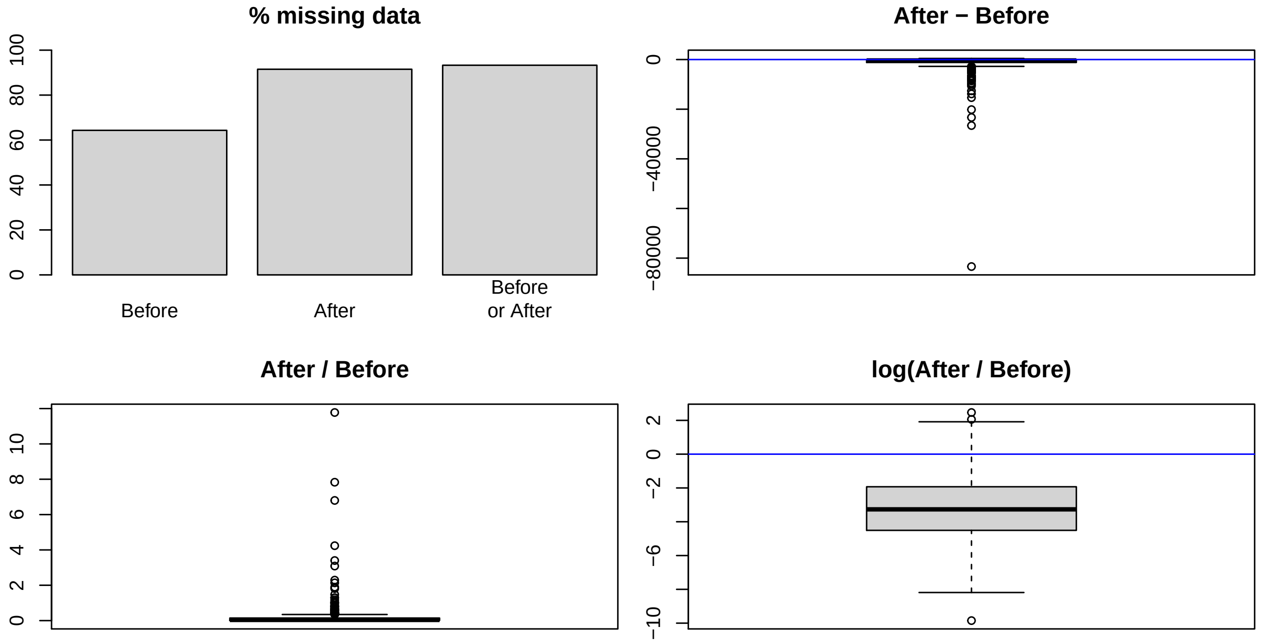

1.2.9 BRCA1 and BRCA2 mutation type

The levels of the variable *bra1_2* of the **OTTA** dataset are the following ones:

- 1 = pathogenic BRCA1 mutation,
- 2 = pathogenic BRCA2 mutation,
- 3 = pathogenic BRCA mutation NOS,

- 4 = tested wildtype (by any sort of testing),
- 6 = tested wildtype (full sequencing, and MLPA, of both genes),
- 7 = unclassified variant in BRCA1,
- 8 = unclassified variant in BRCA2,
- 9 = Unknown/Untested.

Due of the low number of observations per levels of the variable *bra1_2* of the **OTTA** dataset, it was decided to dichotomise the information into two categories, *Pathogenic* and *Other*, as shown in the following table

```
otta$mutation_type = factor(c(1,1,1,0,0,0,0,0,NA)[OTTA$brca1_2],
                            levels=c(1,0),labels=c("Pathogenic","Other"))
table(OTTA$brca1_2,otta$mutation_type,useNA="always")
```

```
##
##        Pathogenic Other <NA>
##   1           170     0    0
##   2            91     0    0
##   4             0  1626    0
##   6             0   106    0
##   7             0    42    0
##   8             0    60    0
##   9             0     0 1235
##   <NA>          0     0 2878
```

1.2.10 Presence of tumour after treatment

The levels of the variable *resdx* of the **OTTA** dataset, describing the size of the remaining disease when some tumour remained after surgery, are the following ones:

- 1 = no macroscopic disease,
- 2 = macroscopic disease 1 cm,
- 3 = macroscopic disease >1 and 2cm,
- 4 = macroscopic disease 2 cm,
- 5 = macroscopic disease >2 cm,
- 6 = macroscopic disease, size unknown,
- 7 = tumour not resected (eg inoperable; biopsy only),
- 9 = unknown.

Due of the low number of observations per levels of the variable *resdx* it was decided to dichotomise the information into two categories, *No disease left* versus *Some disease left*, as shown in the following table (The levels *8* and *888*, not described in the documentation, were considered as missing data).

```
temp = OTTA$resdx
temp[temp>=7] = NA
otta$tumourafter = factor(as.numeric(temp==1),levels=0:1,
                          labels=c("Some disease left","No disease left"))
table(OTTA$resdx,otta$tumourafter,useNA="always")
```

```
##
##        Some disease left No disease left <NA>
##   1                    0            2132    0
##   2                  482               0    0
##   3                   48               0    0
##   4                  111               0    0
##   5                  262               0    0
```

```
##    6                      627              0    0
##    7                        0              0   30
##    8                        0              0   52
##    9                        0              0  472
##    888                      0              0  216
##    <NA>                     0              0 1776
```

The following plot shows the log of the ratio between the CA125 levels post and ante surgery per level of the variable *Presence of tumour after treatment*. It may be noted that, on average, the CA125 level decreased more for patients with some tumour left than for patients without macroscopic disease after surgery.

```r
par(mfrow=c(1,1),mar=c(3,5,3,1))
boxplot(log(otta$treatmentresponse_ratio)~tumourafter,data=otta,
    ylab="log(CA125 After divided by CA125 Before)",
    col="light gray",axes=FALSE
    )
box()
axis(2)
axis(1,1:2,c("Some tumour\nleft","No tumour\nleft"),padj=0.5)
abline(h=0,col="blue")
```

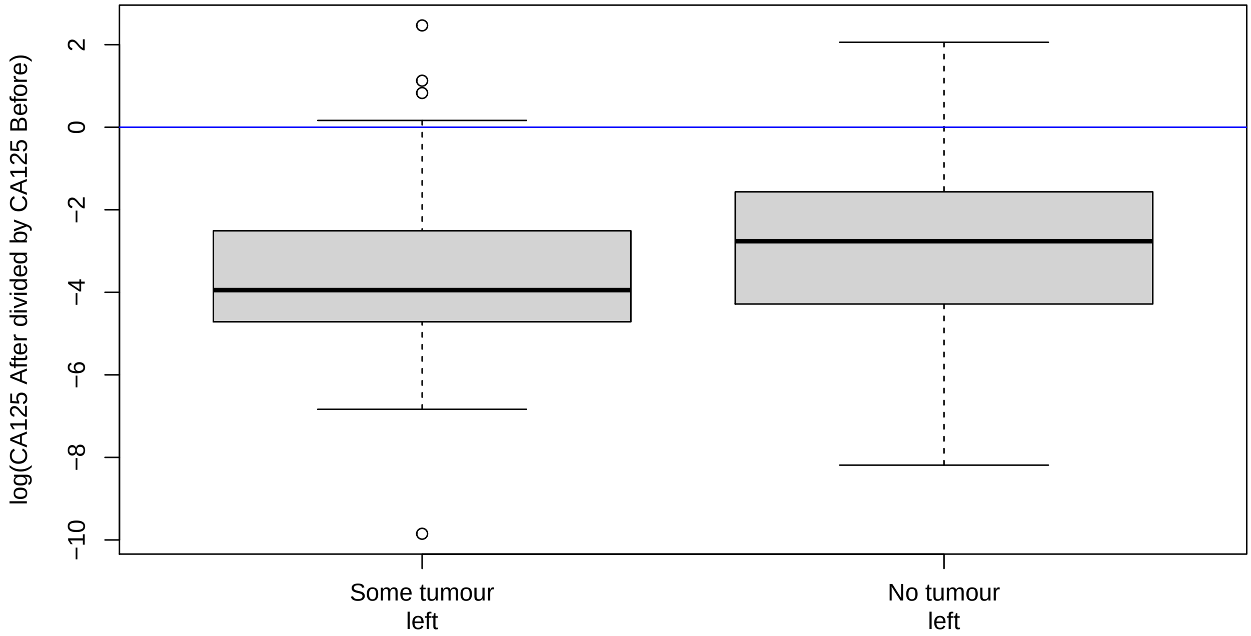

1.2.11 Presence of cancer within family

Several variables of the **OTTA** dataset measure the presence of breast, ovarian or unrelated cancer(s) in the family. The plot below shows the missing data pattern for these variables. Rows correspond to patients and columns to the variables of interest. Missings are coded in red. Most measures are missing with some clustering by rows due to sites.

```r
temp = OTTA[,c("brcancersis","ovcancersis","brcancerdau",
                "ovcancerdau","brcancermom","ovcancermom","brcancerdad")]
temp[temp==88|temp==77|temp==8] = NA
par(mfrow=c(1,1),mar=c(2.5,3,1,0))
image(is.na(t(temp[nrow(temp):1,])),col=c("light gray","red"),axes=FALSE)
```

```r
axis(2,c(1,0),c(1,nrow(temp)),las=2)
axis(1,seq(0,1,length=ncol(temp)),
     colnames(temp),cex.axis=.75)
```

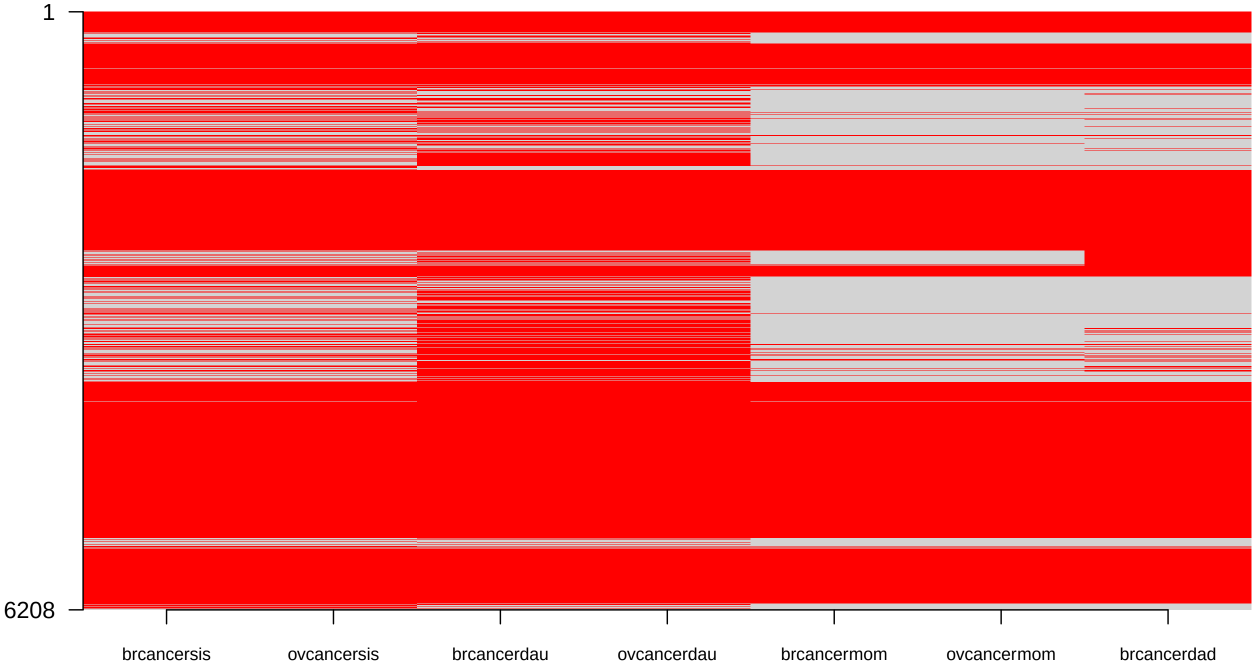

We chose to focus on the variable *Presence of related cancer for the mother* as the corresponding variables (*brcancermom*, *ovcancermom*, with coding 1 = Yes, 2 = No, 8 = Missing) show the lowest number of missing data, and as some sites seemed to have coded the information related to sisters and daughters differently.

```r
temp = OTTA[,c("brcancermom","ovcancermom")]
temp[temp==8] = NA
otta$cancer.mother = factor(as.numeric(apply(temp==1,1,any,na.rm=FALSE)),levels=c(0,1),
                            c("No related cancer of mother","Related cancer of mother"))
    # CHECK
    table(apply(temp[,c("brcancermom","ovcancermom")],1,paste,collapse="-"),
          otta$cancer.mother,useNA="always")
```

```
##
##          No related cancer of mother Related cancer of mother <NA>
##    1-1                             0                       12    0
##    1-2                             0                      188    0
##    2-1                             0                       48    0
##    2-2                          2028                        0    0
##    2-NA                            0                        0    1
##    NA-NA                           0                        0 3931
##    <NA>                            0                        0    0
```

1.2.12 Presence of prior cancers in the patient

The variables *prior1* to *prior4* of the **OTTA** dataset report the presence of related or unrelated cancer(s) in the patient with following levels:

- 0 = no prior primary cancer diagnosis,
- 1 = breast,
- 2 = colon,
- 3 = ovary,
- 4 = uterus,
- 5 = cervix,
- 6 = skin: nonmelanoma,
- 7 = skin: melanoma,
- 8 = lung,
- 9 =stomach,
- 10 = brain,
- 11 = other,
- 12 = skin: unknown if melanoma,

- 88 = Don't know if prior cancer.

The left plot of the next figure shows the missing data pattern for these variables, which is, again, site dependent. The right plot show the occurrence of each level for non-missing data. Note that *ovarian* cancer has no entry, confirming that the **OTTA** dataset focuses on primary cancer.

```
prior = OTTA[,c("prior1","prior2","prior3","prior4")]
prior[prior>80] = NA
par(mfrow=c(1,2),mar=c(7.5,3,1,1))
#
image(is.na(t(prior[nrow(prior):1,])),col=c("light gray","red"),axes=FALSE)
axis(2,c(1,0),c(1,nrow(prior)),las=2)
axis(1,seq(0,1,length=ncol(prior)),
     colnames(prior))
#
tablew = table(unlist(prior))
names(tablew) = c("No cancer","Breast","Colon","Uterus","Cervix","Skin (not m.)",
                  "Melanoma","Lung","Brain","Other","Skin (maybe m.)")
barplot(tablew,las=2)
```

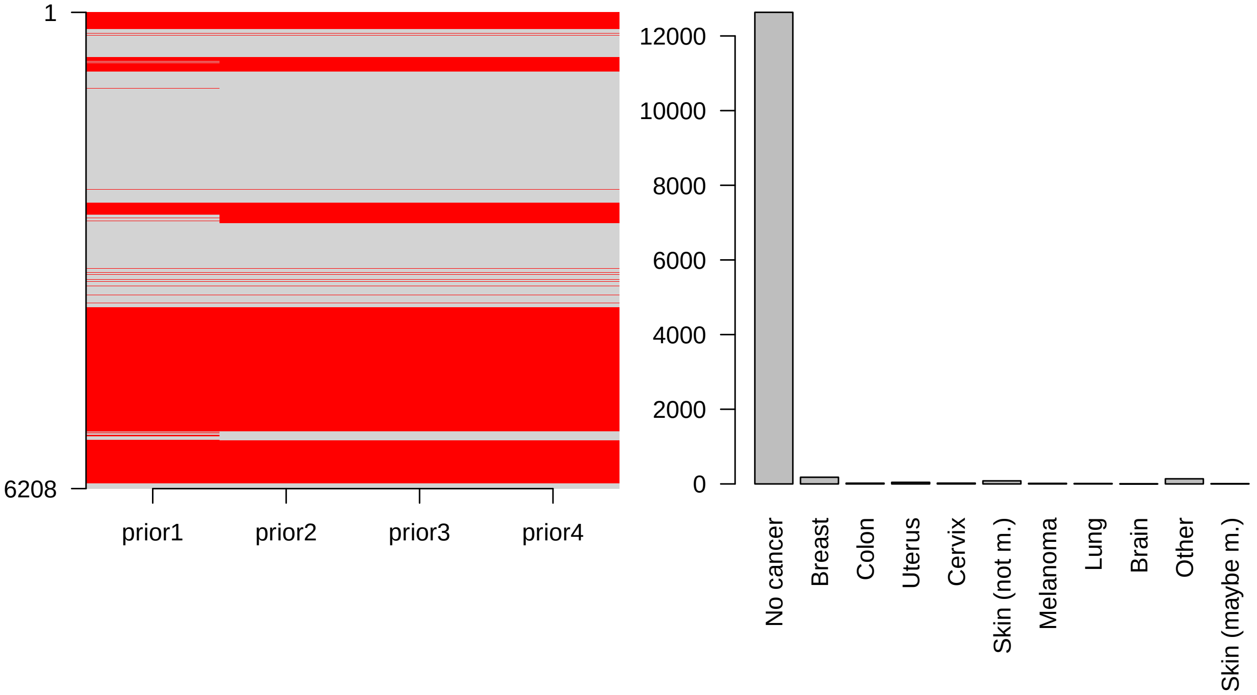

Due to the low number of occurrences per level, we chose to define the following two dichotomous variables :

- *Patient had previous related (i.e., 'breast') cancer*,
- *Patient had previous (i.e., 'any') cancer.*

The measures of patients with at least one missing in the *prior1* to *prior4* variables were set as missing. All measures of the site *s020* were set as missing as this site reported no previous cancer for any of its 69 patients, which was considered rather unlikely given the estimated probabilities of not having a previous cancer per site (ranging from 74% [s013] to 96% [s005]) even when assuming the most favourable scenario (i.e., the one of site s005).

```
otta$priorcancer = factor(as.numeric(apply(prior>0,1,any,na.rm=FALSE)),levels=c(0,1),
                          labels=c("Had no prior cancer","Had prior cancer"))
otta$priorrelatedcancer = factor(as.numeric(apply(prior==1,1,any,na.rm=FALSE)),
                                 levels=c(0,1),
                                 labels=c("Had no prior related cancer",
                                          "Had prior related cancer"))
table(otta$priorrelatedcancer,otta$priorcancer,useNA="always")
```

```
##
##                                Had no prior cancer Had prior cancer <NA>
##   Had no prior related cancer                 2682              281    0
##   Had prior related cancer                       0              176    0
##   <NA>                                           0                0 3069
```

1.2.13 Grade: tumour differentiation level

The variable *grade* of the **OTTA** dataset has the following levels

- 1 = well differentiated,
- 2 = moderately differentiated,
- 3 = poorly differentiated,

- 4 = undifferentiated,
- 6 = s013 unknown if grade 3 or 4,

- 7 = not applicable/LMP,
- 8 = DK.

The following plot shows the number of occurrences per level of the variable *grade*.

```
par(mfrow=c(1,1),mar=c(7.5,3,1,1))
grade = table(unlist(OTTA[,c("grade")]),useNA="always")
names(grade) = c("Well (1)","Moderate (2)","Poor (3)","None (4)","? (5)","Poor or None (6)","NA (7)","N
barplot(grade,las=2)
```

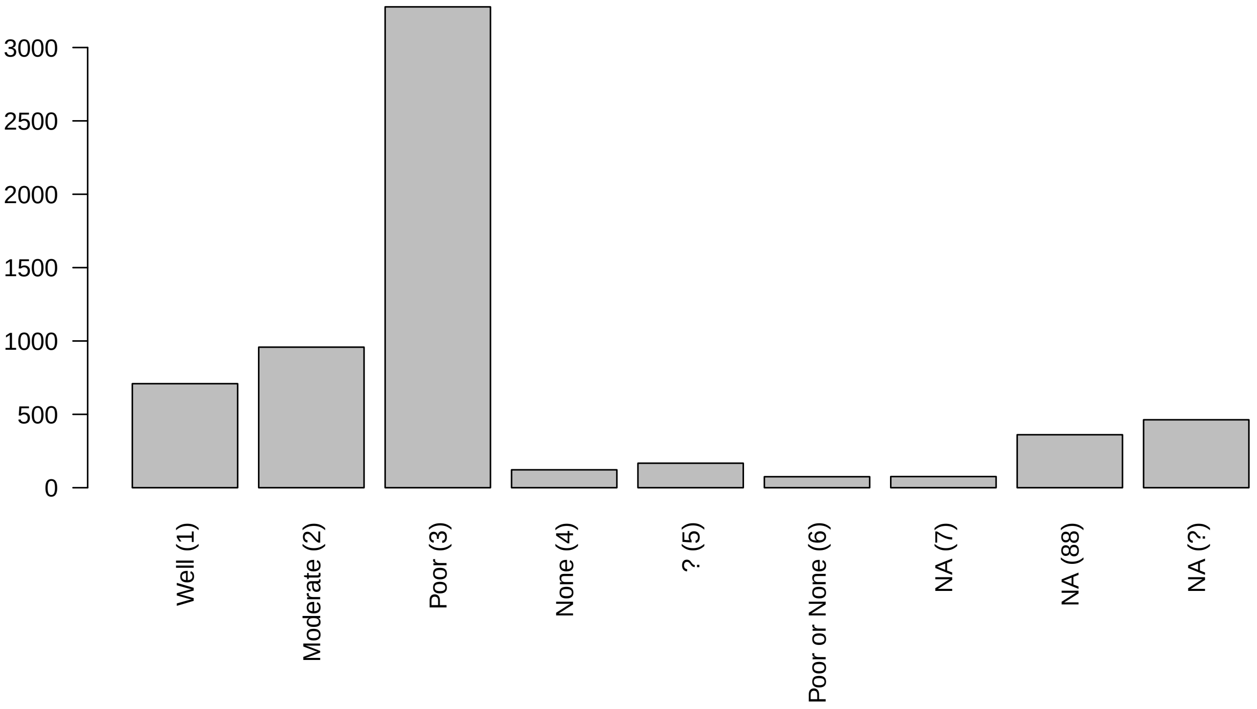

As the number of occurrences in the category *undifferentiated* was small and as the categories *poorly differentiated* and *undifferentiated* were undissociated for site s020, we chose to define the variable *Tumour differentiation level* by combining levels 3 and 4. Also, as the code 5 was not described, the 172 corresponding measures were coded as missing.

We defined the following 3- and 2-level factors:

```
otta$differentiation3 = factor(c(1,2,3,3,NA,3)[OTTA[,c("grade")] ],levels=1:3,
                               labels=c("Well","Moderate","Poor/No"))
otta$differentiation2 = factor(c(2,2,3,3,NA,3)[OTTA[,c("grade")] ],levels=2:3,
                               labels=c("Well/Moderate","Poor/No"))
table(otta$cancer_type2,otta$differentiation3,useNA="always")
```

```
##
##                 Well Moderate Poor/No <NA>
##   HGSOC            1      376    2575  292
##   Endometrioid   328      251     213   48
##   Clear cell      16       57     379  241
##   Mucinous       151      173      57   24
##   LGSOC          176        3      30    9
##   <NA>            37       98     220  453
```

```r
table(otta$cancer_type2,otta$differentiation2,useNA="always")
```

```
##
##                 Well/Moderate Poor/No <NA>
##   HGSOC                   377    2575  292
##   Endometrioid            579     213   48
##   Clear cell               73     379  241
##   Mucinous                324      57   24
##   LGSOC                   179      30    9
##   <NA>                    135     220  453
```

1.2.14 FIGO stating

The variable *figo* of the **OTTA** dataset has the following levels

- 1 = IA,
- 2 = IB,
- 3 = IC,
- 4 = I(NOS),
- 5 = IIA,
- 6 = IIB,
- 7 = IIC,
- 8 = II(NOS),
- 9 = IIIA,
- 10 = IIIB,
- 11 = IIIC,
- 12 = III(NOS),
- 13 = IV,
- 88 = DK

Due to the large number of levels of the 'figo' variable and to its small number of occurrences per level, we chose to combine the information in 4 stages as follows:

```r
otta$figo = factor(c(1,1,1,1,2,2,2,2,3,3,3,3,4)[OTTA[,c("figo")]],levels=1:4,
                   labels=c("Stage I","Stage II","Stage III","Stage IV"))
table(OTTA[,c("figo")] ,otta$figo,useNA="always")
```

```
##
##       Stage I Stage II Stage III Stage IV <NA>
##   1       656        0         0        0    0
##   2        57        0         0        0    0
##   3       582        0         0        0    0
##   4        97        0         0        0    0
##   5         0       73         0        0    0
##   6         0      199         0        0    0
##   7         0      413         0        0    0
##   8         0       49         0        0    0
##   9         0        0       133        0    0
##   10        0        0       284        0    0
##   11        0        0      2007        0    0
##   12        0        0       149        0    0
##   13        0        0         0      447    0
##   88        0        0         0        0  276
```

```
##   <NA>       0        0         0        0  786
```

```
table(otta$cancer_type2,otta$figo,useNA="always")
```

```
##
##                 Stage I Stage II Stage III Stage IV <NA>
##   HGSOC             236      284      1994      368  362
##   Endometrioid      342      189       122       14  173
##   Clear cell        295      154       130       12  102
##   Mucinous          217       36        60       11   81
##   LGSOC              45       18       104       12   39
##   <NA>              257       53       163       30  305
```

1.2.15 Age at diagnosis

The OTTA variable *refage revised* reports the age of patients at diagnosis. The left plot of the next figure shows the distribution of age at diagnosis per site (colour coded). Note that age at diagnosis seems site dependent (for site s012, for example, no patient was diagnosed after the age of 65). Also, in the site s019, 2 patients were diagnosed with ovarian cancer at the age 3 (outliers). The right plot shows number of occurrence per level of the corresponding 5-level ordinal variable we defined. 131 observations are missing.

```
otta$ageatdiagnosis = floor(OTTA[,c("refage_revised")])# floor recommended in dictionary
# 5-level ordinal factor
pt_group_age = rep(0,100)
pt_group_age[ 1: 39] = 1
pt_group_age[40: 49] = 2
pt_group_age[50: 59] = 3
pt_group_age[60: 69] = 4
pt_group_age[70:100] = 5
otta$agegroupatdiagnosis = factor(pt_group_age[otta$ageatdiagnosis],1:5,c("]0,40[ y.o.","[40,50[ y.o.",
# plots
par(mfrow=c(1,2),mar=c(7.5,3,1,1))
#
otta2 = otta[order(otta$site),]
plot(otta2$ageatdiagnosis,col=rainbow(nlevels(factor(otta2$site)))[as.numeric(otta2$site)],
     axes=FALSE,ylim=c(0,100),xlab="Participants and sites",ylab="Age")
abline(h=0)
axis(1,tapply(1:nrow(otta2),otta2$site,mean),levels(factor(otta2$site)),
     las=2,cex.axis=.5,pos=0)
axis(2,seq(0,100,10),las=2)
#
temp1 = table(otta$agegroupatdiagnosis,useNA="always")
names(temp1)[is.na(names(temp1))] = "NA"
barplot(temp1,las=2)
```

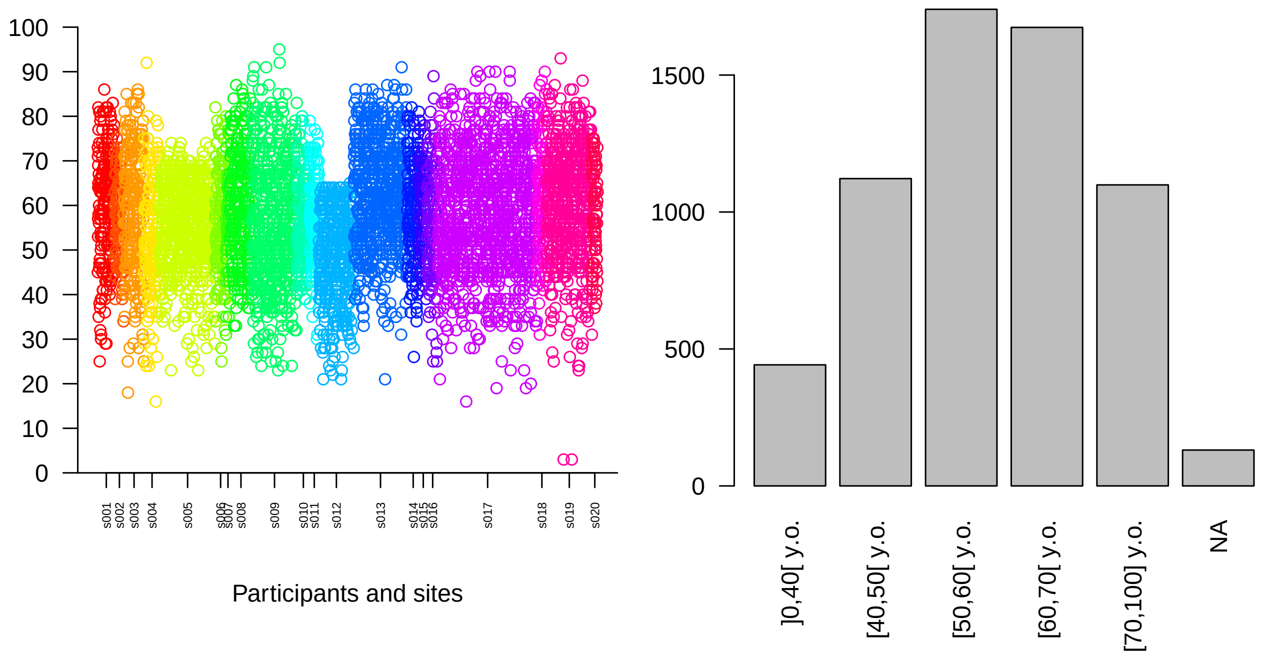

1.2.16 last follow up information

The variable *timelastfu* of the **OTTA** dataset reports the time of last follow up from day of diagnosis. The left plot of the next figure shows the distribution of the time of last follow up from day of diagnosis per site (colour coded). The maximum follow up time was over 30 years. For sites s007 and s005, the minimum time of last follow up can was greater than one year. The right plot shows the age at last follow up, obtained by adding the time of last follow up in years to the age at diagnosis.

```
otta$time.lastfollowup = OTTA[,c("timelastfu")]
sapply(split(otta$time.lastfollowup/365.25,otta$site),
       function(x)c(min=min(x,na.rm=TRUE),mean=mean(x,na.rm=TRUE),
                    max=max(x,na.rm=TRUE),na=sum(is.na(x))))
```

```
##          s001        s002        s003        s004      s005       s006
## min  0.000000  0.04928131  0.008213552  0.08213552  1.325120  0.4900753
## mean 3.324533  6.71868583  4.580131026  4.56159074  9.295624  8.7960509
## max  9.938398 20.60506502 21.253935661 15.44147844 31.058179 20.2354552
## na   3.000000  3.00000000  0.000000000 38.00000000 39.000000  0.0000000
##           s007        s008       s009      s010       s011        s012
## min   1.075975  0.02190281  0.0109514 0.6078029  0.3969884  0.03011636
## mean  6.031369  4.02337239  6.9400857 4.5072395  3.4957493  7.61019061
## max  10.182067 20.01095140 29.1362081 9.2867899 18.9568789 13.99315537
## na    4.000000  0.00000000  0.0000000 0.0000000  8.0000000  0.00000000
##             s013       s014        s015        s016         s017
## min   0.01368925 0.02190281  0.06844627  0.02737851  0.005475702
## mean  4.87081025 1.92374173  4.24355921  4.03295637  5.399382774
## max  14.79808350 4.95550992 11.58110883 19.67693361 23.561943874
## na    0.00000000 0.00000000  0.00000000  0.00000000 99.000000000
##            s018      s019       s020
## min   0.0164271  0.000000  0.03832991
## mean  4.8206766  4.591197  7.33843208
```

```
## max  12.4188912 18.316222 16.99657769
## na    0.0000000 14.000000  0.00000000
```

```r
# figure
par(mfrow=c(1,2),mar=c(2,3,3,1))
#
otta2 = otta[order(otta$site),]
plot(otta2$time.lastfollowup/365.25,
     col=rainbow(nlevels(factor(otta2$site)))[as.numeric(otta2$site)],axes=FALSE,
     main="Time of last follow up in years\nfrom day of diagnosis",
     xlab="Participants and sites",ylab="Years")
abline(h=0)
axis(1,tapply(1:nrow(otta2),otta2$site,mean),levels(factor(otta2$site)),
     las=2,cex.axis=.5,pos=0)
axis(2,seq(0,30,5),las=2)
#
plot(otta2$time.lastfollowup/365+otta2$ageatdiagnosis,
     col=rainbow(nlevels(factor(otta2$site)))[as.numeric(otta2$site)],axes=FALSE,
     main="Age at time of last follow up",
     xlab="Participants and sites",ylab="Years")
abline(h=0)
axis(1,tapply(1:nrow(otta2),otta2$site,mean),levels(factor(otta2$site)),
     las=2,cex.axis=.5,pos=0)
axis(2,seq(0,100,10),las=2)
```

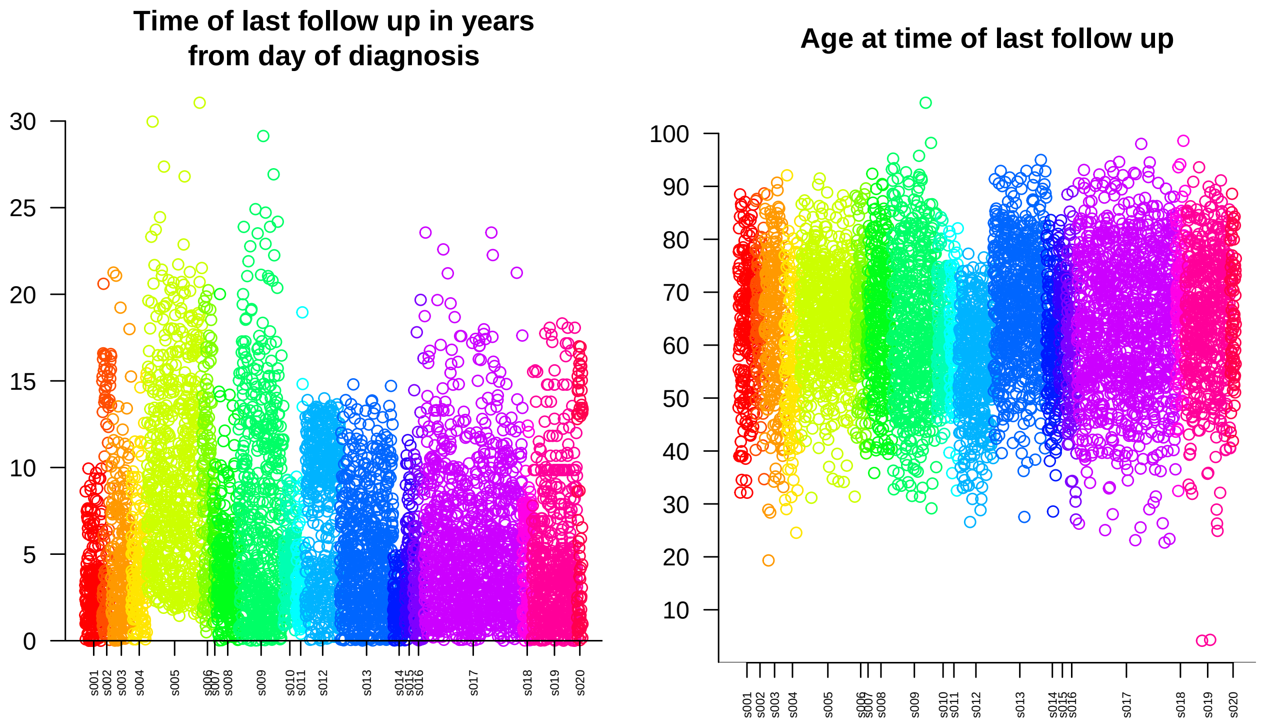

1.2.17 Status at last follow up

The OTTA variables *finalstatus* (with levels 1 = alive, 2 = dead, 8 = did not follow) and *causedeath* (with levels 1 = Progression of Disease - known or presumed, 2 = Treatment related, 3 = Other, 7 = Not applicable

(i.e. still alive), 9 = Unknown) respectively report the status at time of last follow up and the cause of death.

The following table shows the number of occurrences per combination of both variables. Note that observations with code 8 (undocumented) for variable *finalstatus* and with codes 0 (undocumented) and 9 for variable *causedeath* were imported as missing.

```
deadalive.lastfollowup = factor(c(0,1)[OTTA[,c("finalstatus")] ],levels=0:1,
                                labels=c("alive","dead"))
cause.death = factor(OTTA[,c("causedeath")] ,levels=c(1,2,3,7),
                     labels = c("disease","treatment","no related","alive"))
table(cause.death,deadalive.lastfollowup ,useNA="always")
```

```
##              deadalive.lastfollowup
## cause.death  alive dead <NA>
##   disease        1 2111    1
##   treatment      0   26    0
##   no related     0  227    0
##   alive       1690   40    0
##   <NA>         934  974  204
```

For this table, we chose to define the variable *Status at last follow up* with levels *Alive*,*Dead (disease)*, *Dead (treatment)*, *Dead (other)*, *Dead (unknown)* and NA, according to the following rules :

- *Alive* if finalstatus = *Alive* (even when coded NAs in causedeath),
- *Dead (unknown)* if finalstatus = *Dead* AND causedeath == NA,
- *Dead (disease)* if finalstatus = *Dead* AND causedeath == *Disease*,
- *Dead (treatment)* if finalstatus = *Dead* AND cause.death == *Treatment*.
- *Dead (not related)* if finalstatus = *Dead* AND cause.death == *Not related*. s Also, we chose to address the inconsistencies of the last table in the following way (Note that these choices may play a role in the survival analyses presented in Section 4) :
- 1 participant has *Alive* as status but *Disease* as cause of death. This participant was coded as missing.
- 1 participant has NA status but *Disease* as cause of death. This participant was coded as missing.
- 40 participant are *Alive* according to cause of death but *Dead* according to their status at last follow up. These participants were coded as missing.
- 986 participants are *Alive* according to their status at last follow up but NAs according to the reported cause of death. These participants were coded as *Alive*.
- 993 participants are dead according to their status at last follow but NAs according to the reported cause of death. These participants were coded as *Dead (unknown)*.

```
status.lastfollowup = rep(NA,nrow(otta))
# 1)
status.lastfollowup[deadalive.lastfollowup=="alive"] = "Alive"
# 2)
status.lastfollowup[deadalive.lastfollowup=="dead"&is.na(cause.death)] = "Dead (unknown)"
# 3)
status.lastfollowup[deadalive.lastfollowup=="dead"&cause.death=="disease"] = "Dead (disease)"
# 4)
status.lastfollowup[deadalive.lastfollowup=="dead"&cause.death=="treatment"] = "Dead (treatment)"
# 5)
status.lastfollowup[deadalive.lastfollowup=="dead"&cause.death=="no related"] = "Dead (other)"
# 6)
status.lastfollowup[deadalive.lastfollowup=="alive"&cause.death=="disease"] = NA
```

```r
# finalisation
otta$status.lastfollowup = factor(status.lastfollowup,
                                  levels=c("Alive","Dead (disease)","Dead (treatment)",
                                           "Dead (other)","Dead (unknown)"))
```

1.2.18 Time of interview

The OTTA variables *timeint_revised* reports the time of interview from the day of diagnotic. The next figure shows the following information

- The left upper plot of the next figure shows the distribution of the time of interview for each patient and site (colour coded),
- The right upper plot shows the percentage of patients immediately included in the **OTTA** databank (times of interview equal to 0) per site,
- The left lower plot shows the average time to interview (in years) per set in years,
- The right lower plot shows the the time to interview (y-axis) versus the time of last follow-up (x-axis).

```r
otta$time.interview = OTTA[,"timeint_revised"]
# summary stat
mean_site = tapply(otta$time.interview/365.25,otta$site,mean,na.rm=TRUE)
pi0_site  = tapply((otta$time.interview/365.25)==0,otta$site,mean,na.rm=TRUE)
# figure
par(mfrow=c(2,2),mar=c(4,4,3,1))
#
otta2 = otta[order(otta$site),]
plot(otta2$time.interview/365.25,
     col=rainbow(nlevels(factor(otta2$site)))[as.numeric(otta2$site)],axes=FALSE,
     main="Time of interview in years\nfrom day of diagnosis",
     xlab="Participants and sites",ylab="Years")
abline(h=0)
axis(1,tapply(1:nrow(otta2),otta2$site,mean),levels(factor(otta2$site)),
     las=2,cex.axis=.5,pos=0)
axis(2,seq(0,30,5),las=2)
#
barplot(pi0_site*100,col=rainbow(nlevels(factor(otta2$site))),
        xlab="Sites",ylab="Percentage",
        main = "Percentage of time to interview\n equal to 0",
        cex.names=.5,las=2)
#
barplot(mean_site,col=rainbow(nlevels(factor(otta2$site))),
        xlab="Sites",ylab="Years",
        main = "Average time to interview",
        cex.names=.5,las=2)
#
plot(otta$time.lastfollowup/365.25,otta$time.interview/365.25,
     col=rainbow(nlevels(factor(otta2$site)))[as.numeric(otta2$site)],axes=FALSE,
     main="Time of interview versus\nTime of last follow up",
     xlab="Time of last follow up (in years)",
     ylab="time of interview (in years)")
abline(0,1)
abline(-13.5,1,lty=2)
```

```r
abline(h=0)
axis(2,seq(0,30,5),las=2)
axis(1,seq(0,30,5),las=1)
```

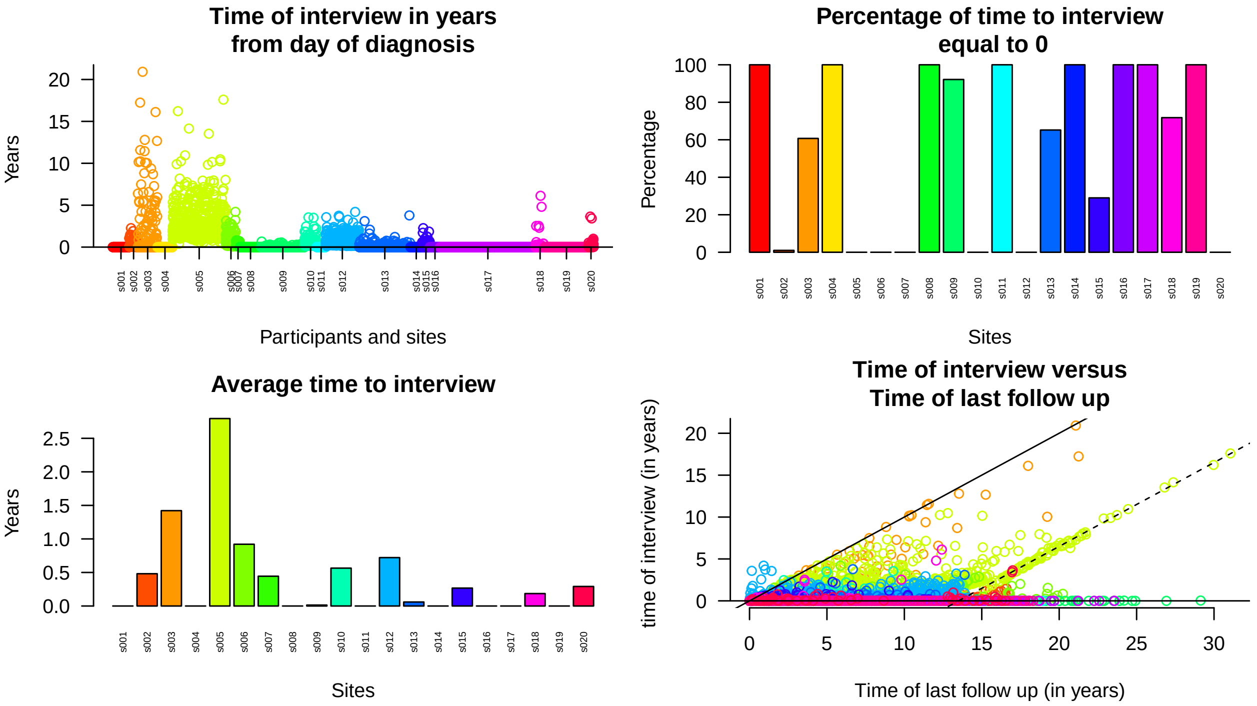

The 3 first plots show that time of interview is site dependent, with some sites immediately including patients to the OTTA in all (s016, s011, s004, s008, s019, s014, s001 and s017) or most cases (s009, s018), while other always include them with a relatively large delays (2.75 years on average for s005). Some interviews came 10 years after diagnosis.

The comparison of the time of interview and time of last follow-up shows

- that the time of interview may be larger than the time of last follow-up. This may correspond to sites who interviewed patients before they got diagnosed (theoretically, they were set to 0 according to the dictionary) or to errors. For such cases, we chose to set *time of interview* to NA.

- that some sites seem to organise data collection in waves (time of last follow-up often comes 13.5 years after time of interview for site s005),
- that the data collection occured at the time of last follow-up (time of interview equals time of last follow-up) in some cases.

```r
posw = which(otta$time.interview>otta$time.lastfollowup &
             !is.na(otta$time.interview>otta$time.lastfollowup))
table(otta$site[posw])
```

```
##
## s001 s002 s003 s004 s005 s006 s007 s008 s009 s010 s011 s012 s013 s014 s015
##    0    0    0    0    0    0    0    0    0    1    0   15    0    0    0
## s016 s017 s018 s019 s020
##    0    0    0    0    1
```

```r
otta$time.interview[posw] = NA
```

1.2.19 Save datsets

```
# select rows
otta = otta[!is.na(otta$cancer_type),]
otta = otta[order(otta$site),]
# size
n = nrow(otta)
p = ncol(otta)
```

The **otta** dataset only considers patients with one of the five cancer types of interest. It has 5400 rows (patients) and 33 columns.

Both the **OTTA** (raw combined dataset) and **otta** (amended dataset) data frames are saved under results/0-data/ as rdata and csv files.

```
# rdata files
save(otta,file=paste0("results/rdata/1-otta-amended.rd"))
save(OTTA,file=paste0("results/rdata/1-otta-raw.rd"))

# csv files
write.csv(otta,file=paste0("results/tables/1-otta-amended.csv"))
write.csv(OTTA,file=paste0("results/tables/1-otta-raw.csv"))
```

1.3 Descriptive analyses

The imported **otta** dataset has 5400 rows (patients) and 33 columns. In the next sections, we propose

- descriptive analysis of the missing data pattern,
- patient and patient-year sample sizes per cancer type and outcome of interest
- summary statistics for some time-related and continuous variables.

1.3.1 Missing data

The next plot (**Figure S1 of the article**) shows the missing data pattern in the imported **otta** dataset for each variables (y-axis) and patients ordered by sites (x-axis). Non-missing data appear in grey. Missing data are colour coded by site. The percentage of non-missing data per variable is indicated.

```
otta2 = is.na(otta)
otta2[is.na(otta)] = rep(as.numeric(otta$site),ncol(otta))[is.na(otta)]
par(mfrow=c(1,1),mar=c(2,4,4,8))
#
image(otta2[1:nrow(otta2),ncol(otta2):1],
      col=c("light gray",rainbow(nlevels(otta$site))),
      main="Global missing data",axes=FALSE)
axis(3,c(0,1),c(1,nrow(otta)),las=1)
axis(1,tapply(1:nrow(otta),otta$site,mean)/nrow(otta),levels(factor(otta$site)),
     las=2,cex.axis=.5)
axis(4,seq(0,1,length=ncol(otta)),
     colnames(otta)[ncol(otta):1],las=2,cex.axis=.75)
axis(2,seq(0,1,length=ncol(otta)),
     paste0(format(round(apply(!is.na(otta),2,mean)*100,2)),"%")[ncol(otta):1],las=2,cex.axis=.75)
```

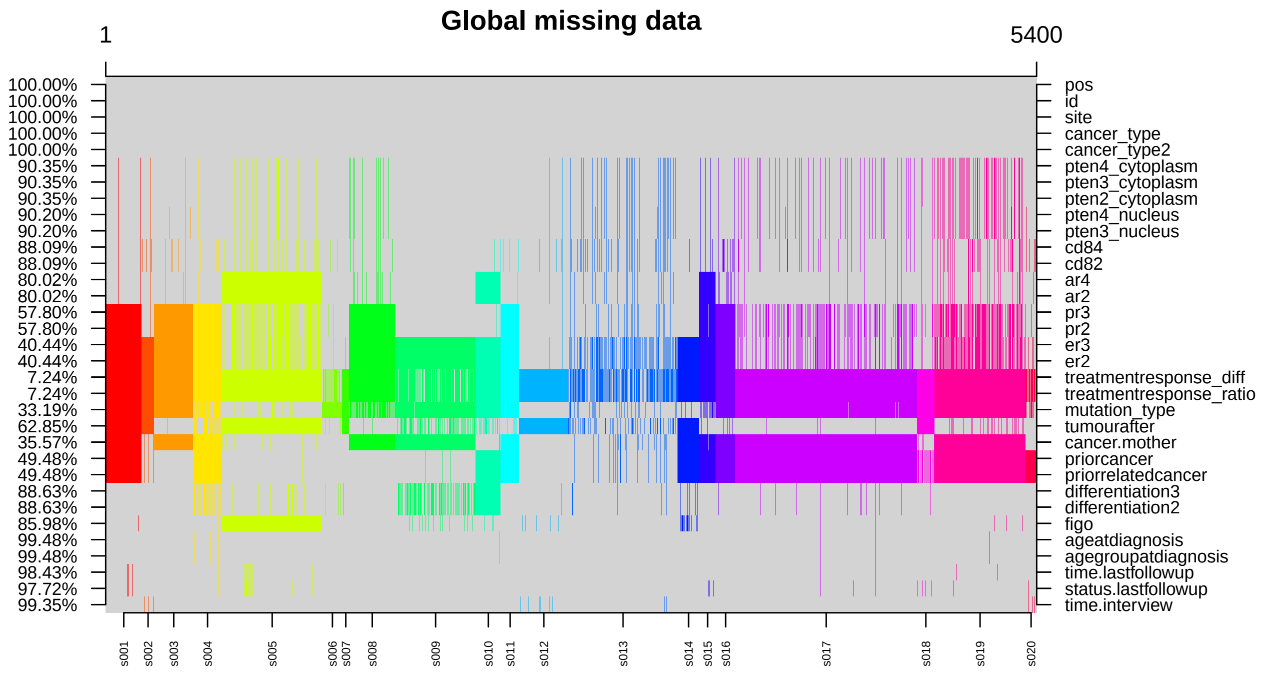

We can note that

- the missing data pattern is site dependent. Some sites have no/few AR (s015, s005, s010), PR (s003, s016, s011, s004, s008, s001), ER (s015, s009, s003, s016, s011, s004, s002, s008, s014) or FIGO measures (s005),
- some variables show a high percentage of missingnesss like *treatment response* (~92.5%), *mutation type* (~67%), presence of cancer in the mother (~65%) or in the patient (~50%).

1.3.2 Sample size

In this section, we define the sample size per cancer type and level of the variables *PTEN*, *CD8*, *AR*, *PR*, *ER Presence of tumour after surgery*, *Differentiation level*, *Age group at diagnosis* and *site* in term of

- number of participants (**lower part of the Table 1 of the article**, saved as results/1-import/otta-samplesize-participants.csv),
- number of person-year (**Table S1 of the article**, saved under results/1-import/otta-samplesize-personyears.csv).

1.3.2.1 Number of participants

```
# create table
Table1 = NULL
for(ow in 1:n.outcome){
    ## participant sample size
    table = table(otta[,id.outcome$id[ow]],otta$cancer_type2)
    table = rbind(apply(table,2,sum),table)
    table = cbind(table,apply(table,1,sum))
    rownames(table)[1] = "Total"
    colnames(table)[ncol(table)] = "Total"
    # title, percentage par level within cancer type and save
```

```
    table = cbind(Variable=NA,Level=rownames(table),
                  apply(table,2,function(x) paste0(x," (",format(round(x/sum(x[-1])*100,0)),")")))
    table[,1] = c(id.outcome$name[ow],rep("",nrow(table)-1))
    Table1 = rbind(Table1,table)
    }
# add total
table = matrix(c("OTTA dataset","Total",
                 paste0(c(table(otta$cancer_type2),nrow(otta))," (100)")),
               ncol=nlevels(otta$cancer_type2)+3,
               dimnames=list(NULL,c("Variable","Level",levels(otta$cancer_type2),"Total")))
Table1 = rbind(table,Table1)
# print
data.frame(Table1)
```

```
# save table as csv file
write.csv(Table1,file=paste0("results/tables/1-otta-samplesize-participants.csv"))
```

1.3.2.2 Number of person-years

```
# define number of days per patient
day.i = round(otta$time.lastfollowup)
day.i[is.na(day.i)] = 0 # missing dataset to 0


# create table
Table2 = NULL
for(ow in 1:n.outcome){
    ## person-year sample size
    table = round(table(rep(otta[,id.outcome$id[ow]],day.i),
                        rep(otta$cancer_type2,day.i))/365.25)
    table = rbind(apply(table,2,sum),table)
    table = cbind(table,apply(table,1,sum))
    rownames(table)[1] = "Total"
    colnames(table)[ncol(table)] = "Total"
    # title, percentage par level within cancer type and save
    table = cbind(Variable=NA,Level=rownames(table),
                  apply(table,2,function(x) paste0(x," (",format(round(x/sum(x[-1])*100,0)),")")))
    table[,1] = c(id.outcome$name[ow],rep("",nrow(table)-1))
    Table2 = rbind(Table2,table)
    }
# add total
table = matrix(c("OTTA dataset","Total",
                 paste0(round(c(table(rep(otta$cancer_type2,day.i)),
                        sum(day.i))/365.25)," (100)")),
               ncol=nlevels(otta$cancer_type2)+3,
               dimnames=list(NULL,c("Variable","Level",levels(otta$cancer_type2),"Total")))
Table2 = rbind(table,Table2)
# print
data.frame(Table2)
```

```
# save table as csv file
write.csv(Table2,file=paste0("results/tables/1-otta-samplesize-personyears.csv"))
```

1.3.3 Summary statistics

In this section, we define a few summary statistics for the variables *Age at diagnosis*, *Number of follow-up years* and *Status at follow up*. The corresponding table (**upper part of Table 1 of the article**) is saved under saved as results/1-import/otta-summarystats.csv.

```
Table3 = NULL
# age at diagnosis:
mean.1 = c(tapply(otta$ageatdiagnosis,otta$cancer_type2,mean,na.rm=TRUE),
           Total=mean(otta$ageatdiagnosis,na.rm=TRUE))
sd.1   = sqrt(c(tapply(otta$ageatdiagnosis,otta$cancer_type2,var,na.rm=TRUE),
                Total=var(otta$ageatdiagnosis,na.rm=TRUE)))
comb.1 = cbind(NA,c("Mean","SD"),round(rbind(mean.1,sd.1),1))
comb.1[,1] = c("Age at diagnosis",rep("",nrow(comb.1)-1))
Table3 = rbind(Table3,comb.1)
# number of follow-up years:
mean.2 = c(tapply(otta$time.lastfollowup/365.25,otta$cancer_type2,mean,na.rm=TRUE),
           Total=mean(otta$time.lastfollowup/365.25,na.rm=TRUE))
sd.2   = sqrt(c(tapply(otta$time.lastfollowup/365.25,otta$cancer_type2,var,na.rm=TRUE),
                Total=var(otta$time.lastfollowup/365.25,na.rm=TRUE)))
comb.2 = cbind(NA,c("Mean","SD"),round(rbind(mean.2,sd.2),1))
comb.2[,1] = c("Years followed",rep("",nrow(comb.2)-1))
Table3 = rbind(Table3,comb.2)
# number of follow-up years:
pi.3   = c(tapply(otta$time.interview/365.25>0,otta$cancer_type2,mean,na.rm=TRUE),
             Total=mean(otta$time.interview/365.25>0,na.rm=TRUE))
mean.3a   = c(tapply(otta$time.interview/365.25,
                    otta$cancer_type2,mean,na.rm=TRUE),
             Total=mean(otta$time.interview/365.25,na.rm=TRUE))
mean.3b   = c(tapply(otta$time.interview[otta$time.interview>0]/365.25,
                    otta$cancer_type2[otta$time.interview>0],mean,na.rm=TRUE),
             Total=mean(otta$time.interview[otta$time.interview>0]/365.25,na.rm=TRUE))
sd.3      = sqrt(c(tapply(otta$time.interview[otta$time.interview>0]/365.25,
                         otta$cancer_type2[otta$time.interview>0],var,na.rm=TRUE),
                  Total=var(otta$time.interview[otta$time.interview>0]/365.25,na.rm=TRUE)))
comb.3 = cbind(NA,c("Mean","% > 0","Mean | > 0","SD | > 0"),
               round(rbind(mean.3a,pi.3*100,mean.3b,sd.3),1))
comb.3[,1] = c("Delayed entry",rep("",nrow(comb.3)-1))
Table3 = rbind(Table3,comb.3)
rownames(Table3) = 1:nrow(Table3)
# print
as.data.frame(Table3)
```

```
# save table as csv file
write.csv(Table3,file=paste0("results/tables/1-otta-summarystats.csv"),
          row.names = FALSE)
```

2 Two-Rater PTEN scores dataset

For a subset of OTTA participants, cytoplasmic and nucleus PTEN scoring were defined by two raters. The following code creates the dataset **id.core** indicating, for each core (rows), the participant id and the PTEN ratings of both raters (columns).

```
id.core = read.csv("data/core_noid.csv",header=TRUE,stringsAsFactors=FALSE)
rownames(id.core) = id.core$id
n.core = nrow(id.core)
head(id.core)
```

The dataset, saved under results/0-data/raters.rd, shows the PTEN scores of 2 raters for 678 cores of 404 **OTTA** patients.

Script 2 - Agreement

D.-L. Couturier / F. Martins / J. Brenton / P. Pharoah

Last modified: 13 Mar 2020

```
#   Input data files  :    results/rdata/raters.rd
#   Output data files :    results/rdata/boot.rd
#   Required R packages :  colorspace, vcd, psych
```

1 Define scores per raters

Here we deduce the cytoplasmic and nucleus PTEN scores from the raw **w.x.yz** scores for both raters.

```
load("results/rdata/1-raters.rd")
wxyz_rater1 = strsplit(id.core$raw_rater1, "[.]")
wxyz_rater2 = strsplit(id.core$raw_rater2, "[.]")

# cytoplasmic scores
id.core$cytoplasm_rater1 = as.numeric(unlist(lapply(wxyz_rater1,function(x)x[[1]])))
id.core$cytoplasm_rater2 = as.numeric(unlist(lapply(wxyz_rater2,function(x)x[[1]])))
    # COMMENT: warnings correspond to NAs

# nucleus scores
id.core$nucleus_rater1 = as.numeric(unlist(lapply(wxyz_rater1,function(x)x[[2]])))
id.core$nucleus_rater2 = as.numeric(unlist(lapply(wxyz_rater2,function(x)x[[2]])))
    # COMMENT: warnings correspond to NAs
```

2 Descriptive analyses

The left plot of the next figure (**Table S2 of the article**) compares the cytoplasmic PTEN scores of both raters. Cases would all lie on the diagonal in cases of perfect agreement. The right plot performs the same comparison for nucleus PTEN scores.

```
par(mar=c(3,3,8,7))
layout(matrix(c(1,2,3), nrow=1, ncol=3), widths=c(2,2,.75), heights=c(1))
colw    = col2.fun(200)[200:1]
colw[1] = "white"

## cytoplasm
table_cytoplasm = table(rater1=id.core$cytoplasm_rater1,
                        rater2=id.core$cytoplasm_rater2)
image(1:nrow(table_cytoplasm),1:nrow(table_cytoplasm),t(table_cytoplasm[nrow(table_cytoplasm):1,]),zlim=
      xlab="",ylab="",main="")
axis(3,at=1:5,c("0-Negative","1-Weak","2-Normal","3-Heterogenous","9-Not Assessable"),las=2,cex.axis=.7
axis(4,at=5:1,c("0-Negative","1-Weak","2-Normal","3-Heterogenous","9-Not Assessable"),las=2,cex.axis=.7
axis(2,at=3,c("First rater"),cex.axis=1.25,tick=FALSE)
```

```
axis(1,at=3,c("Second rater"),cex.axis=1.25,tick=FALSE)
axis(3,at=3,c("Cytoplasmic PTEN measures"),cex.axis=1.5,tick=FALSE,padj=-6)
box(col="light gray")
    #for(i in 1:5){rect(i-.5,(5:1)[i]-.5,i+.5,(5:1)[i]+.5,col=NA,border=gray(.5),lwd=2)}
abline(h=seq(0,5,1)-.5,col=gray(.9))
abline(v=seq(0,5,1)-.5,col=gray(.9))
# add numbers
for(i in 1:nrow(table_cytoplasm)){
    for(j in 1:ncol(table_cytoplasm)){
        text(i,(ncol(table_cytoplasm):1)[j],t(table_cytoplasm)[i,j])
    }
}

## following suggestions of BJC reviewer 3:

# 2 tier
table_cytoplasm_tier2 = table(rater1=c(1,1,2,1)[id.core$cytoplasm_rater1+1],
                              rater2=c(1,1,2,1)[id.core$cytoplasm_rater2+1])
psych::cohen.kappa(table_cytoplasm_tier2)$kappa
```

```
## [1] 0.4628716
```

```
table_cytoplasm_tier2 = table(rater1=c(1,1,2,NA)[id.core$cytoplasm_rater1+1],
                              rater2=c(1,1,2,NA)[id.core$cytoplasm_rater2+1])
psych::cohen.kappa(table_cytoplasm_tier2)$kappa
```

```
## [1] 0.5220202
```

```
table_cytoplasm_tier2 = table(rater1=c(1,2,2,NA)[id.core$cytoplasm_rater1+1],
                              rater2=c(1,2,2,NA)[id.core$cytoplasm_rater2+1])
psych::cohen.kappa(table_cytoplasm_tier2)$kappa
```

```
## [1] 0.4116456
```

```
# 3 tier
table_cytoplasm_tier3 = table(rater1=c(0,1,1,2)[id.core$cytoplasm_rater1+1],
                              rater2=c(0,1,1,2)[id.core$cytoplasm_rater2+1])
psych::cohen.kappa(table_cytoplasm_tier2)$kappa
```

```
## [1] 0.4116456
```

```
## nucleus
table_nucleus = table(rater1=id.core$nucleus_rater1,
                      rater2=id.core$nucleus_rater2)
image(1:nrow(table_nucleus),1:nrow(table_nucleus),t(table_nucleus[nrow(table_nucleus):1,]),zlim=c(0,200)
      xlab="",ylab="",main="")
axis(3,at=1:5,c("0%","<10%","10 to 50%",">50/%","Not Assessable"),las=2,cex.axis=.7,tick=FALSE)
axis(4,at=5:1,c("0%","<10%","10 to 50%",">50/%","Not Assessable"),las=2,cex.axis=.7,tick=FALSE)
axis(2,at=3,c("First rater"),cex.axis=1.25,tick=FALSE)
axis(1,at=3,c("Second rater"),cex.axis=1.25,tick=FALSE)
axis(3,at=3,c("Nucleus PTEN measures"),cex.axis=1.5,tick=FALSE,padj=-6)
box(col="light gray")
    #for(i in 1:5){rect(i-.5,(5:1)[i]-.5,i+.5,(5:1)[i]+.5,col=NA,border=gray(.5),lwd=2)}
abline(h=seq(0,5,1)-.5,col=gray(.9))
abline(v=seq(0,5,1)-.5,col=gray(.9))
#
```

```
# add numbers
for(i in 1:nrow(table_nucleus)){
    for(j in 1:ncol(table_nucleus)){
        text(i,(ncol(table_nucleus):1)[j],t(table_nucleus)[i,j])
    }
}

## legend
temp = barplot(rep(1,200),plot=FALSE)
barplot(rep(1,200),col=colw,horiz = TRUE,border=colw,axes=FALSE)
rect(0,0,1,max(temp)+1,col=NA,border="light gray")
posw = c(1,seq(25,200,25))
axis(4,pos=1,at=temp[posw],seq(0,200,25),las=2,cex.axis=.75)
```

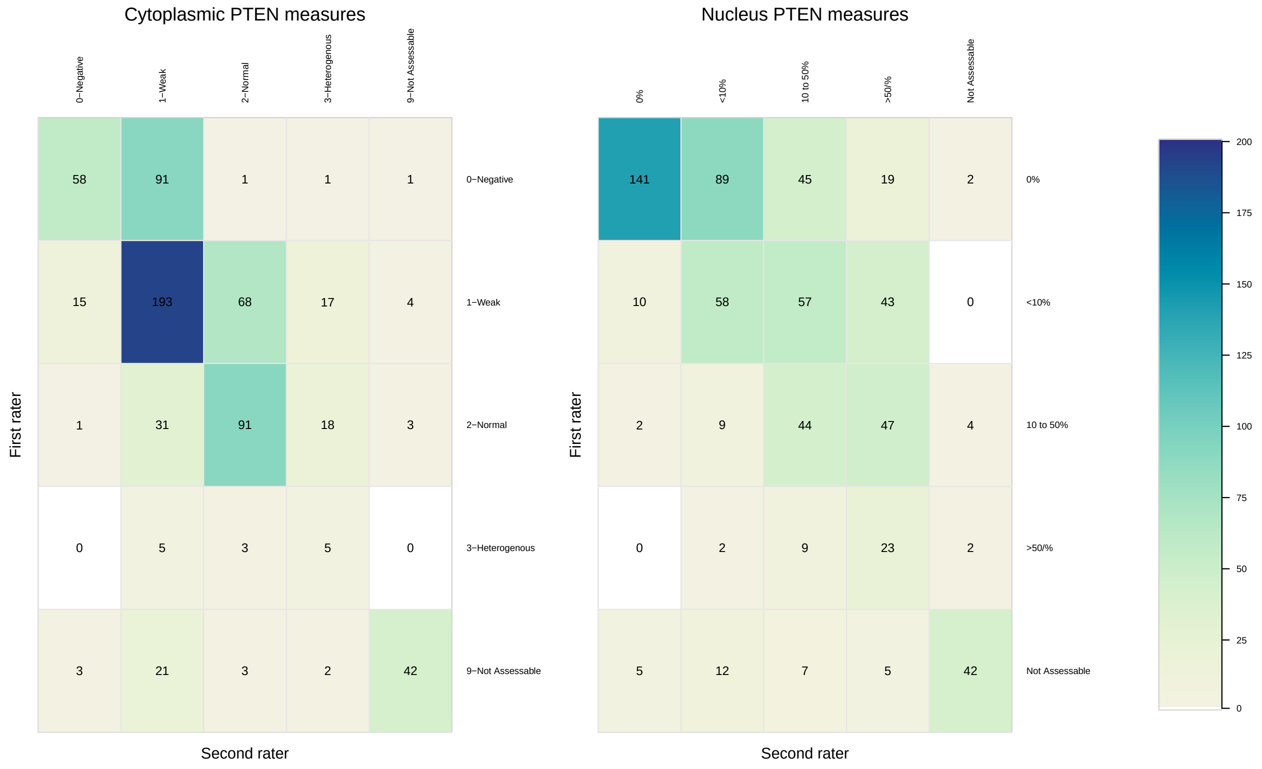

For cytoplasmic PTEN, at the exception of the level *heterogenous*, most cases belong to the diagonal or to positions next to the diagonal.

3 Agreement analyses

In this section, we define different estimated Cohen's agreement coefficients for the cytoplasmic and nucleus measures:

- (unweighted) global ,
- weighted for the ordinal levels of measure of interest,
- (unweighted) for dichotomous variable *heterogeneity* (only for cytoplasmic PTEN measures),
- (unweighted) for dichotomous variable *non-assessability*.

Parameter confidence intervals were defined by means of a non-parametric boostrap (with 2500 replicates). In agreement analyses, 0 corresponds to a zero agreement level (matches are due to chance) and 1 (or -1) corresponds to a perfect agreement (or perfect disagreement). The wikipedia page dedicated to Cohen's https://en.wikipedia.org/wiki/Cohen's_kappa mentions two useful magnitude guidelines :

- Landis and Koch :
  - [0; 0.2[slight,
  - [0.2; 0.4[fair, [0.4; .6[moderate,
  - [0.6; 0.8[substantial,

  - [0.8; 1[almost perfect,
- Fleiss :
  - [0; 40[poor,
  - [0.4; 0.75[fair to good,
  - [0.75; 1] excellent.

```
# estimation and inference
if(any(dir("results/rdata/")=="2-boot.rd")){
    load("results/rdata/2-boot.rd")
}else{
    source("source/2-bootstrap-kappa.r")
}
```

```
## 50  over  2500
## 100  over  2500
## 150  over  2500
## 200  over  2500
## 250  over  2500
## 300  over  2500
## 350  over  2500
## 400  over  2500
## 450  over  2500
## 500  over  2500
## 550  over  2500
## 600  over  2500
## 650  over  2500
## 700  over  2500
## 750  over  2500
## 800  over  2500
## 850  over  2500
## 900  over  2500
## 950  over  2500
## 1000  over  2500
## 1050  over  2500
## 1100  over  2500
## 1150  over  2500
## 1200  over  2500
## 1250  over  2500
## 1300  over  2500
## 1350  over  2500
## 1400  over  2500
## 1450  over  2500
## 1500  over  2500
## 1550  over  2500
## 1600  over  2500
```

```
## 1650  over  2500
## 1700  over  2500
## 1750  over  2500
## 1800  over  2500
## 1850  over  2500
## 1900  over  2500
## 1950  over  2500
## 2000  over  2500
## 2050  over  2500
## 2100  over  2500
## 2150  over  2500
## 2200  over  2500
## 2250  over  2500
## 2300  over  2500
## 2350  over  2500
## 2400  over  2500
## 2450  over  2500
## 2500  over  2500
```

We also produce agreement charts between raters 1 and 2 for both measures. The conclusion remains the same: For nucleus for example, the shift of the white rectangles corresponding to the ordinal levels of the scores over the diagonal indicates that rater 2 tends to classify cores into more severe diagnostic categories compared to rater 1.

3.1 Cytoplasmic results

```
data.frame(ar.kappa.r3o[,,"Cytoplasm"])
```

```
# plot
rownames(table_cytoplasm) = colnames(table_cytoplasm) =  c("Negative","Weak","Normal","Heterogenous","No
agreementplot(t(table_cytoplasm),weights=1,reverse_y=FALSE,main="Cytoplasm",
      xlab="",ylab="",pop=FALSE,xlab_rot=90,ylab_rot=0,xlab_just="right",ylab_just="right",
      xscale=FALSE,yscale=FALSE)
```

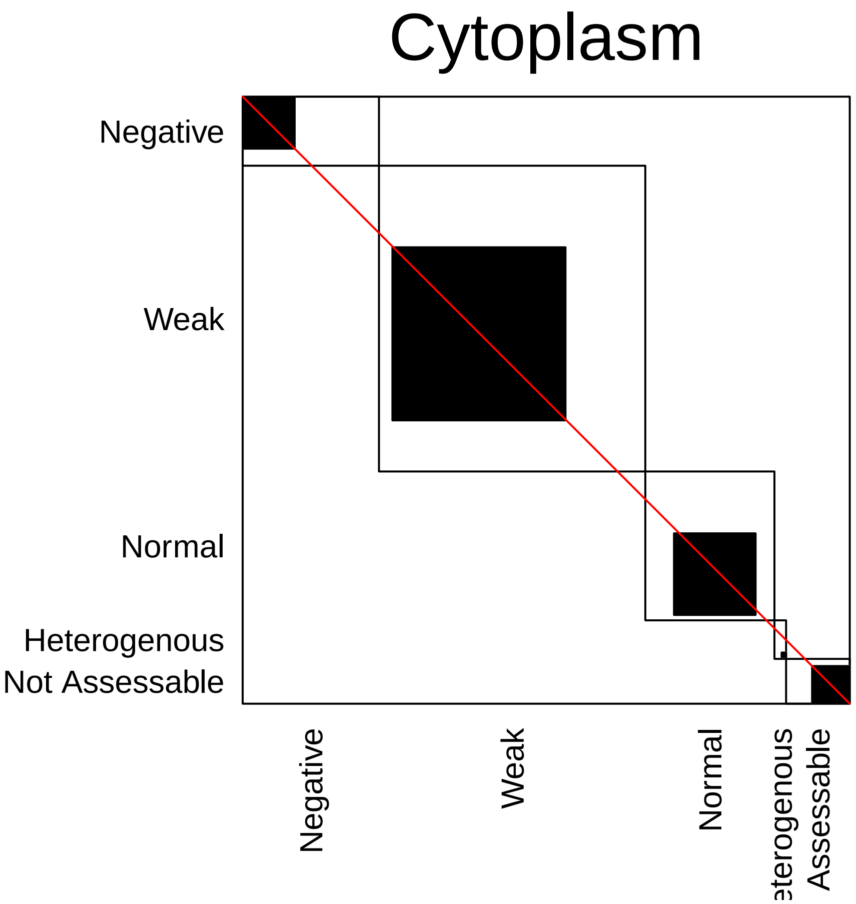


```
#axis(2,at=1,"First rater",tick=FALSE,cex=2,col.axis="blue",padj=-8)
#axis(1,at=1,"Second rater",tick=FALSE,cex=2,col.axis="blue",padj=8,hadj=-.75)
```

3.2 Nucleus results

```
data.frame(ar.kappa.r3o[-3,,"Nucleus"])
```

```
# plot
rownames(table_nucleus) = colnames(table_nucleus) =  c("0%","<10%","10 to 50%",">50%","Not Assessable")
agreementplot(t(table_nucleus),weights=1,reverse_y=FALSE,main="Nucleus",
      xlab="",ylab="",pop=FALSE,xlab_rot=90,ylab_rot=0,xlab_just="right",ylab_just="right",
      xscale=FALSE,yscale=FALSE)
```

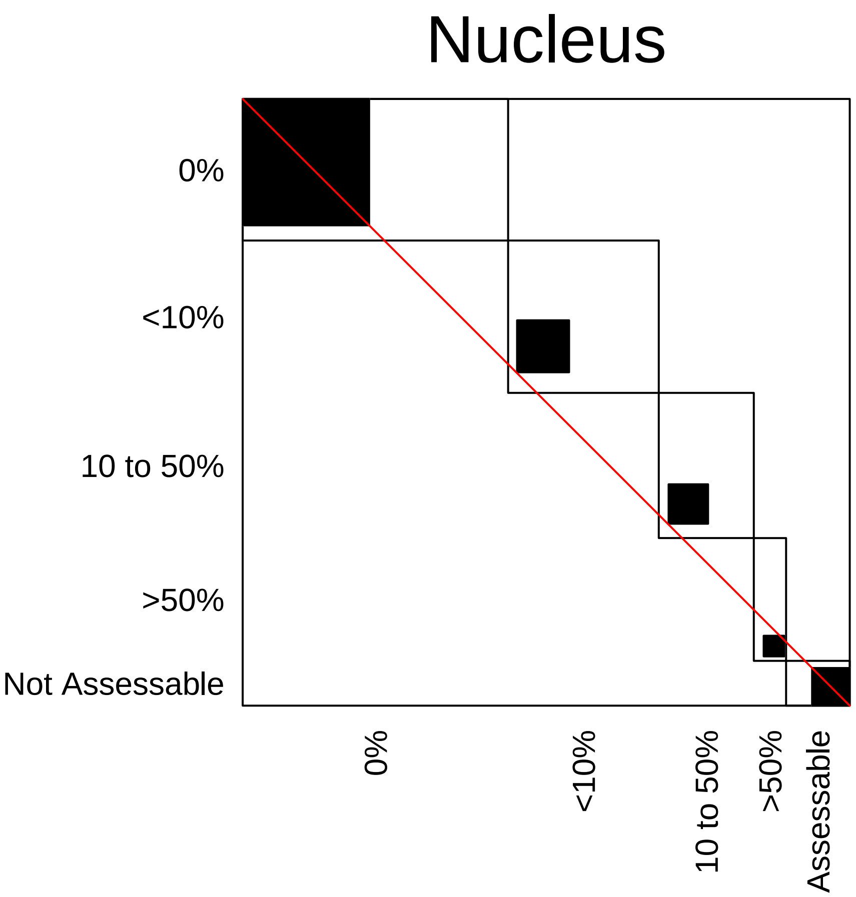


```
#axis(2,at=1,"First rater",tick=FALSE,cex=2,col.axis="blue",padj=-8)
#axis(1,at=1,"Second rater",tick=FALSE,cex=2,col.axis="blue",padj=8,hadj=-.75)
```

3.3 Combine results and save table

The following code produces the **Table S2 of the article**.

```
out = cbind(round(ar.kappa.r3o[,1,"Cytoplasm"],3),
            apply(round(ar.kappa.r3o[,2:3,"Cytoplasm"],3),1,function(x)paste0("[",x[1],";",x[2],"]")),
            round(ar.kappa.r3o[,1,"Nucleus"],3),
            apply(round(ar.kappa.r3o[,2:3,"Nucleus"],3),1,function(x)if(!is.na(x[1])){paste0("[",x[1],"
                  }else{NA})
            )
out[is.na(out)] = ""
colnames(out) = paste0(rep(c("Estimate","95%CI"),2),rep(c("_cyt","_nuc"),each=2))
# print
as.data.frame(out)
```

```
# save table as csv file
write.csv(out,file=paste0("results/tables/2-ratersagreement.csv"))
```

Script 3 - Association

D.-L. Couturier / F. Martins / J. Brenton / P. Pharoah

Last modified: 13 Mar 2020

```
#   Input data files    :   results/rdata/1-otta-amended.rd
#   Output data files :     -
#   Required R packages :   coin, colorspace
```

In this script, we analyse the association

- between *cancer type* and the biomarkers *PTEN* (cytoplasmic and nucleus), *CD8*, *PR*, *AR*, *ER*,
- between *cytoplasmic PTEN* and selected factors per *cancer type*.

1 Association between cancer type and different biomarkers

We use the χ^2- test to assess if *cancer type* and the biomarker of interest are dependent. When the association is significant, we analyse the association visually by looking at

- the sign (blue for negative, red for positive) and
- value (coloured for large, grey for small) of the (signed) square root of the contribution of each element of the contingency table of interest to the χ^2-test statistic.

1.1 Association between cancer type and cytoplasmic PTEN

Figure 1C of the article

```
chisq.test(table(otta$pten4_cytoplasm,otta$cancer_type))
```

```
##
##  Pearson's Chi-squared test
##
## data:  table(otta$pten4_cytoplasm, otta$cancer_type)
## X-squared = 183.87, df = 12, p-value < 2.2e-16
```

```
CMHtest(table(otta$pten4_cytoplasm,otta$cancer_type))
```

```
## Cochran-Mantel-Haenszel Statistics for  by
##
##                  AltHypothesis    Chisq Df       Prob
## cor        Nonzero correlation   2.6475  1 1.0371e-01
## rmeans  Row mean scores differ  15.0054  3 1.8121e-03
## cmeans  Col mean scores differ 130.2161  4 3.5009e-27
## general    General association 183.8304 12 6.9743e-33
```

```
# mosaic plot
par(mfrow=c(1,1),mar=c(2.5,0,0,2.5))
y = otta$pten4_cytoplasm_r
x = otta$cancer_type
mosaicplot(table(x,y),main="",
```

```
       color = TRUE, shade = c(2,3,4,5),
       xlab = "",ylab="",las=2,dir="v",cex.axis=.725)
axis(1,at=0.55,tick=FALSE,labels="Cancer Type",cex.axis=1.3)
axis(4,at=0.45,tick=FALSE,labels="PTEN",cex.axis=1.3,las=2,hadj=.6)
```

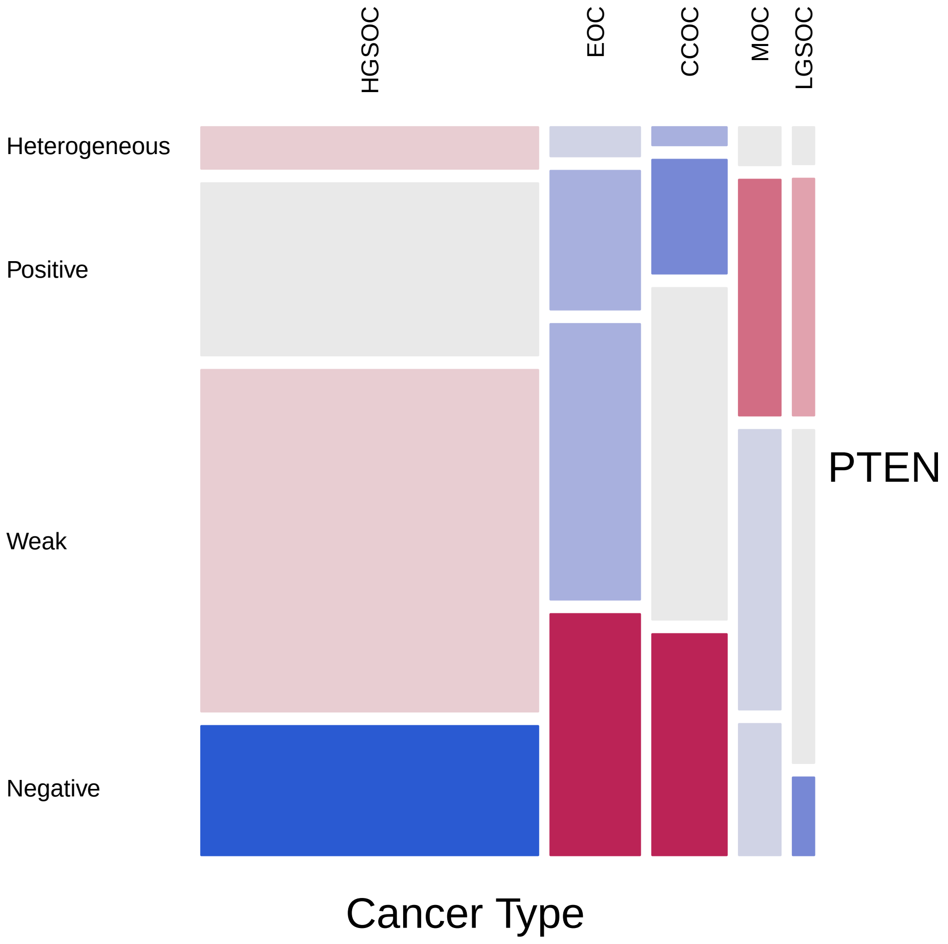

1.2 Association between cancer type and nucleus PTEN

```
chisq.test(table(otta$pten4_nucleus,otta$cancer_type))
```

```
##
##  Pearson's Chi-squared test
##
## data:  table(otta$pten4_nucleus, otta$cancer_type)
## X-squared = 125.68, df = 12, p-value < 2.2e-16
```

```
# mosaic plot
par(mfrow=c(1,1),mar=c(2.5,0,0,2.5))
mosaicplot(table(otta$cancer_type,otta$pten4_nucleus),
       main="", color=TRUE, shade = c(2,3,4,5),
       xlab = "",ylab="",las=2,dir="v",cex.axis=.7)
axis(1,at=0.55,tick=FALSE,labels="Cancer Type",cex.axis=1.25)
```

```r
axis(4,at=0.475,tick=FALSE,labels="PTEN",cex.axis=1.25,las=2,hadj=.75)
```

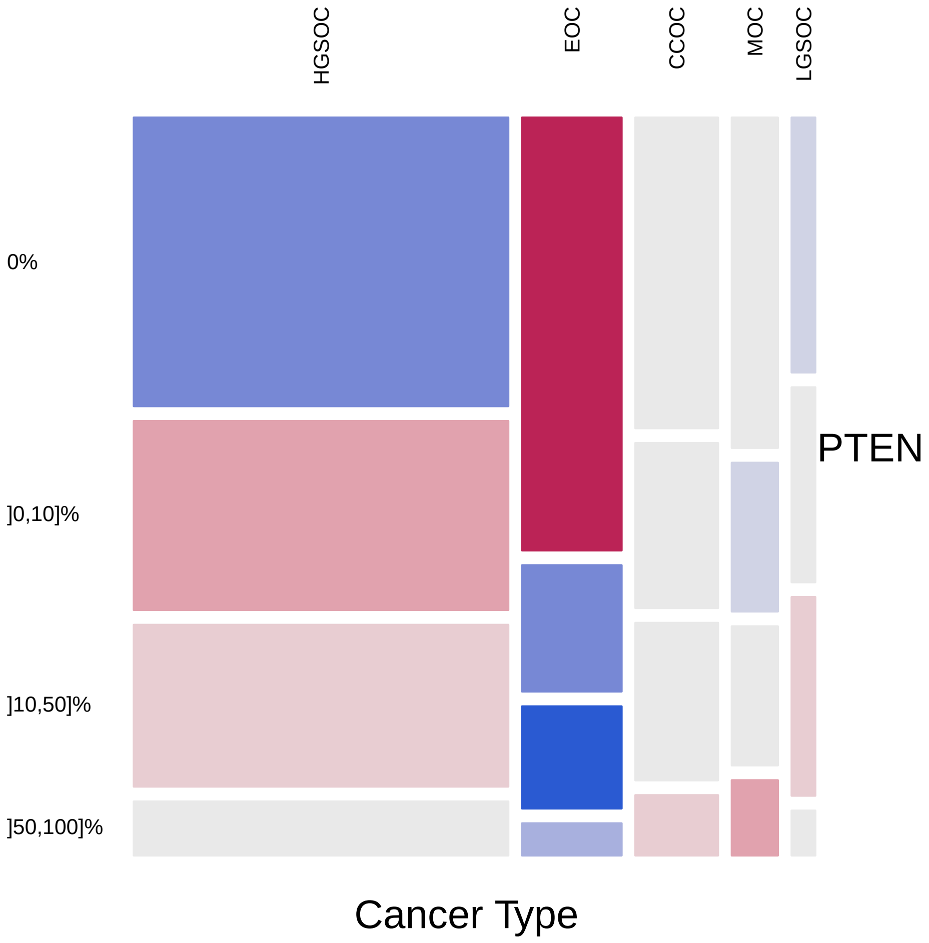

1.3 Association between cancer type and CD8

Figure 2C of the article

```r
chisq.test(table(otta$cd84,otta$cancer_type))
```

```
##
##  Pearson's Chi-squared test
##
## data:  table(otta$cd84, otta$cancer_type)
## X-squared = 485.16, df = 12, p-value < 2.2e-16
```

```r
# mosaic plot
y = otta[,"cd84_r"]
x = otta[,"cancer_type"]
par(mfrow=c(1,1),mar=c(2.5,0,0,4))
mosaicplot(table(x,y),main="",
       color = TRUE, shade = c(2,3,4,5),
       xlab = "",ylab="",las=2,dir="v",cex.axis=.725)
axis(1,at=0.55,tick=FALSE,labels="Cancer Type",cex.axis=1.3)
```

```r
axis(4,at=0.45,tick=FALSE,labels="CD8",cex.axis=1.3,las=2,hadj=.4)
```

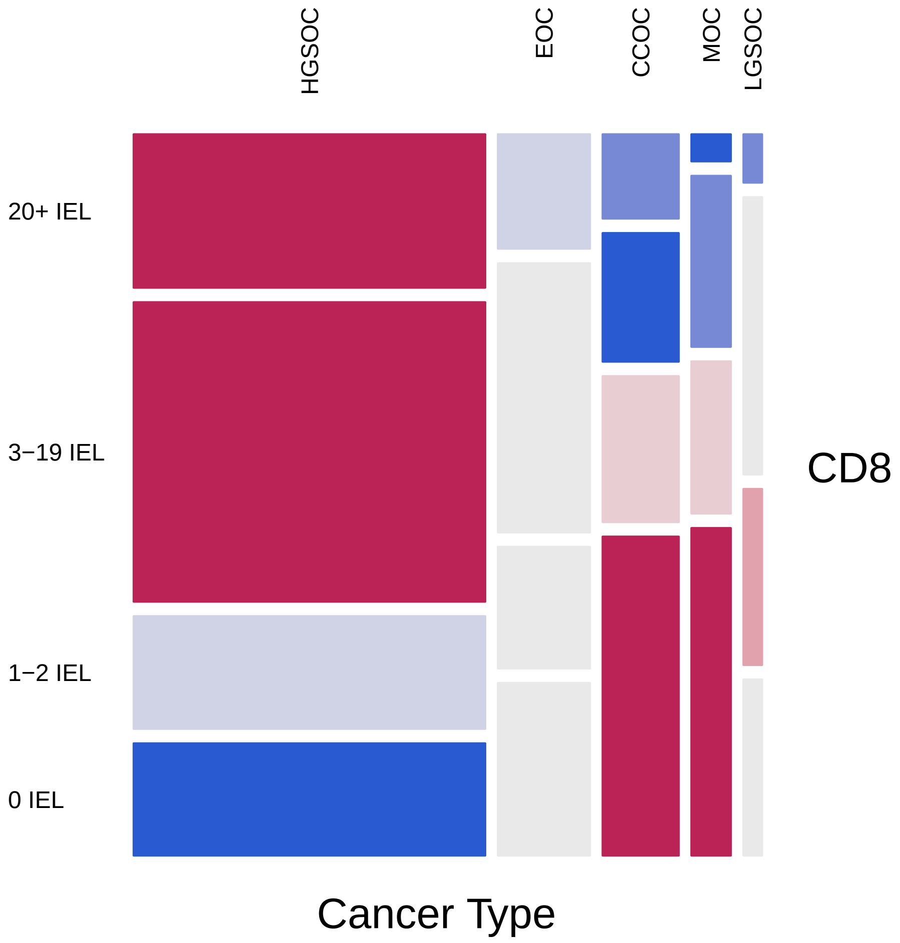

1.4 Association between cancer type and AR

```r
chisq.test(table(otta$ar2,otta$cancer_type))
```

```
##
##  Pearson's Chi-squared test
##
## data:  table(otta$ar2, otta$cancer_type)
## X-squared = 315.01, df = 4, p-value < 2.2e-16
```

```r
# mosaic plot
par(mfrow=c(1,1),mar=c(2.5,0,0,2.5))
mosaicplot(table(otta$cancer_type,otta$ar2),main="",
       color = TRUE, shade = c(2,3,4,5),
       xlab = "",ylab="",las=2,dir="v",cex.axis=.7)
axis(1,at=0.55,tick=FALSE,labels="Cancer Type",cex.axis=1.25)
axis(4,at=0.475,tick=FALSE,labels="AR",cex.axis=1.25,las=2,hadj=.75)
```

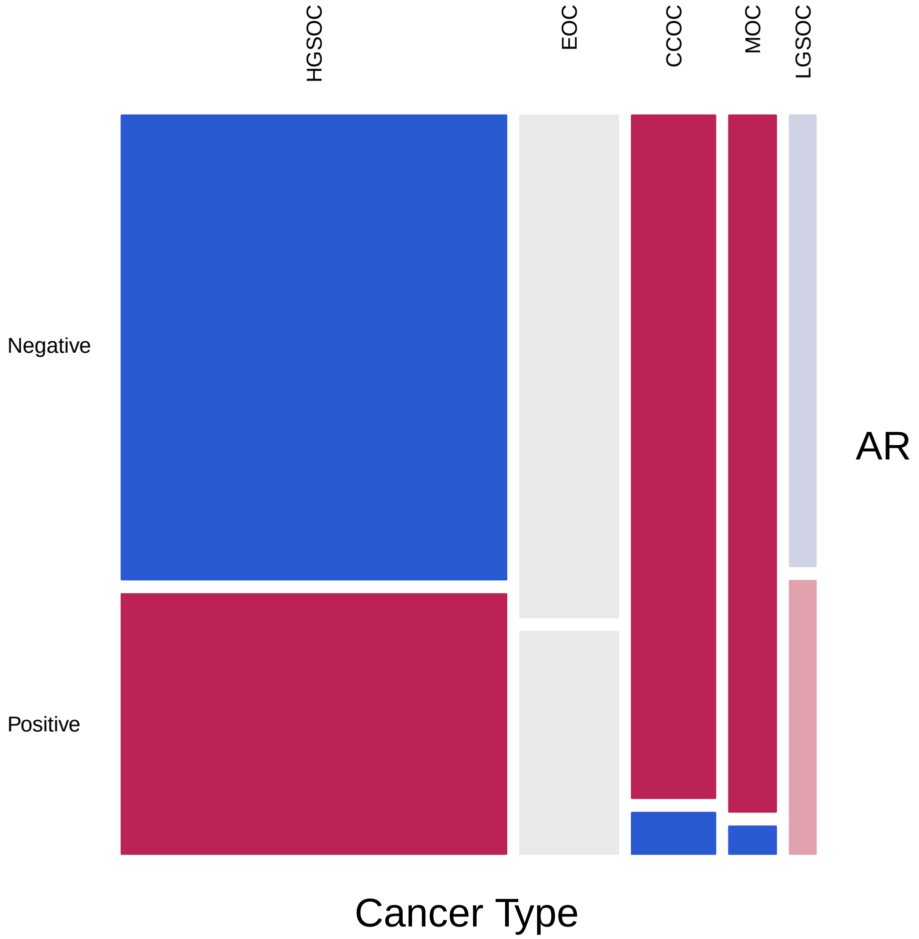

1.5 Association between cancer type and PR

```
chisq.test(table(otta$pr3,otta$cancer_type))
```

```
##
##  Pearson's Chi-squared test
##
## data:  table(otta$pr3, otta$cancer_type)
## X-squared = 976.95, df = 8, p-value < 2.2e-16
```

```
# mosaic plot
par(mfrow=c(1,1),mar=c(2.5,0,0,2.5))
mosaicplot(table(otta$cancer_type,otta$pr3),main="",
       color = TRUE, shade = c(2,3,4,5),
       xlab = "",ylab="",las=2,dir="v",cex.axis=.7)
axis(1,at=0.55,tick=FALSE,labels="Cancer Type",cex.axis=1.25)
axis(4,at=0.475,tick=FALSE,labels="PR",cex.axis=1.25,las=2,hadj=.75)
```

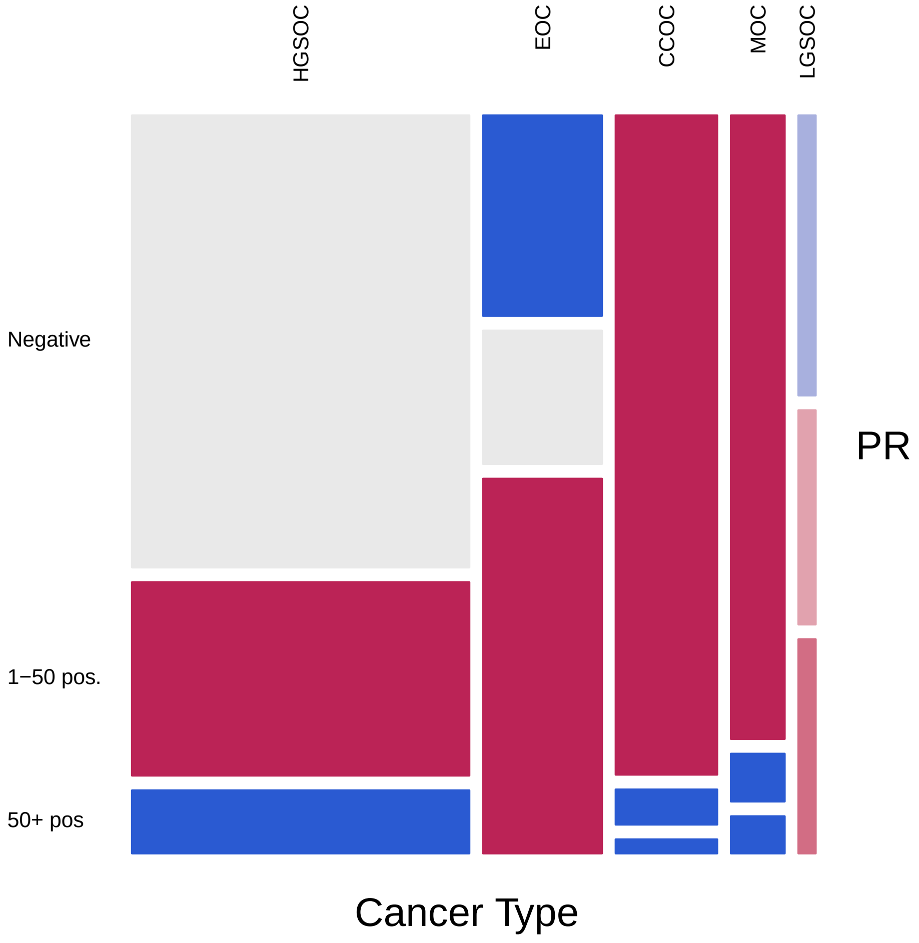

1.6 Association between cancer type and ER

```
chisq.test(table(otta$er3,otta$cancer_type))
```

```
##
##  Pearson's Chi-squared test
##
## data:  table(otta$er3, otta$cancer_type)
## X-squared = 614.33, df = 8, p-value < 2.2e-16
```

```
# mosaic plot
par(mfrow=c(1,1),mar=c(2.5,0,0,2.5))
mosaicplot(table(otta$cancer_type,otta$er3),main="",
       color = TRUE, shade = c(2,3,4,5),
       xlab = "",ylab="",las=2,dir="v",cex.axis=.7)
axis(1,at=0.55,tick=FALSE,labels="Cancer Type",cex.axis=1.25)
axis(4,at=0.475,tick=FALSE,labels="ER",cex.axis=1.25,las=2,hadj=.75)
```

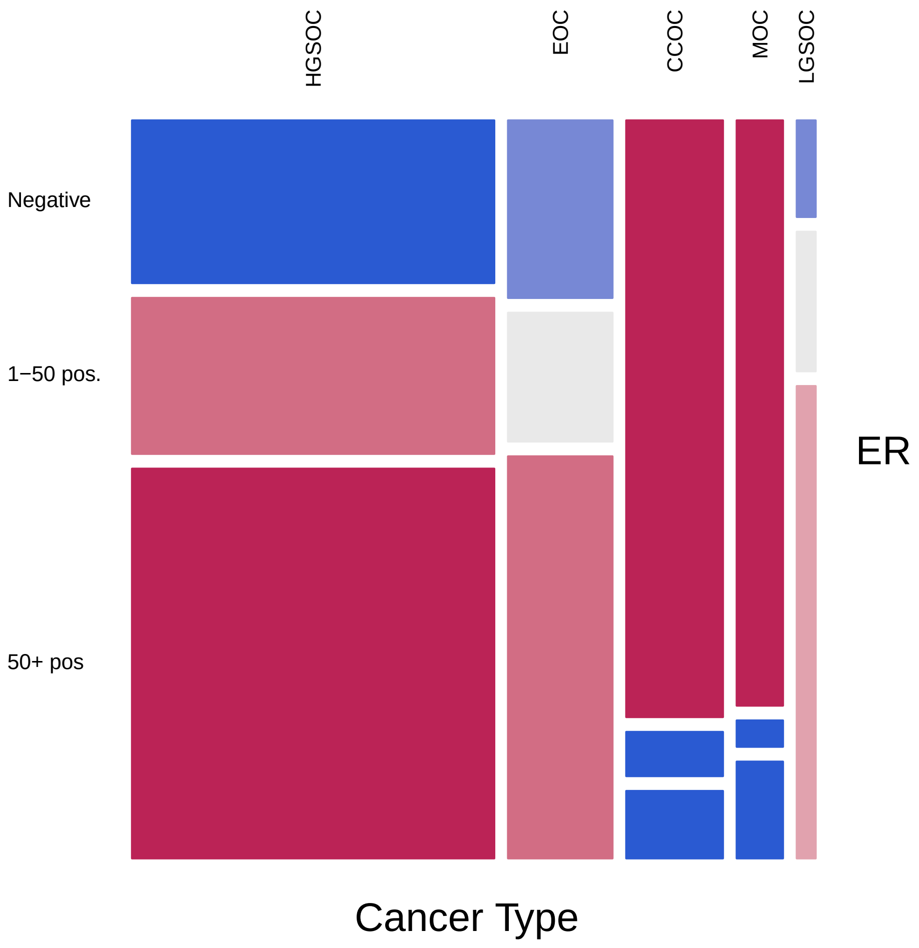

2 Association between cytoplasmic PTEN score and selected biomarkers and ordinal variables per Cancer type

In this section, the association between *cytoplasmic PTEN scores* and selected ordinal variables available in the OTTA dataset is tested as follows:

- **Nominal/Nominal**: *cytoplasmic PTEN* as a 4-level **nominal** variable, the other variable as a **nominal** factor, and performing a Pearson's Chi-square test (with simulated p-values, based on 500'000 Monte Carlo replicates, to get p-values valid even if the commonly used rules of thumbs suggest not to use the Chi-square test),
- **Nominal/Ordinal**: *cytoplasmic PTEN* as a 4-level **nominal** variable, the other variable as an **ordinal** factor, and performing an *extended Cochran -Armitage trend* (also referred to as a *Generalized Pearson Chi-Squared Test* and related to the linear logit model. refer to Agresti, 2002, p182),
- **Ordinal/Ordinal**: *cytoplasmic PTEN* as a 3-level **ordinal** variable (by ignoring the level *heterogenous*), the other variable as an **ordinal** factor, and performing a *linear-by-linear ordinal association test* (refer to Agresti, 2002, p86)

Note

- that these 3 options are special cases of the *generalised Cochran-Mantel-Haenszel test* (refer to Agresti, 2010, p205), thanks to the unifying work of Landis et al, 1978).

- that we chose to assume equidistant scores when considering ordinal variables,
- that we also compare the Chi-Square test to the Fisher one (with simulated p-values when the dataset is too large),
- that the **Nominal/Ordinal** and **Ordinal/Ordinal** cases are more efficient (under the model) as less degrees of freedom are required.

2.1 Estimation

```
## var list:
listvarw = c("tumourafter","differentiation2","figo",
             "agegroupatdiagnosis","cd84","ar2","pr3","er3")
n.var = length(listvarw)
id.var = data.frame(pos=1:n.var,id=listvarw,
                    name = c("Tumour after treatment","Differentiation level",
                             "FIGO staging","Age group at diagnosis",
                             "CD8","AR","PR","ER"),
                    abbr = c("t","d","f","g","c","a","p","r"),
                    stringsAsFactors=FALSE)
## cancer:
n.cancer  = nlevels(otta$cancer_type)
id.cancer = data.frame(pos=1:n.cancer,id=levels(otta$cancer_type),
                       name = levels(otta$cancer_type2),
                       stringsAsFactors=FALSE)

##
n.test  = 10
id.test = data.frame(pos = 1:n.test,
                     id  = c("chisq-asym","chisq-sim",
                             "fisher-exact","fisher-sim",
                             "cochran-armitage",
                             "linear-by-linear",
                             "gCMH-nom+nom",
                             "gCMH-nom+ord",
                             "gCMH-ord+nom",
                             "gCMH-ord+ord"),
                     stringsAsFactors=FALSE)

##
ar.pval.vct = array(NA,dim=c(n.var,n.cancer,n.test),
                    dimnames=list(id.var$id,id.cancer$id,id.test$id))
mx.warning.vc = matrix(FALSE,nrow=n.var,ncol=n.cancer,
                       dimnames=list(id.var$id,id.cancer$id))
set.seed(30)
for(cw in 1:n.cancer){
    data_cw = otta[as.numeric(otta$cancer_type)==cw,]
    for(vw in 1:n.var){
        observed = table(v1=data_cw[,id.var$id[vw]],
                         pten = data_cw[,"pten4_cytoplasm"])
        expected = t(sapply(apply(observed,1,sum),
                     function(x)x*apply(observed,2,sum))/sum(observed))
            if(!all(is.na(expected))){
            # warning
            if(any(expected<5)){mx.warning.vc[vw,cw] = TRUE}
```

```
            # test1
            testw = try(chisq.test(observed),silent=TRUE)
            if(class(testw)!="try-error"){
                ar.pval.vct[vw,cw,1] = testw$p.value
                }
            # test2
            testw = try(chisq.test(observed,simulate.p.value = TRUE,B = 500000))
            if(class(testw)!="try-error"){
                ar.pval.vct[vw,cw,2] = testw$p.value
                }
            # test3
            testw = try(fisher.test(observed),silent=TRUE)
            if(class(testw)!="try-error"){
                ar.pval.vct[vw,cw,3] = testw$p.value
                }
            # test4
            testw = try(fisher.test(observed,workspace = 2000000,
                                    simulate.p.value = TRUE,B = 500000))
            if(class(testw)!="try-error"){
                ar.pval.vct[vw,cw,4] = testw$p.value
                }
            # test5
            testw = try(chisq_test(observed,
                        scores = list("v1" = 1:nlevels(otta[,id.var$id[vw]]))))
            if(class(testw)!="try-error"){
                ar.pval.vct[vw,cw,5] = pvalue(testw)
                }
            # test6
            testw = try(lbl_test(observed[,c("Negative","Weak","Positive")]))
            if(class(testw)!="try-error"){
                ar.pval.vct[vw,cw,6] = pvalue(testw)
                }
            # tests7+9
            testw = try(CMHtest(observed))
            if(class(testw)!="try-error"){
                ar.pval.vct[vw,cw,c("gCMH-nom+nom","gCMH-ord+nom")] = testw[[1]][c("general","cmeans"),
                }
            # tests7+9
            testw = try(CMHtest(observed[,c("Negative","Weak","Positive")]))
            if(class(testw)!="try-error"){
                ar.pval.vct[vw,cw,c("gCMH-nom+ord","gCMH-ord+ord")] = testw[[1]][c("rmeans","cor"),"Prol
                }
            }
        }# end vw
    }# end cw

# adjust p-value
ar.pval_holm1.vct = apply(ar.pval.vct,c(2:3),p.adjust,method="holm")
```

2.2 Checks

```r
# check: compare p-values of the chi-square test
#        obtained by simulations (Hope 1968) or by means of
#        asymptotic inference
par(mfcol=c(2,3),mar=c(4,4,2,2))
#
pvalw = c(0.0001,0.001,0.01,0.05,.5)
colw  = rep(1:5,each=n.var)
labw  = rep(id.var$abbr,n.var)
pchw  = c(NA,19)[as.numeric(ar.pval_holm1.vct[,,"linear-by-linear"]<0.05)+1]
#
plot(log(ar.pval.vct[,,"chisq-asym"]),log(ar.pval.vct[,,"chisq-sim"]),
     xlab="log p-val of Chi2 (asymp)",ylab="log p-val of Chi2 (sim)",
     axes=FALSE,pch=pchw,cex=2,col="light gray")
text(log(ar.pval.vct[,,"chisq-asym"]),log(ar.pval.vct[,,"chisq-sim"]),
     col=colw,labels=labw)
axis(1,log(pvalw),pvalw,tick=TRUE,las=2,cex.axis=.75)
axis(2,log(pvalw),pvalw,tick=TRUE,las=2,cex.axis=.75)
box()
abline(0,1,col="red")
abline(h=log(0.05),lty=3,col="gray")
abline(v=log(0.05),lty=3,col="gray")
#
plot(log(ar.pval.vct[,,"fisher-sim"]),log(ar.pval.vct[,,"chisq-sim"]),
     xlab="log p-val of Fisher (sim)",ylab="log p-val of Chi2 (sim)",
     axes=FALSE,pch=pchw,cex=2,col="light gray")
text(log(ar.pval.vct[,,"fisher-sim"]),log(ar.pval.vct[,,"chisq-sim"]),
     col=colw,labels=labw)
axis(1,log(pvalw),pvalw,tick=TRUE,las=2,cex.axis=.75)
axis(2,log(pvalw),pvalw,tick=TRUE,las=2,cex.axis=.75)
box()
abline(0,1,col="red")
abline(h=log(0.05),lty=3,col="gray")
abline(v=log(0.05),lty=3,col="gray")
#
plot(log(ar.pval.vct[,,"cochran-armitage"]),log(ar.pval.vct[,,"chisq-sim"]),
     xlab="extended Cochran-Armitage trend",ylab="log p-val of Chi2 (sim)",
     axes=FALSE,pch=pchw,cex=2,col="light gray")
text(log(ar.pval.vct[,,"cochran-armitage"]),log(ar.pval.vct[,,"chisq-sim"]),
     col=colw,labels=labw)
axis(1,log(pvalw),pvalw,tick=TRUE,las=2,cex.axis=.75)
axis(2,log(pvalw),pvalw,tick=TRUE,las=2,cex.axis=.75)
box()
abline(0,1,col="red")
abline(h=log(0.05),lty=3,col="gray")
abline(v=log(0.05),lty=3,col="gray")
#
plot(log(ar.pval.vct[,,"linear-by-linear"]),log(ar.pval.vct[,,"chisq-sim"]),
     xlab="Linear-by-linear ordinal association",ylab="log p-val of Chi2 (sim)",
     axes=FALSE,pch=pchw,cex=2,col="light gray")
text(log(ar.pval.vct[,,"linear-by-linear"]),log(ar.pval.vct[,,"chisq-sim"]),
     col=colw,labels=labw)
```

```
axis(1,log(pvalw),pvalw,tick=TRUE,las=2,cex.axis=.75)
axis(2,log(pvalw),pvalw,tick=TRUE,las=2,cex.axis=.75)
box()
abline(0,1,col="red")
abline(h=log(0.05),lty=3,col="gray")
abline(v=log(0.05),lty=3,col="gray")
#
plot(1,1,pch="",xlab="",ylab="",axes=FALSE)
legend("top",ncol=1,lty=1,col=1:5,
       levels(otta$cancer_type))
legend("bottom",ncol=1,pch=19,col="light gray",
       legend=c("Significant\n[linear-by linear association]"),cex=.75)
#
plot(1,1,pch="",xlab="",ylab="",axes=FALSE)
legend("top",ncol=1,col="black",
       paste0(id.var$abbr," = ",id.var$name),cex=.75)
```

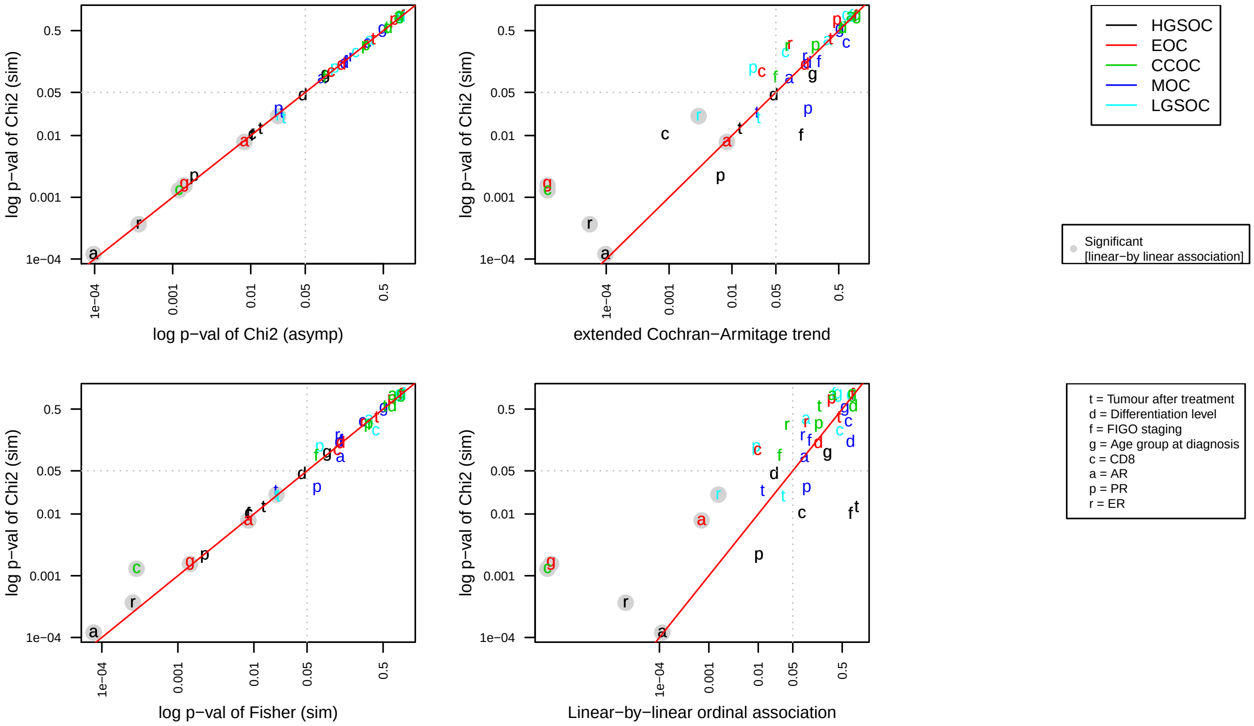

Comments:

- the upper left plot shows that the simulated and asymptotic p-values for the Chi-square lead to the same conclusions in this case,
- the bottom left plot, comparing the Chi-square and Fisher test results, suggests that the only different conclusion, at the 5% alpha level, occurs when analysing the association between cytoplasmic PTEN and PR for the MOC cancer type,
- the right upper plot shows that there is not much benefit in using a test not ignoring the ordinal characteristics of the variables other than PTEN.
- the right bottom plot shows the benefits of using a test not ignoring the ordinal characteristics of the both variables (many p-values are smaller for the linear-by-linear association model).
- the light grey circles show the associations which are found as significant with a global Holm multiplicity correction according to the linear-by-linear association model.

```r
# check: compare p-values of the chi-square test
#        obtained by simulations (Hope 1968) or by means of
#        asymtotical inference
par(mfcol=c(2,2),mar=c(4,4,2,2))
#
pvalw = c(0.0001,0.001,0.01,0.05,.5)
colw  = rep(1:5,each=n.var)
labw  = rep(id.var$abbr,n.var)
#
plot(log(ar.pval.vct[,,"chisq-asym"]),log(ar.pval.vct[,,"gCMH-nom+nom"]),
     xlab="log p-val of Chi2 (asymp)",ylab="log p-val of gCMH Nominal/Nominal",
     axes=FALSE,cex=2,pch="",col="light gray",main="Nominal/Nominal")
text(log(ar.pval.vct[,,"chisq-asym"]),log(ar.pval.vct[,,"gCMH-nom+nom"]),
     col=colw,labels=labw)
axis(1,log(pvalw),pvalw,tick=TRUE,las=2,cex.axis=.75)
axis(2,log(pvalw),pvalw,tick=TRUE,las=2,cex.axis=.75)
box()
abline(0,1,col="red")
abline(h=log(0.05),lty=3,col="gray")
abline(v=log(0.05),lty=3,col="gray")
#
plot(log(ar.pval.vct[,,"cochran-armitage"]),log(ar.pval.vct[,,"gCMH-ord+nom"]),
     xlab="log p-val of cochran-armitage",ylab="log p-val of gCMH Ordinal/Nominal",
     axes=FALSE,cex=2,pch="",col="light gray", main="Ordinal(OTHER)/Nominal(PTEN)")
text(log(ar.pval.vct[,,"cochran-armitage"]),log(ar.pval.vct[,,"gCMH-ord+nom"]),
     col=colw,labels=labw)
axis(1,log(pvalw),pvalw,tick=TRUE,las=2,cex.axis=.75)
axis(2,log(pvalw),pvalw,tick=TRUE,las=2,cex.axis=.75)
box()
abline(0,1,col="red")
abline(h=log(0.05),lty=3,col="gray")
abline(v=log(0.05),lty=3,col="gray")
#
plot(log(ar.pval.vct[,,"linear-by-linear"]),log(ar.pval.vct[,,"gCMH-ord+ord"]),
     xlab="log p-val of linear-by-linear",ylab="log p-val of gCMH Ordinal/Ordinal",
     axes=FALSE,pch="",cex=2,col="light gray", main="Ordinal/Ordinal")
text(log(ar.pval.vct[,,"linear-by-linear"]),log(ar.pval.vct[,,"gCMH-ord+ord"]),
     col=colw,labels=labw)
axis(1,log(pvalw),pvalw,tick=TRUE,las=2,cex.axis=.75)
axis(2,log(pvalw),pvalw,tick=TRUE,las=2,cex.axis=.75)
box()
abline(0,1,col="red")
abline(h=log(0.05),lty=3,col="gray")
abline(v=log(0.05),lty=3,col="gray")
#
#
plot(1,1,pch="",xlab="",ylab="",axes=FALSE)
legend("top",ncol=1,lty=1,col=1:5,
       levels(otta$cancer_type),cex=.5)
#
legend("bottom",ncol=1,col="black",
       paste0(id.var$abbr," = ",id.var$name),cex=.5)
```

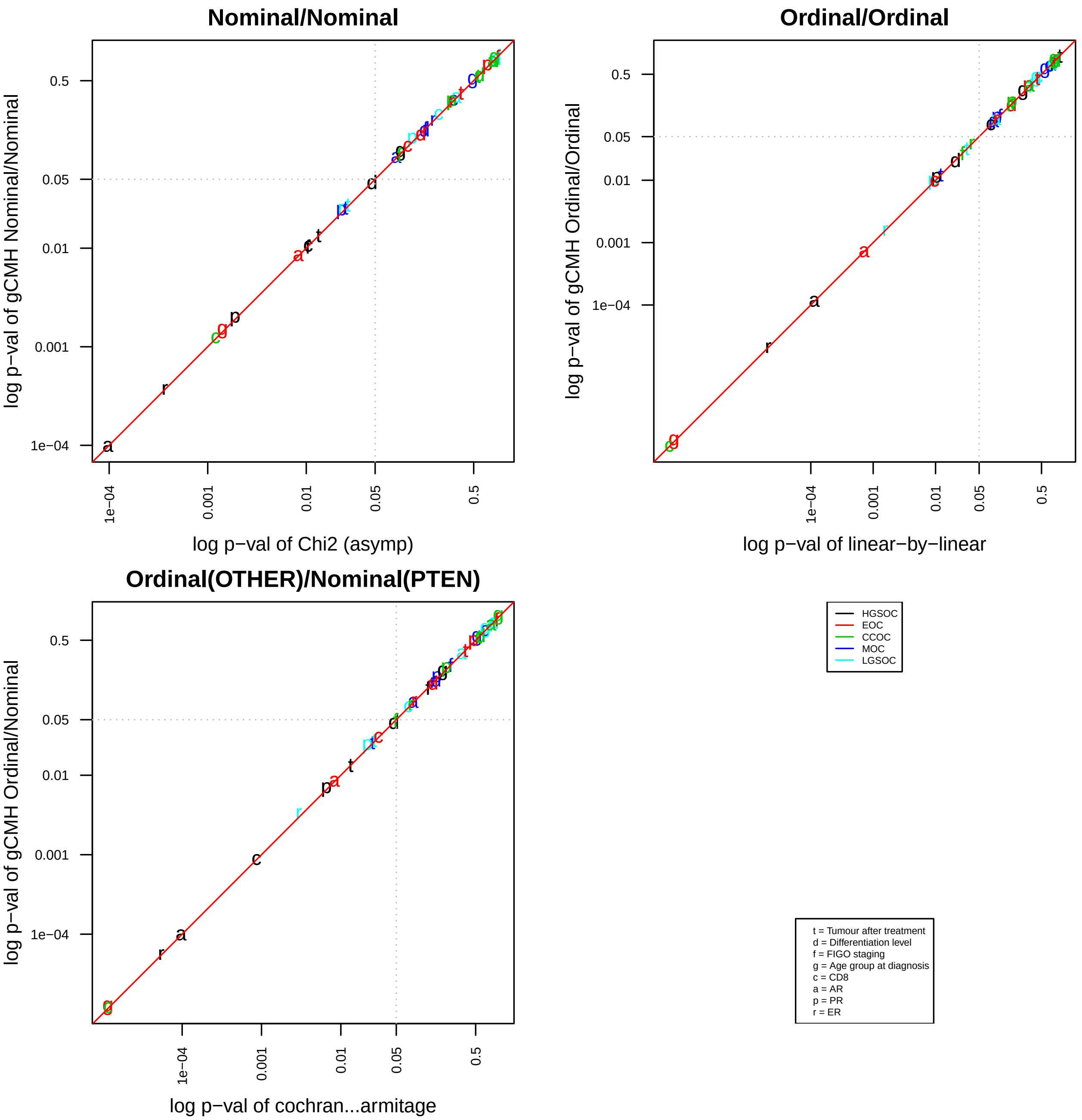

Comments:

- the generalised Cochran-Mantel-Haenszel tests as implemented in the package vcdExtra does the same job as the tests described above. Out of simplicity, it may be worth describing only one unifying test instead of three different ones.

2.3 Results with *Holm* multiplicity correction per cancer type

The following table (**Table S3 of the article**) shows the p-values of the different association models per variable (rows) and cancer type (columns) after muliplicity correction [Holm global multiplicity correction, 5% per analysis and cancer type].

```
ar.pval_holm1.vct = ar.pval_holm1.vct[,,c("gCMH-nom+nom","gCMH-ord+nom","gCMH-ord+ord")]
TableS3 = NULL
for(dw in 1:dim(ar.pval_holm1.vct)[3]){
    temp = data.frame(Variable = id.var$name,
                      Test = c("Nominal/Nominal","Nominal/Ordinal",
                      "Ordinal/Ordinal")[dw],
                      apply(ar.pval_holm1.vct[,,dw],2,pval.fun))
    TableS3 = rbind(TableS3,temp)
    }
TableS3 = TableS3[order(match(TableS3$Variable,id.var$name)),]
# print
data.frame(TableS3)
```

3 Additional analyses

In this section, we perform the tests related to two additional analyses aiming to describe the association between heterogenous PTEN and

- FIGO staging,
- Levels of CD8.
- Levels of CD8 by BRCA mutation status

Note that the p-values of these additional analyses are non-adjusted.

3.1 Association between heterogenous PTEN and FIGO staging

```
x = otta[otta$cancer_type=="HGSOC","figo"]
y = otta[otta$cancer_type=="HGSOC","pten4_cytoplasm_r"]
CMHtest(table(x,y=="Heterogeneous"))
```

```
## Cochran-Mantel-Haenszel Statistics for x by
##
##                  AltHypothesis  Chisq Df     Prob
## cor        Nonzero correlation 5.5311  1 0.018681
## rmeans  Row mean scores differ 6.3325  3 0.096508
## cmeans  Col mean scores differ 5.5311  1 0.018681
## general    General association 6.3325  3 0.096508
```

3.2 Association between heterogenous PTEN and CD8

```
x = otta[otta$cancer_type=="HGSOC","cd84"]
y = otta[otta$cancer_type=="HGSOC","pten4_cytoplasm_r"]
CMHtest(table(x,y=="Heterogeneous"))
```

```
## Cochran-Mantel-Haenszel Statistics for x by
##
##                  AltHypothesis   Chisq Df      Prob
## cor        Nonzero correlation  9.9158  1 0.0016387
## rmeans  Row mean scores differ 10.2638  3 0.0164519
```

```
## cmeans  Col mean scores differ  9.9158  1 0.0016387
## general    General association 10.2638  3 0.0164519
```

Generalised Cochran-Mantel-Haenszel test for the association between CD8 and PTEN:

```
# general association between CD8 and PTEN:
x = otta[otta$cancer_type=="HGSOC","cd84"]
y = otta[otta$cancer_type=="HGSOC","pten4_cytoplasm_r"]
CMHtest(table(x,y))
```

```
## Cochran-Mantel-Haenszel Statistics for x by y
##
##                  AltHypothesis  Chisq Df       Prob
## cor        Nonzero correlation 10.783  1 0.00102459
## rmeans  Row mean scores differ 13.493  3 0.00368274
## cmeans  Col mean scores differ 16.562  3 0.00086957
## general    General association 21.533  9 0.01048459
```

3.3 Association between heterogenous PTEN and CD8 by BRCA mutation status

Analysis suggested by reviewer 3:

```
# Figures
par(mfrow=c(2,2),mar=c(2.5,0,3.5,4))

    # general association between PTEN and mutation type
    x = otta[otta$cancer_type=="HGSOC","mutation_type"]
    y = otta[otta$cancer_type=="HGSOC","pten4_cytoplasm_r"]
    mosaicplot(table(x,y),main="All HGSOC cases",
           color = TRUE, shade = c(2,3,4,5),
           xlab = "",ylab="",las=2,dir="v",cex.axis=.725)
    axis(1,at=0.55,tick=FALSE,labels="Mutation type",cex.axis=1.3)
    axis(4,at=0.5,tick=FALSE,labels="PTEN",cex.axis=1.3,las=2,hadj=.6)
    axis(4,at=0.45,tick=FALSE,labels="(HGSOC)",cex.axis=1.2,las=2,hadj=.45)

    # general association between CD8 and PTEN for pathogenic (ie, BRCA 1/2) mutation:
    x = otta[otta$cancer_type=="HGSOC","cd84"]
    y = otta[otta$cancer_type=="HGSOC","pten4_cytoplasm_r"]
    mosaicplot(table(x,y),main="All HGSOC cases",
           color = TRUE, shade = c(2,3,4,5),
           xlab = "",ylab="",las=2,dir="v",cex.axis=.725)
    axis(1,at=0.55,tick=FALSE,labels="CD8",cex.axis=1.3)
    axis(4,at=0.5,tick=FALSE,labels="PTEN",cex.axis=1.3,las=2,hadj=.6)
    axis(4,at=0.45,tick=FALSE,labels="(HGSOC)",cex.axis=1.2,las=2,hadj=.45)

    # general association between CD8 and PTEN for pathogenic (ie, BRCA 1/2) mutation:
    x = otta[otta$cancer_type=="HGSOC"&otta$mutation_type=="Pathogenic","cd84"]
    y = otta[otta$cancer_type=="HGSOC"&otta$mutation_type=="Pathogenic","pten4_cytoplasm_r"]
    mosaicplot(table(x,y),main="Only HGSOC cases with\npathogenic mutations",
           color = TRUE, shade = c(2,3,4,5),
           xlab = "",ylab="",las=2,dir="v",cex.axis=.725)
    axis(1,at=0.55,tick=FALSE,labels="CD8",cex.axis=1.3)
    axis(4,at=0.5,tick=FALSE,labels="PTEN",cex.axis=1.3,las=2,hadj=.6)
    axis(4,at=0.45,tick=FALSE,labels="(HGSOC)",cex.axis=1.2,las=2,hadj=.45)
```

```r
# general association between CD8 and PTEN for non-pathogenic (ie, not BRCA 1/2) mutation:
x = otta[otta$cancer_type=="HGSOC"&otta$mutation_type!="Pathogenic","cd84"]
y = otta[otta$cancer_type=="HGSOC"&otta$mutation_type!="Pathogenic","pten4_cytoplasm_r"]
mosaicplot(table(x,y),main="Only HGSOC cases without\npathogenic mutations",
       color = TRUE, shade = c(2,3,4,5),
       xlab = "",ylab="",las=2,dir="v",cex.axis=.725)
axis(1,at=0.55,tick=FALSE,labels="CD8",cex.axis=1.3)
axis(4,at=0.5,tick=FALSE,labels="PTEN",cex.axis=1.3,las=2,hadj=.6)
axis(4,at=0.45,tick=FALSE,labels="(HGSOC)",cex.axis=1.2,las=2,hadj=.45)
```

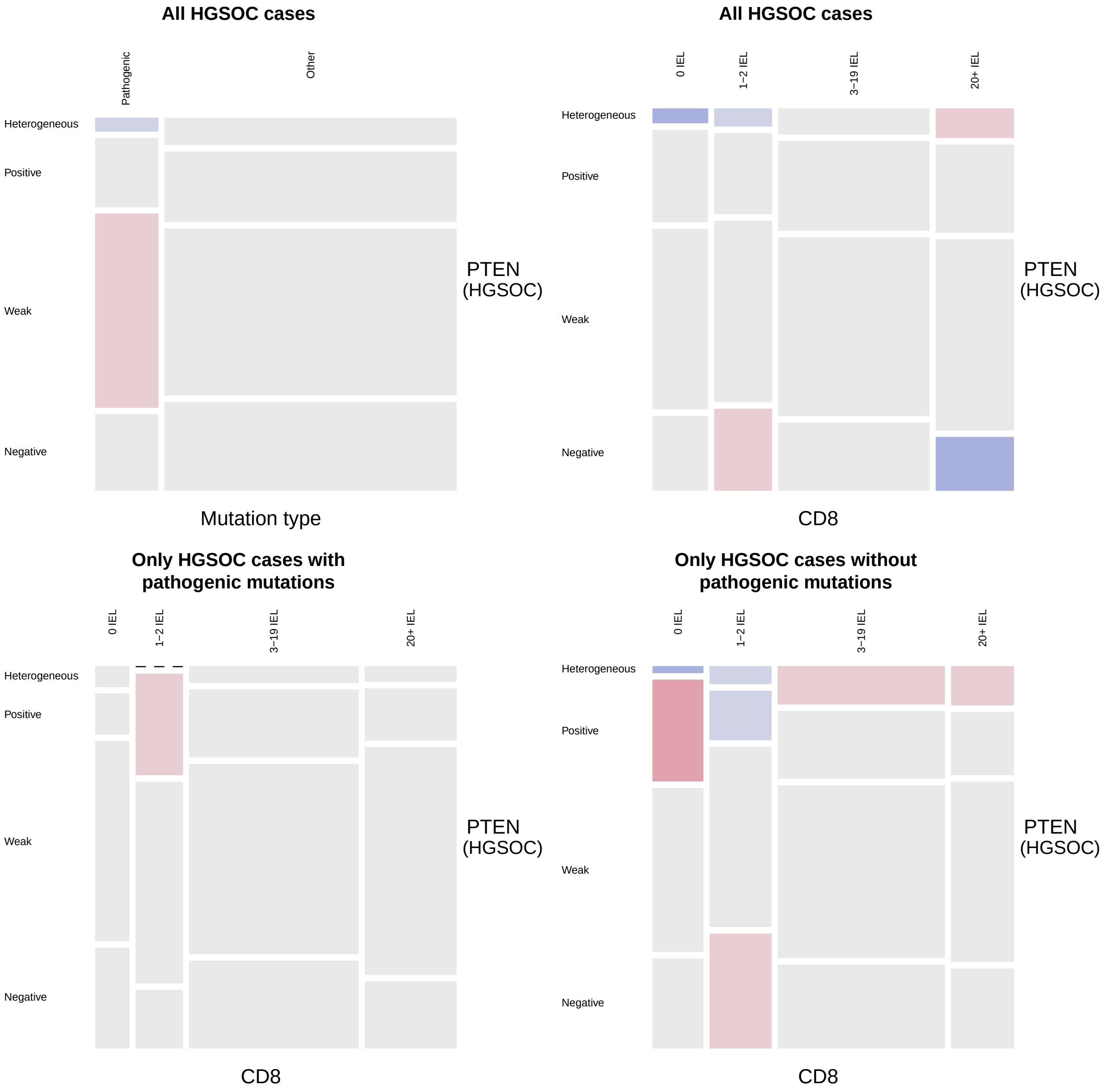

Generalised Cochran-Mantel-Haenszel test for the association between BRCA mutation status and PTEN:

```r
x = otta[otta$cancer_type=="HGSOC","mutation_type"]
y = otta[otta$cancer_type=="HGSOC","pten4_cytoplasm_r"]
```

```r
# not sig
CMHtest(table(x,y))
```

```
## Cochran-Mantel-Haenszel Statistics for x by y
##
##                  AltHypothesis   Chisq Df    Prob
## cor        Nonzero correlation 0.47397  1 0.49117
## rmeans  Row mean scores differ 0.47397  1 0.49117
## cmeans  Col mean scores differ 6.13004  3 0.10545
## general    General association 6.13004  3 0.10545
```

Generalised Cochran-Mantel-Haenszel test for the association between CD8 and PTEN:

```r
# general association between CD8 and PTEN for pathogenic (ie, BRCA 1/2) mutation:
x = otta[otta$cancer_type=="HGSOC","cd84"]
y = otta[otta$cancer_type=="HGSOC","pten4_cytoplasm_r"]
# not sig
CMHtest(table(x,y))
```

```
## Cochran-Mantel-Haenszel Statistics for x by y
##
##                  AltHypothesis  Chisq Df       Prob
## cor        Nonzero correlation 10.783  1 0.00102459
## rmeans  Row mean scores differ 13.493  3 0.00368274
## cmeans  Col mean scores differ 16.562  3 0.00086957
## general    General association 21.533  9 0.01048459
```

Generalised Cochran-Mantel-Haenszel test for the association between CD8 and PTEN for patients with pathogenic mutations:

```r
# general association between CD8 and PTEN for pathogenic (ie, BRCA 1/2) mutation:
x = otta[otta$cancer_type=="HGSOC"&otta$mutation_type=="Pathogenic","cd84"]
y = otta[otta$cancer_type=="HGSOC"&otta$mutation_type=="Pathogenic","pten4_cytoplasm_r"]
# not sig
CMHtest(table(x,y))
```

```
## Cochran-Mantel-Haenszel Statistics for x by y
##
##                  AltHypothesis    Chisq Df    Prob
## cor        Nonzero correlation 0.026486  1 0.87072
## rmeans  Row mean scores differ 0.593216  3 0.89798
## cmeans  Col mean scores differ 0.514294  3 0.91574
## general    General association 5.219111  9 0.81480
```

Generalised Cochran-Mantel-Haenszel test for the association between CD8 and PTEN for patients with non-pathogenic mutations:

```r
# general association between CD8 and PTEN for non-pathogenic (ie, not BRCA 1/2) mutation:
x = otta[otta$cancer_type=="HGSOC"&otta$mutation_type!="Pathogenic","cd84"]
y = otta[otta$cancer_type=="HGSOC"&otta$mutation_type!="Pathogenic","pten4_cytoplasm_r"]
# sig
CMHtest(table(x,y))
```

```
## Cochran-Mantel-Haenszel Statistics for x by y
##
##                  AltHypothesis  Chisq Df      Prob
## cor        Nonzero correlation  4.148  1 0.0416840
```

```
## rmeans  Row mean scores differ 10.178  3 0.0171140
## cmeans  Col mean scores differ 13.841  3 0.0031293
## general    General association 24.251  9 0.0039217
```

4 Figures

4.1 Association between PTEN and FIGO for HGSOC

Figure 2A of the article

```
x = otta[otta$cancer_type=="HGSOC","figo"]
y = otta[otta$cancer_type=="HGSOC","pten4_cytoplasm_r"]
par(mfrow=c(1,1),mar=c(2.5,0,0,4))
mosaicplot(table(x,y),main="",
       color = TRUE, shade = c(2,3,4,5),
       xlab = "",ylab="",las=2,dir="v",cex.axis=.725)
axis(1,at=0.55,tick=FALSE,labels="FIGO staging",cex.axis=1.3)
axis(4,at=0.5,tick=FALSE,labels="PTEN",cex.axis=1.3,las=2,hadj=.6)
axis(4,at=0.45,tick=FALSE,labels="(HGSOC)",cex.axis=1.2,las=2,hadj=.45)
```

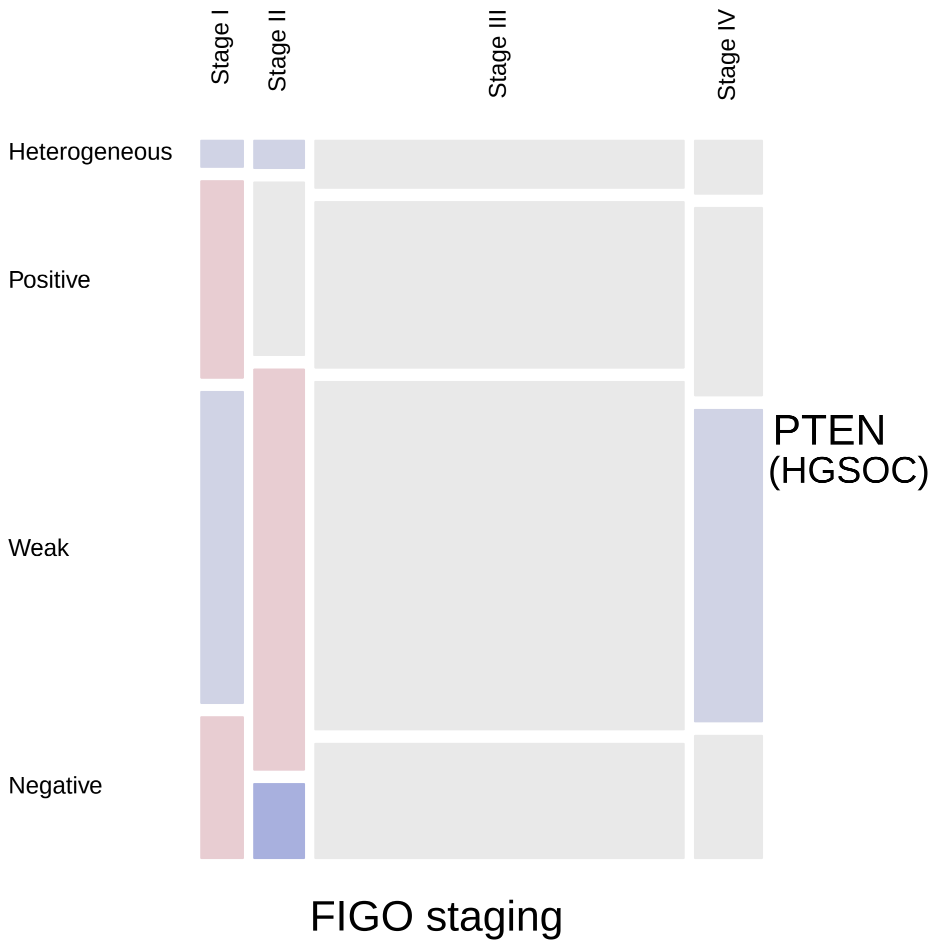

4.2 Association between CD8 and cancer-type

Figure 2C of the article

```
y = otta[,"cd84_r"]
x = otta[,"cancer_type"]
par(mfrow=c(1,1),mar=c(2.5,0,0,4))
mosaicplot(table(x,y),main="",
       color = TRUE, shade = c(2,3,4,5),
       xlab = "",ylab="",las=2,dir="v",cex.axis=.725)
axis(1,at=0.55,tick=FALSE,labels="Cancer Type",cex.axis=1.3)
axis(4,at=0.45,tick=FALSE,labels="CD8",cex.axis=1.3,las=2,hadj=.4)
```

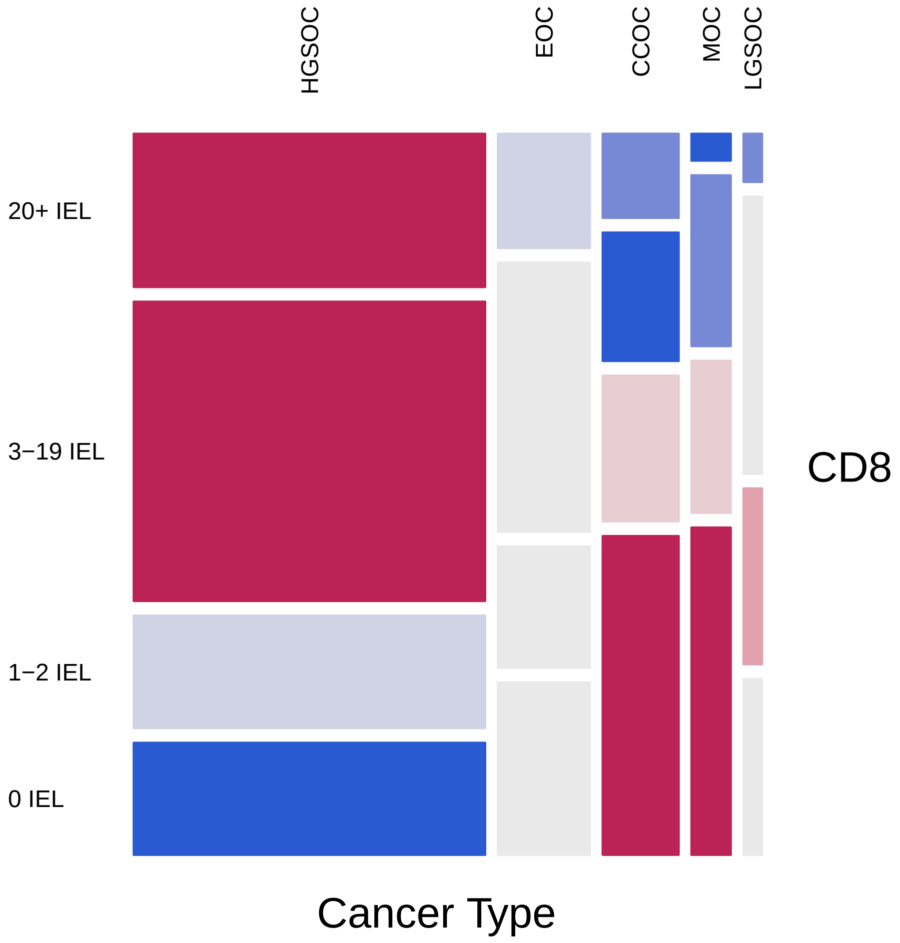

4.3 Association between PTEN and CD8 for CCOC

Figure 2D of the article

```
y = otta[otta$cancer_type=="CCOC","cd84_r"]
x = otta[otta$cancer_type=="CCOC","pten4_cytoplasm"]
```

```
par(mfrow=c(1,1),mar=c(2.5,0,0,4))
mosaicplot(table(x,y),main="",
       color = TRUE, shade = c(2,3,4,5),
       xlab = "",ylab="",las=2,dir="v",cex.axis=.725)
axis(1,at=0.55,tick=FALSE,labels="Cytoplasmic PTEN",cex.axis=1.3)
axis(4,at=0.45,tick=FALSE,labels="CD8",cex.axis=1.3,las=2,hadj=.4)
axis(4,at=0.40,tick=FALSE,labels="(CCOC)",cex.axis=1.2,las=2,hadj=.45)
```

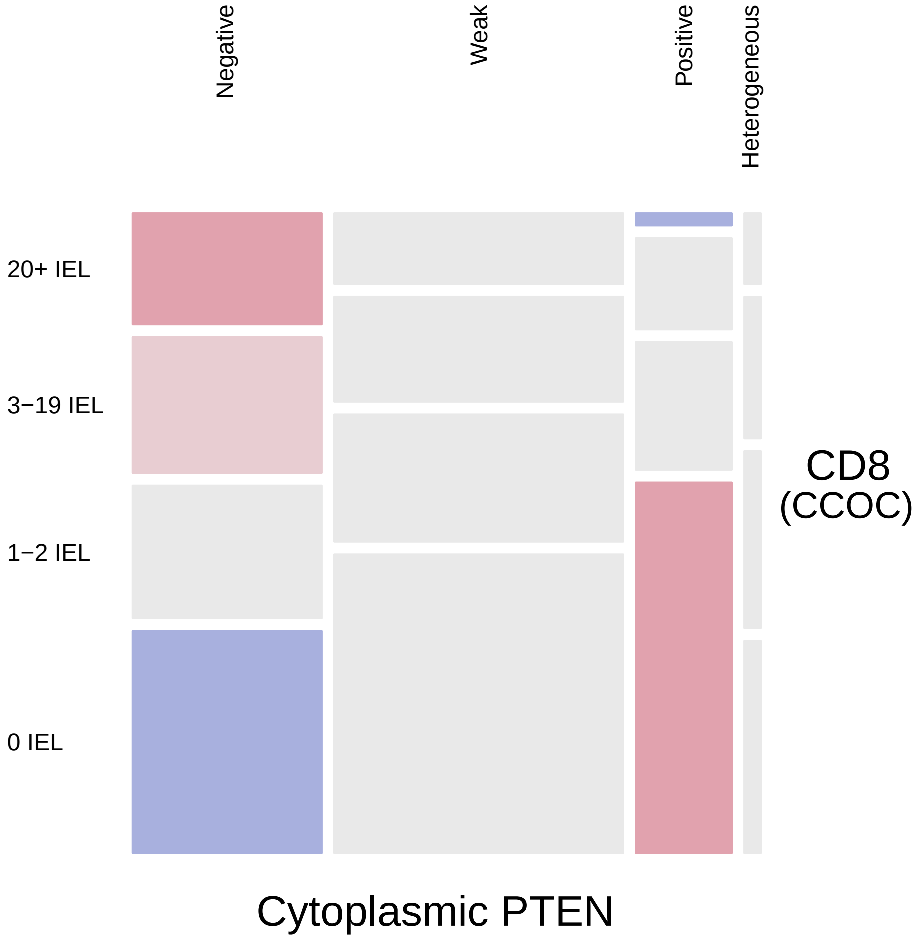

4.4 Association between PTEN and CD8 for HGSOC

Figure 2E of the article

```
y = otta[otta$cancer_type=="HGSOC","cd84_r"]
x = otta[otta$cancer_type=="HGSOC","pten4_cytoplasm"]
par(mfrow=c(1,1),mar=c(2.5,0,0,4))
mosaicplot(table(x,y),main="",
       color = TRUE, shade = c(2,3,4,5),
       xlab = "",ylab="",las=2,dir="v",cex.axis=.725)
axis(1,at=0.55,tick=FALSE,labels="Cytoplasmic PTEN",cex.axis=1.3)
axis(4,at=0.45,tick=FALSE,labels="CD8",cex.axis=1.3,las=2,hadj=.4)
axis(4,at=0.40,tick=FALSE,labels="(HGSOC)",cex.axis=1.2,las=2,hadj=.45)
```

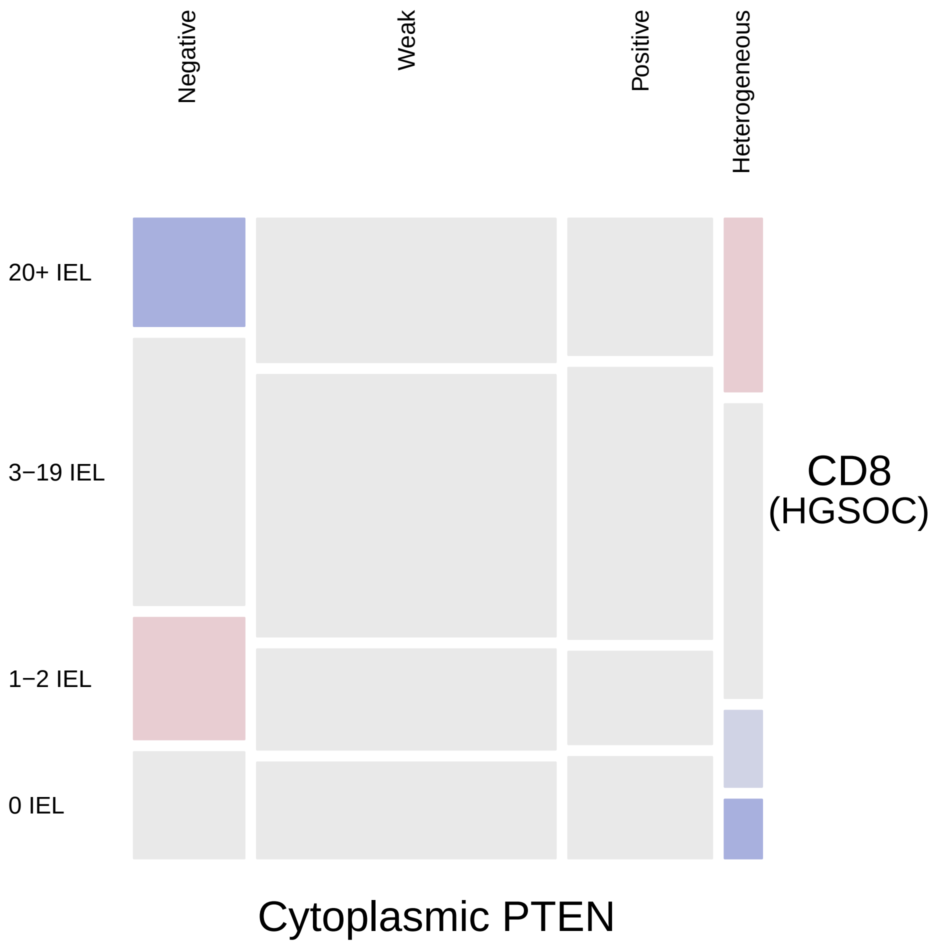

4.5 Association between Age group and PTEN for EOC

Figure 3A of the article

```
y = otta[otta$cancer_type=="EOC","pten4_cytoplasm_r"]
x = otta[otta$cancer_type=="EOC","agegroupatdiagnosis2"]
par(mfrow=c(1,1),mar=c(2.5,0,0,4))
mosaicplot(table(x,y),main="",
       color = TRUE, shade = c(2,3,4,5),
       xlab = "",ylab="",las=2,dir="v",cex.axis=.725)
axis(1,at=0.55,tick=FALSE,labels="Age group at diagnosis",cex.axis=1.3)
axis(4,at=0.50,tick=FALSE,labels="PTEN",cex.axis=1.3,las=2,hadj=.4)
axis(4,at=0.45,tick=FALSE,labels="(EOC)",cex.axis=1.2,las=2,hadj=.4)
```

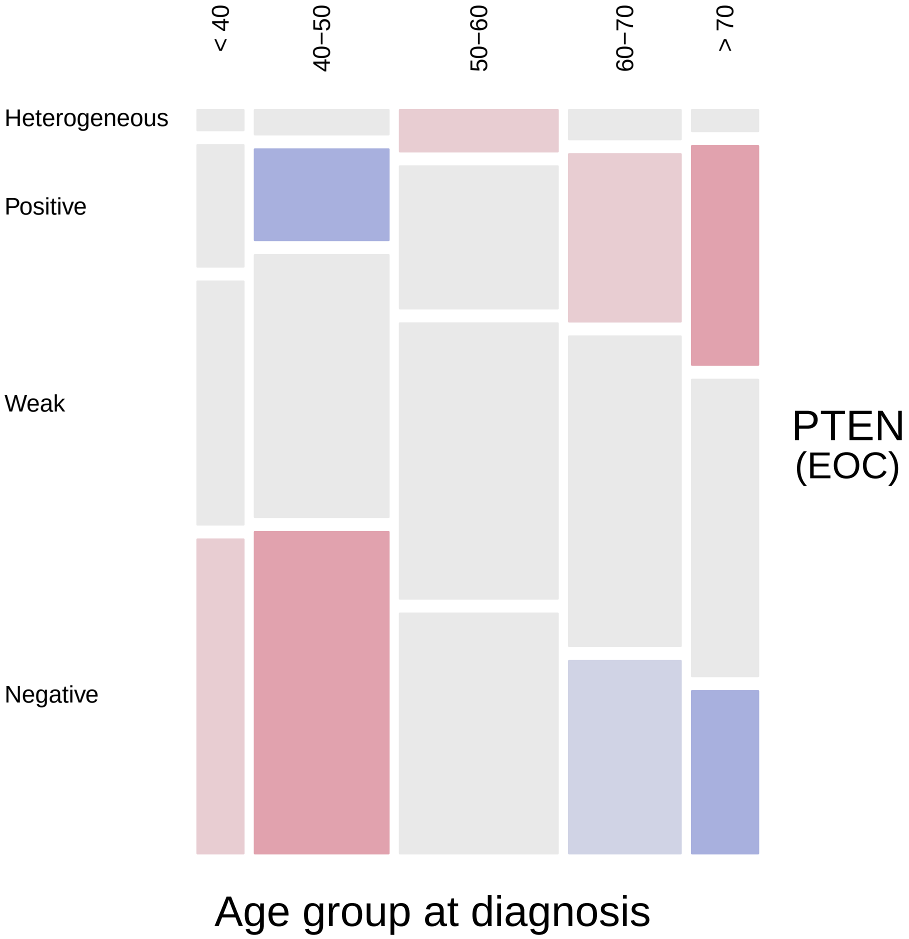

4.6 Association between PTEN and ER for HGSOC

Figure 3B of the article

```
y = otta[otta$cancer_type=="HGSOC","er3_r"]
x = otta[otta$cancer_type=="HGSOC","pten4_cytoplasm"]
par(mfrow=c(1,1),mar=c(2.5,0,0,4))
mosaicplot(table(x,y),main="",
       color = TRUE, shade = c(2,3,4,5),
       xlab = "",ylab="",las=2,dir="v",cex.axis=.725)
axis(1,at=0.55,tick=FALSE,labels="Cytoplasmic PTEN",cex.axis=1.3)
axis(4,at=0.45,tick=FALSE,labels="ER",cex.axis=1.3,las=2,hadj=.2)
axis(4,at=0.40,tick=FALSE,labels="(HGSOC)",cex.axis=1.2,las=2,hadj=.4)
```

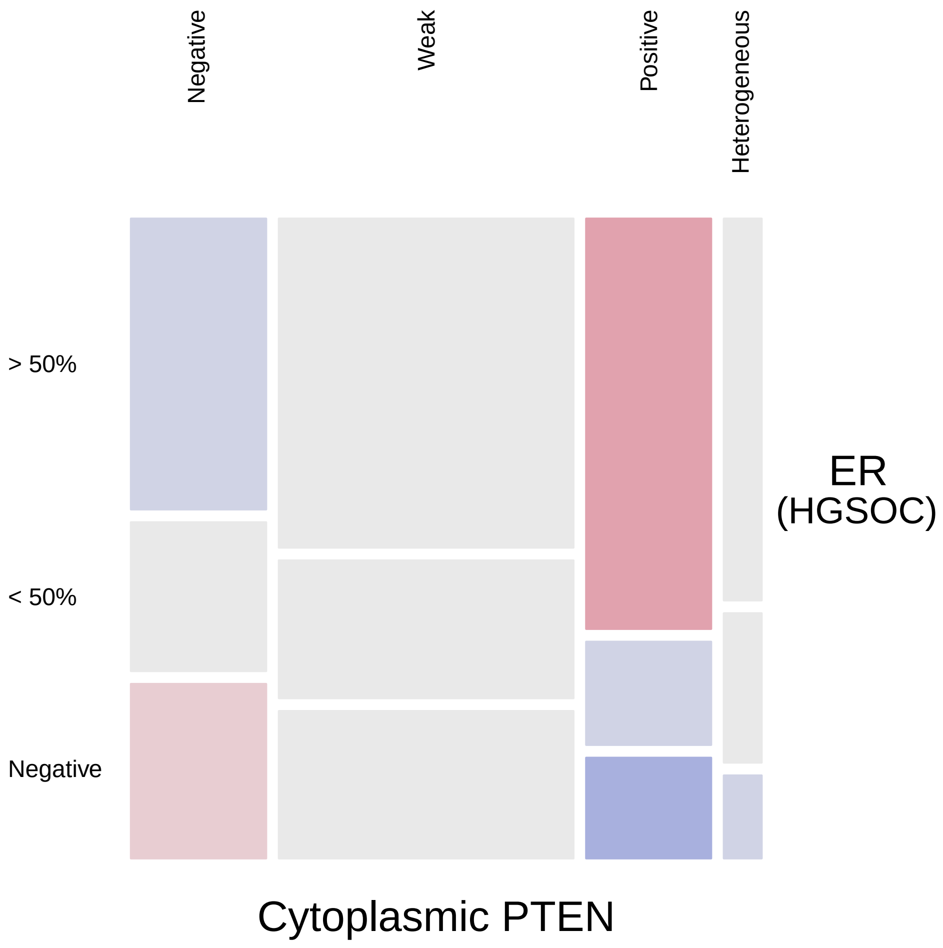

4.7 Association between PTEN and PR for HGSOC

Figure 3C of the article

```
y = otta[otta$cancer_type=="HGSOC","pr2_r"]
x = otta[otta$cancer_type=="HGSOC","pten4_cytoplasm"]
par(mfrow=c(1,1),mar=c(2.5,0,0,4))
mosaicplot(table(x,y),main="",
       color = TRUE, shade = c(2,3,4,5),
       xlab = "",ylab="",las=2,dir="v",cex.axis=.725)
axis(1,at=0.55,tick=FALSE,labels="Cytoplasmic PTEN",cex.axis=1.3)
axis(4,at=0.45,tick=FALSE,labels="PR",cex.axis=1.3,las=2,hadj=.2)
axis(4,at=0.40,tick=FALSE,labels="(HGSOC)",cex.axis=1.2,las=2,hadj=.4)
```

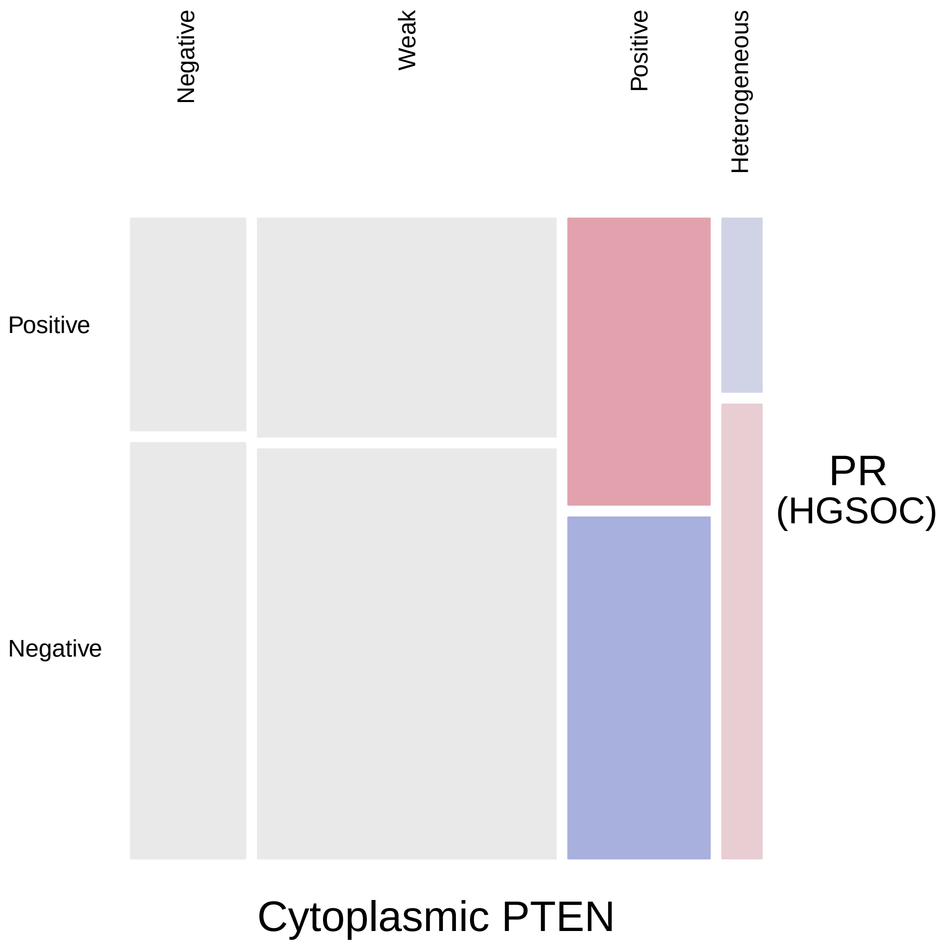

4.8 Association between PTEN and AR for HGSOC

Figure 3D of the article

```
y = otta[otta$cancer_type=="HGSOC","pr2_r"]
x = otta[otta$cancer_type=="HGSOC","pten4_cytoplasm"]
par(mfrow=c(1,1),mar=c(2.5,0,0,4))
mosaicplot(table(x,y),main="",
       color = TRUE, shade = c(2,3,4,5),
       xlab = "",ylab="",las=2,dir="v",cex.axis=.725)
axis(1,at=0.55,tick=FALSE,labels="Cytoplasmic PTEN",cex.axis=1.3)
axis(4,at=0.45,tick=FALSE,labels="PR",cex.axis=1.3,las=2,hadj=.2)
axis(4,at=0.40,tick=FALSE,labels="(HGSOC)",cex.axis=1.2,las=2,hadj=.4)
```

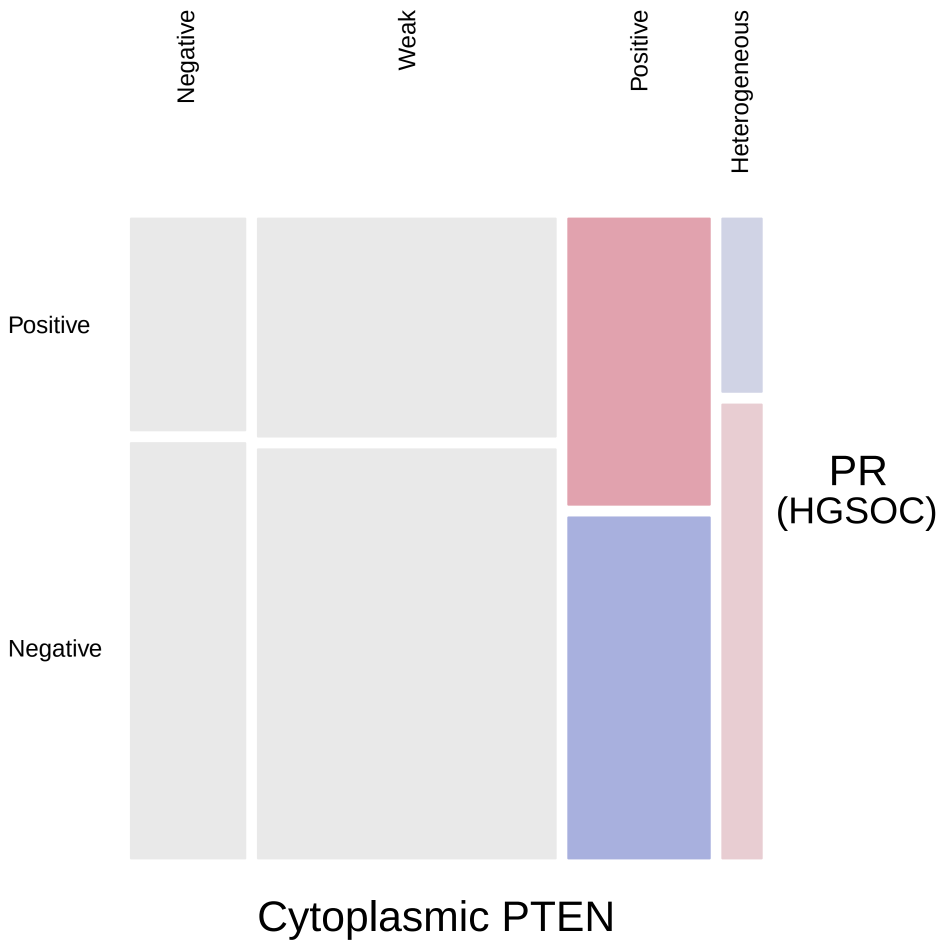
Negative
Weak
Positive
Heterogeneous
Positive
Negative
PR
(HGSOC)
Cytoplasmic PTEN

Script 4 - Survival

D.-L. Couturier / F. Martins / J. Brenton / P. Pharoah

Last modified: 13 Mar 2020

```
#   Input data files  :    results/rdata/otta-amended.rd
#   Output data files :    results/rdata/4-imp_m3d2_m=75_it=50.rd
#                          results/rdata/4-imp_m3d3_m=75_it=50.rd
#   Required R packages :  survival, mice, survminer, colorspace, multcomp
```

In this script, we first prepare the data for the planned survival analyses, propose some Kaplan-Meier figures, fit some

1 Preparation of the data

1.1 Left-truncated and right-censored survival outcome

1.1.1 Right censoring

We chose to censor survival times over 12.5 years (as information related to long follow-up is less reliable and as we are not interested in the instantaneous probability of death long time after diagnosis) as follows:

```
# upper limit
upper = 10
# time of last follow up (uc for upper censored)
otta$time.lastfollowup_year_uc = otta$time.lastfollowup/365.25
otta$time.lastfollowup_year_uc[otta$time.lastfollowup_year>upper] = upper
# time of interview (uc for upper censored)
otta$time.interview_year_uc = otta$time.interview/365.25
otta$time.interview_year_uc[otta$time.interview_year>upper] = upper
# status at last follow up (set any patient with lfu>10 as alive, ie right censored)
otta$status.lastfollowup_year_uc = otta$status.lastfollowup
otta$status.lastfollowup_year_uc[(otta$time.lastfollowup/365.25)>upper] = "Alive"
```

Consequently, the follow-up times of 820 (ie, ~ 16%) patients and the status at last follow-up of 111 patients (ie, ~ 2%) were amended.

1.1.2 Survival outcome

Here, we define the left-truncated and right-censored survival outcomes according to 3 definitions:

- definition 1: **time from diagnosis to death from any cause, left-truncated due to delayed patient enrolment and right-censored at 10 years** (This is the definition of Ovarian Tumor Tissue Analysis Consortium, 2017, JAMA Oncol., supplemental material, p2).
- definition 2 [**Selected definition for the article**]: **time from diagnosis to death from any cause except *other*, left-truncated due to delayed patient enrolment and right-censored at 10 years** (i.e., with this definition, suvival times of patients dead due to causes not related to the disease/treatment are considered as right-censored).
- definition 3: **time from diagnosis to death from any cause except *other*, left-truncated due to delayed patient enrolment and right-censored at 10 years, with unknown cause of death considered as missing** (i.e., with this definition, suvival times of patients dead due to causes not related to the disease/treatment are considered as right-censored and unknown cause of death are considered as missing).

```
# set events according to both definitions
def1 = otta$status.lastfollowup_year_uc!="Alive"
def2 = (otta$status.lastfollowup_year_uc!="Alive") &
       (otta$status.lastfollowup_year_uc!="Dead (other)")
def3 = def2
def3[otta$status.lastfollowup_year_uc=="Dead (unknown)"] = NA
table(def1,def2,useNA="always")
```

```
##        def2
## def1    FALSE TRUE <NA>
##   FALSE  2303    0    0
##   TRUE    167 2822    0
##   <NA>      0    0  108
```

```
table(def2,def3,useNA="always")
```

```
##        def3
## def2    FALSE TRUE <NA>
##   FALSE  2470    0    0
##   TRUE      0 1993  829
##   <NA>      0    0  108
```

```
#
n.def   = 3
id.def = data.frame(pos=1:n.def,id=c("AllDeath", "UnknownOrRelatedDeath", "RelatedToDeathOnly"))

# define according survival times
otta$survival.time_year_uc1  =
    Surv(time  = otta$time.interview_year_uc,
         time2 = otta$time.lastfollowup_year_uc,
         event = def1)
otta$survival.time_year_uc2  =
    Surv(time  = otta$time.interview_year_uc,
         time2 = otta$time.lastfollowup_year_uc,
         event = def2)
otta$survival.time_year_uc3  =
    Surv(time  = otta$time.interview_year_uc,
         time2 = otta$time.lastfollowup_year_uc,
         event = def3)
    # note: warnings correspond to missing date of interview or
    #       missing date of last follow-up
```

Note that

- *unknown causes of death* are considered as related to the disease for definition 2,
- the number of events strongly decreases with definition 3 due to a large proportion of cases with *unknown causes of death*
- one advantage of definitions 2 and 3 over definition 1 is that death due to unrelated causes (like car accidents and cardiac arrests, for example) does not attenuate the effect of predictors.

1.2 Main predictor

We define here **id.mainpred**, an R object identifying the main predictor (4-level Cytoplasmic PTEN) and change the contrats of this factor so that the reference corresponds to *positive*

```
# main predictor
temp        = c("pten4_cytoplasm")
n.mainpred  = length(temp)
id.mainpred = data.frame(pos=1:n.mainpred,id=temp,stringsAsFactors=FALSE,
                         name = c("Cytoplasmic PTEN"))
# change of contrasts
otta$pten4_cytoplasm = factor(as.character(otta$pten4_cytoplasm),
                              levels=c("Positive","Heterogeneous","Weak","Negative"))
```

1.3 List of secondary predictors (and transformations)

We define here the list of secondary predictors as well as the different models to be tested:

- model 1: no secondary predictor included in the model,
- model 2: similar model to the one of Ovarian Tumor Tissue Analysis Consortium, 2017, JAMA Oncol., p3 controlling for
  - age at diagnosis,
  - 4-level FIGO staging,
  - presence/absence of residual disease post treatment,
  - site,
- model 3: similar model to the one of Sieh et al, Lancet Oncology, 2013, p4
  - controlling for
    * age and age-squared at diagnosis,
    * 4-level FIGO staging,
    * presence/absence of residual disease post treatment,
    * 2-level grade (*Well/Moderate* versus *Poor/None*),
  - and stratified by site

- model 4:
  - controlling for
    * 4-level FIGO staging,
    * 2-level grade (*Well/Moderate* versus *Poor/None*),
    * site
  - and stratified by site *Age group* times *presence absence of residual disease*
- model 5 [**Selected model for the article**]: similar model to the one of Sieh et al, Lancet Oncology, 2013, p4
  - controlling for
    * age and age-squared at diagnosis,
    * 4-level FIGO staging,

        * presence/absence of residual disease post treatment,
    - and stratified by site

- model 6: similar model as model 5 but controlling for CD8
- model 7: similar model as model 5 but controlling for ER
- model 8: similar model as model 5 but controlling for AR
- model 9: similar model as model 5 but controlling for PR

Note that

- compared to Sieh et al, Lancet Oncology, 2013, p4, we didn't include pre-treatment CA125 levels in the model due to the high percentage of missing data,
- we did not use multivariable fractional polynomials for *age* here, as the R package *mfp* currently requires strictly right-censored observations and can't deal with left truncation.
- this choice of predictors **excludes** some sites of the analysis as missingness per variable is often very site-dependent. For example, the sites s002, s007, s014, s005 and s012 have no information about *residual tumour* and therefore are excluded of all analyses in this section.

```
# variable as included in the model
temp1    = c("figo","differentiation2",
            "tumourafter","strata(tumourafter)",
            "ageatdiagnosis","I(ageatdiagnosis^2)",
            "site","strata(site)",
            "strata(agegroupXtumourleft)",
            "cd82","er2","ar2","pr2")
# (same for rms which uses strat instead of strata)
temp2    = c("figo","differentiation2",
            "tumourafter","strat(tumourafter)",
            "ageatdiagnosis","I(ageatdiagnosis^2)",
            "site","strat(site)",
            "strat(agegroupXtumourleft)",
            "cd82","er2","ar2","pr2")
# corresponding variable
temp3   =  c("figo","differentiation2",
             "tumourafter","tumourafter",
             "ageatdiagnosis","ageatdiagnosis",
             "site","site",
             "agegroupXtumourleft",
             "cd82","er2","ar2","pr2")
n.secpred  = length(temp1)
id.secpred = data.frame(pos=1:n.secpred,id=temp1,var=temp3,rms=temp2,
                        stringsAsFactors=FALSE)
# 1st model: no secondary predictor
id.secpred$model1  = as.logical(c(0,0,0,0,0,0,0,0,0,0,0,0,0))
# 2nd model: jama 2017
id.secpred$model2  = as.logical(c(1,1,1,0,1,0,1,0,0,0,0,0,0))
# 3rd model: lancet 2013
id.secpred$model3  = as.logical(c(1,1,1,0,1,1,0,1,0,0,0,0,0))
# 4th model:
id.secpred$model4  = as.logical(c(1,1,0,0,0,0,1,0,1,0,0,0,0))
# 5th model: lancet 2013 without differentiation
id.secpred$model5  = as.logical(c(1,0,1,0,1,1,0,1,0,0,0,0,0))
# 6th model: lancet 2013+cd82
id.secpred$model6  = as.logical(c(1,0,1,0,1,1,0,1,0,1,0,0,0))
# 7th model: lancet 2013+er2
```

```
id.secpred$model7  = as.logical(c(1,0,1,0,1,1,0,1,0,0,1,0,0))
# 8th model: lancet 2013+ar2
id.secpred$model8  = as.logical(c(1,0,1,0,1,1,0,1,0,0,0,1,0))
# 9th model: lancet 2013+pr2
id.secpred$model9  = as.logical(c(1,0,1,0,1,1,0,1,0,0,0,0,1))
# 10th model: lancet 2013+cd82+er2+ar2+pr2
id.secpred$model9  = as.logical(c(1,0,1,0,1,1,0,1,0,1,1,1,1))
#
n.model  = ncol(id.secpred)-4
id.model = data.frame(pos=1:n.model,id=paste0("model",1:n.model),
                      stringsAsFactors=FALSE)
id.secpred[,c("id",id.model$id)]
```

```
# define agegroupXtumourleft
otta$agegroupXtumourleft = paste0(otta$agegroupatdiagnosis,otta$tumourafter)
otta$agegroupXtumourleft[is.na(otta$agegroupatdiagnosis)|is.na(otta$tumourafter)] = NA
otta$agegroupXtumourleft = factor(otta$agegroupXtumourleft)
```

2 Survival function estimates

For each cancer subtype, we estimate here the probability of survival as a function of time from diagnosis and levels of an ordinal variable by means of

- the Nelson-Altschuler-Aalen [NAA] estimator (solid line),
- the Kaplan-Meier [KM] product-limit estimator (dashed line).

The upper left plot coresponds to **Figure 2B of the article**.

```
n.cancer  = nlevels(otta$cancer_type)
id.cancer = data.frame(pos=1:n.cancer, id=levels(otta$cancer_type),
                       name=levels(otta$cancer_type2),stringsAsFactors=FALSE)

# chose definition [see above]
dw = 2
otta$survival.timew = otta[,paste0("survival.time_year_uc",dw)]
colw = c(
    rgb(6, 188, 241, maxColorValue = 255),
    rgb(100, 100, 100, maxColorValue = 255),
    rgb(235,7,142, maxColorValue = 255),
    rgb(55,56,149, maxColorValue = 255)
    )

# for each main predictor
for(pw in 1:n.mainpred){
    par(mfrow=c(2,3),omi=c(0,0,.4,0),mar=c(4,4,3,1))
    # for each cancer type
    for(cw in 1:n.cancer){
        data_cw = na.omit(otta[as.numeric(otta$cancer_type)==cw,
                              c("survival.timew",id.mainpred$id[pw])])
        fit_naa = survfit(as.formula(paste0("survival.timew ~",
                          id.mainpred$id[pw])),data = data_cw,
                          type="fleming-harrington")
```

```
    fit_km  = survfit(as.formula(paste0("survival.timew ~",
                      id.mainpred$id[pw])),data = data_cw,
                      type="kaplan-meier")
    levelw = levels(data_cw[,id.mainpred$id[pw]])
    n.levelw = length(levelw)
    plot(fit_naa, conf.int=FALSE, col=colw,
         xlab="",ylab ="Survival",axes=FALSE,
         xlim=c(0,upper),ylim=c(0,1),
         main=paste0(id.cancer$name[cw],"\n(n = ",nrow(data_cw),")"))
    lines(fit_km,col=colw,lty=2)
    axis(1,0:upper,pos=0)
    axis(1,5,"Time (years)",tick=FALSE,padj=1.5)
    axis(2,seq(0,1,.1),las=2,cex=.9)
    }
    # legend
    plot(1,1,pch="",axes=FALSE,xlab="",ylab="")
    legend("top",ncol=1,col=c(colw,NA,1,1),
           title = id.mainpred$name[pw],
           title.col = gray(0.25),
           lty=c(rep(1,n.levelw),NA,1,3),
           legend=c(levelw,"","NAA estimator","KM estimator"))
    # main title
    mtext(id.mainpred$name[pw],side=3,outer=3,cex=1.25,col=gray(0.25))
}
```

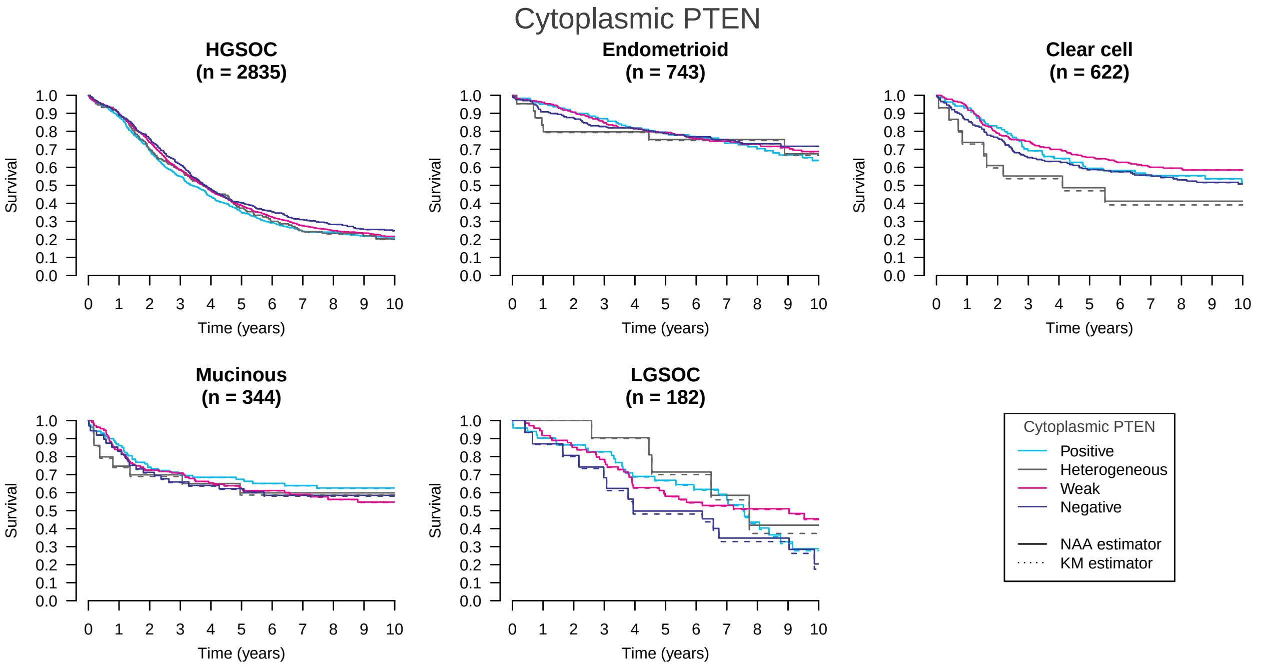

Note that

- the sample size per main predictor and cancer type is indicated in parenthesis. They do not correspond to the sample sizes reported in Table 1 of the manuscript due to missing data in the survival times (even for definitions 1 and 2 of the survival outcome).
- the NAA and KM estimators lead here to very similar survival probability estimates in all cases (as the risk set size never reaches 0).

3 Cox regressions: complete-case analyses

In the following code, for each cancer subtype (5), model (9) and definition (3), we store

- the Cox proportional hazard model parameter estimates,
- the sample size,
- the p-values of the proportional hazard test per variable/level as well as global,
- the p-value of the ANOVA when comparing the restricted (not including primary ooutcomes) and full models (including primary outomes)
- the concordance statistic as estimated by survConcordance() of the survival package.

3.1 Model fit

```
coxph_model_cancer_mainpred_def = list.fun(n.def,id.def$id)

# for each survival definition
for(dw in 1:n.def){
    otta$survival.timew = otta[,paste0("survival.time_year_uc",dw)]
    coxph_model_cancer_mainpred_def[[dw]] = list.fun(n.mainpred,id.mainpred$id)
    # for each main perdictor
    for(pw in 1:n.mainpred){
        coxph_model_cancer_mainpred_def[[dw]][[pw]] = list.fun(n.cancer,id.cancer$id)
        mainpredw = id.mainpred$id[pw]
        nlevelw   = nlevels(otta[,mainpredw])
        # for each cancer sub-type
        for(cw in 1:n.cancer){
            if(cw<5){
                coxph_model_cancer_mainpred_def[[dw]][[pw]][[cw]]=list.fun(n.model,id.model$id)
            }else{# special case for LGSOC due to small sample sizes
                coxph_model_cancer_mainpred_def[[dw]][[pw]][[cw]]=list.fun(1,id.model$id[1])
            }
            # for each model
            for(mw in 1:ifelse(cw<5,n.model,1)){

                ### PREPARE
                # formulas
                secpred_coxph = paste0(id.secpred$id[id.secpred[,id.model$id[mw]]],
                                       collapse=" + ")
                formula0 = as.formula(paste0("survival.timew ~ ",
                                      if(secpred_coxph!=""){paste0("+",secpred_coxph,collapse="")
                                      }else{1}))
                formula1 = as.formula(paste0("survival.timew ~ ",mainpredw,
                                      if(secpred_coxph!=""){paste0("+",secpred_coxph,collapse="")}))
                # data
                dataw = na.omit(otta[as.numeric(otta$cancer_type)==cw,
                                      c("survival.timew",mainpredw,
                                        id.secpred$var[id.secpred[,id.model$id[mw]]])])
                if(any(colnames(dataw)=="site")){
```

```
                    dataw$site = as.factor(as.character(dataw$site))
                    contrasts(dataw$site) = contr.sum(nlevels(dataw$site))
                    }

                ### FIT
                # coxph restricted and full models
                fit0 = coxph(formula=formula0,data=dataw)
                fit1 = coxph(formula=formula1,data=dataw)

                ## RESULTS
                # estimates and inference
                coefw   = coef(summary(fit1))[,,drop=FALSE]
                # global and per level/var ph test
                phtest  = try(cox.zph(fit1,terms=FALSE,transform="rank")[[1]],silent=TRUE)
                if(class(phtest)!="try-error"){
                    if(nrow(phtest)>1){
                        phtest1 = phtest[-nrow(phtest),"p"]
                        phtest2 = if(nrow(phtest)>1){phtest["GLOBAL","p"]}else{phtest1}
                    }else{
                        phtest1 = phtest2 = phtest[,"p"]
                    }
                }else{
                    phtest1 = phtest2 = NA
                }
                # wald test statistic
                wald    = anova(fit0,fit1)[2,"P(>|Chi|)"]
                # concordance
                conc    = summary(fit1)$concordance[1]
                # combine
                lmhpsn = data.frame(
                    low      = exp(coefw[,1]-qnorm(.975)*coefw[,3]),
                    mid      = exp(coefw[,1]),
                    high     = exp(coefw[,1]+qnorm(.975)*coefw[,3]),
                    p.value  = coefw[,5],
                    #sig      = sig.fun(coefw[,5]),
                    n        = nrow(dataw),
                    ph.test1 = phtest1,
                    ph.test2 = phtest2,
                    wald     = wald,
                    conc     = conc)
                # save
                coxph_model_cancer_mainpred_def[[dw]][[pw]][[cw]][[mw]] = lmhpsn
                }
            }
        }
    }
    # note: - warnings regarding site levels are expected
    #       - inference issue with complex models and small samples too
```

3.2 Selected results

Let's focus on the results of interest, ie, the ones related to cytoplasmic PTEN, (main predictor 1) and the HGSOC cancer subtype (cancer type 1):

```
# select main predictor and cancer type of interest:
pw = 1 # 1 for cytoplasmic PTEN
cw = 1 # 1 for HGSOC

# select results
colw        = colnames(coxph_model_cancer_mainpred_def[[dw]][[pw]][[cw]][[mw]])
ar.res.lidm = array(NA,dim=c(nlevels(otta$pten4_cytoplasm)-1,
                       length(colw),n.def,n.model),
                    dimnames=list(levels(otta$pten4_cytoplasm)[-1],colw,
                           id.def$id,id.model$id))
for(dw in 1:n.def){
    for(mw in 1:n.model){
        ar.res.lidm[,,dw,mw] = as.matrix(coxph_model_cancer_mainpred_def[[dw]][[pw]][[cw]][[mw]][1:nleve
    }
}
# plot HR, CI and p-val
par(mfrow=c(1,1),mar=c(3,4,2,1))
ylim1 = c(0,round(max(ar.res.lidm[,"p.value",,]*10))/10)
ylim2 = c(round(min(ar.res.lidm[,"low",,]*10))/10,
          round(max(ar.res.lidm[,"high",,]*10))/10)
ylimw = c(-.1,ylim2[2])
xlimw = c(.5,n.def*(dim(ar.res.lidm)[1]+1)-.5)
temp  = matrix(1:(n.def*(dim(ar.res.lidm)[1]+1)),ncol=dim(ar.res.lidm)[1])
plot(1,1,pch="",xlab="PTEN level, Definitions and Models",ylab="",
     xlim=xlimw,ylim=ylimw,axes=FALSE)
abline(h=seq(0,1.7,.1),col="light gray",lty=3)
abline(h=c(0,1),col="gray",lwd=2)
abline(h=c(0.05),col="gray",lwd=1,lty=1)
# arrows and pval
for(dw in 1:n.def){
    for(lw in 1:dim(ar.res.lidm)[1]){
        for(mw in 1:n.model){
            lmhp = ar.res.lidm[lw,c("low","mid","high","p.value"),dw,mw]
            pos = (dim(ar.res.lidm)[1]+1)*(lw-1)+dw+c(seq(-.15,.15,length=n.model))[mw]
            arrows(pos,lmhp[1],pos,lmhp[3],col=col1.fun(n.model)[mw],code=3,angle=90,length=.02)
            points(pos,lmhp[2],col=col1.fun(n.model)[mw],pch=1,cex=.5)
            points(pos,lmhp[4],col="red",pch=1,cex=.5)
            }
        }
    }
# axes
axis(2,seq(ylim1[1],ylim1[2],.1),col.axis="red",col="red",las=2)
axis(2,mean(ylim1),"p-value",
     col.axis="red",col="red",tick=FALSE,padj=-3)
axis(2,seq(ylim2[1],ylim2[2],.1),col.axis="black",col="black",las=2)
axis(2,mean(ylim2),"Hazard ratio",
     col.axis="black",col="black",tick=FALSE,padj=-3)
axis(1,temp[-nrow(temp),],rep(paste0("Def\n",1:n.def),dim(ar.res.lidm)[1]),
     cex.axis=.75,tick=FALSE,col.axis="blue")
```

```
axis(3,apply(temp[-nrow(temp),],2,mean),levels(otta[,id.mainpred$id[pw]])[-1],
     cex.axis=.75,tick=FALSE,col.axis="blue")
# legend
legend("bottom",col=col1.fun(n.model),lty=1,lwd=2,legend=id.model$id,
       ncol=n.model,box.lwd=NA,cex=.75)
```

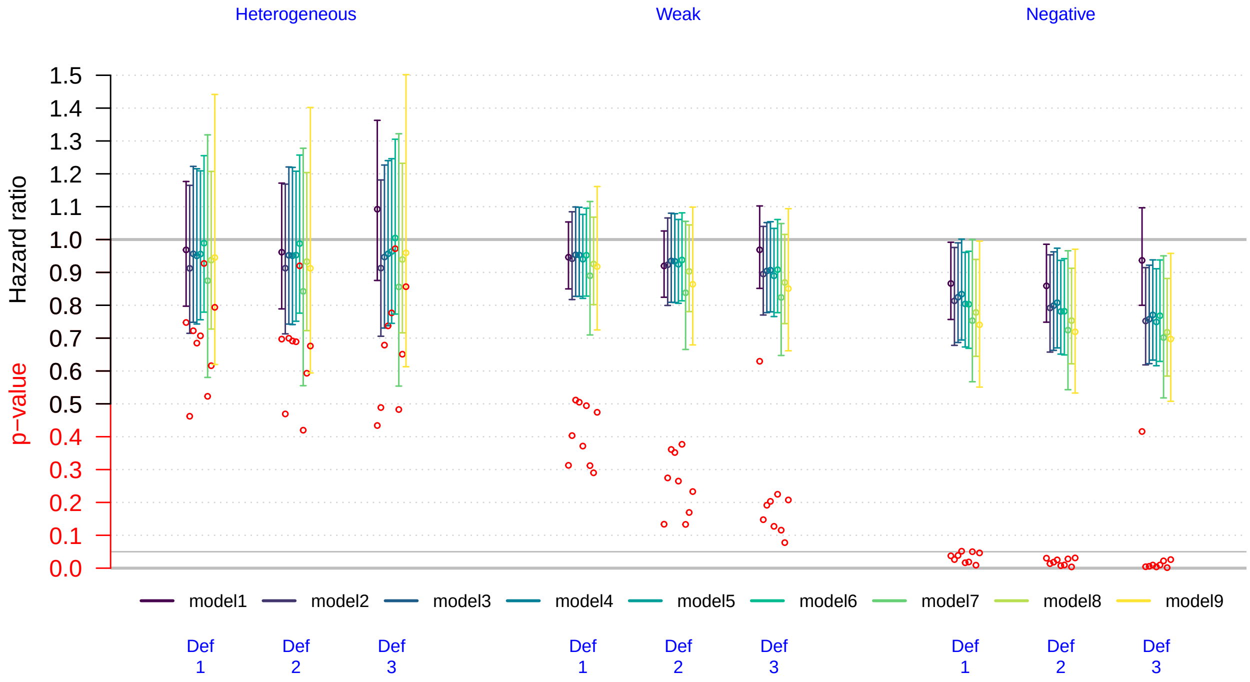

3.3 Table S4

```
# model per cancer type
id.cancer$model = rep(c("model5","model1"),c(4,1))
# fit
TableS4 = NULL
for(cw in 1:n.cancer){
    ### PREPARE
    # formulas
    secpred_coxph = paste0(id.secpred$id[id.secpred[,id.cancer$model[cw]]],
                           collapse=" + ")
    formula1 = as.formula(paste0("survival.time_year_uc2 ~ pten4_cytoplasm",
                          if(secpred_coxph!=""){paste0("+",secpred_coxph,collapse="")}))
    # data
    dataw = na.omit(otta[as.numeric(otta$cancer_type)==cw,
                          c("survival.time_year_uc2","pten4_cytoplasm",
                            id.secpred$var[id.secpred[,id.cancer$model[cw]]])])
    if(any(colnames(dataw)=="site")){
        dataw$site = as.factor(as.character(dataw$site))
        contrasts(dataw$site) = contr.sum(nlevels(dataw$site))
        }

    ### FIT
```

```
    fit1 = coxph(formula=formula1,data=dataw)

    ## RESULTS
    coefw   = coef(summary(fit1))[,,drop=FALSE]
    # combine
    lmhpsn = data.frame(
        low      = exp(coefw[,1]-qnorm(.975)*coefw[,3]),
        mid      = exp(coefw[,1]),
        high     = exp(coefw[,1]+qnorm(.975)*coefw[,3]),
        p.value  = pval.fun(coefw[,5]),
        sig      = sig.fun(coefw[,5]))[1:3,]
    lmhpsn = data.frame(Cancer = id.cancer$id[cw],
                        Level = levels(dataw$pten4_cytoplasm)[-1],
                        lmhpsn)
    # multiplicity correction:
    pval.adj = summary(glht(fit1, linfct = mcp(pten4_cytoplasm = "Dunnet")))[[10]]$pvalues
    lmhpsn$p.value = pval.fun(pval.adj)
    lmhpsn$sig  = sig.fun(pval.adj)
    lmhpsn$low  = format(round(lmhpsn$low,3))
    lmhpsn$mid  = format(round(lmhpsn$mid,3))
    lmhpsn$high = format(round(lmhpsn$high,3))
    # save
    TableS4 = rbind(TableS4,lmhpsn)
    }
# print
TableS4
```

3.4 Model checks

3.4.1 Proportional hazard assumption

We focus here on the p-values of the proportional hazard test based on the scaled Schoenfeld residuals.

```
par(mfrow=c(1,1),mar=c(5,5,2,5))
ylimw = c(0,1.1)#round(max(ar.res.lidm[,"ph.test2",,]*10))/10)
xlimw = c(.5,n.def+.5)
plot(1,1,pch="",xlab="",ylab="",
     xlim=xlimw,ylim=ylimw,axes=FALSE)
abline(h=seq(0,1,.1),col="light gray",lty=3)
abline(h=c(0),col="gray",lwd=2)
abline(h=c(0.05),col="gray",lwd=1,lty=1)
# pval
for(dw in 1:n.def){
        for(mw in 1:n.model){
            pc = ar.res.lidm[1,c("ph.test2","conc"),dw,mw]
            p3 = ar.res.lidm[1:3,c("ph.test1"),dw,mw]
            pos  = dw+seq(-.25,.25,length=n.model)[mw]
            points(pos,pc[1],col="red",pch=1,cex=1)
            points(pos,pc[2],col="violet",pch=3,cex=1)
```

```r
            points(rep(pos,3),p3,col="blue",pch=4,cex=.5)
        }
    }
#
axis(1,rep(1:n.def,each=n.model)+rep(seq(-.25,.25,length=n.model),n.def),
     rep(paste0("model ",1:n.model),n.def),las=2,col.axis="blue")
axis(3,1:n.def,
     paste0("Definition ",1:n.def),las=1,col.axis="blue",tick = FALSE)
axis(2,seq(0,1,.1),las=2)
# legend
legend("top",ncol=3,legend=c("Global PH test p-value","Concordance value",
       "PTEN parameter PH test p-value"),cex=.75,
       bg="white",col=c("red","violet","blue"),pch=c(1,3,4))
```

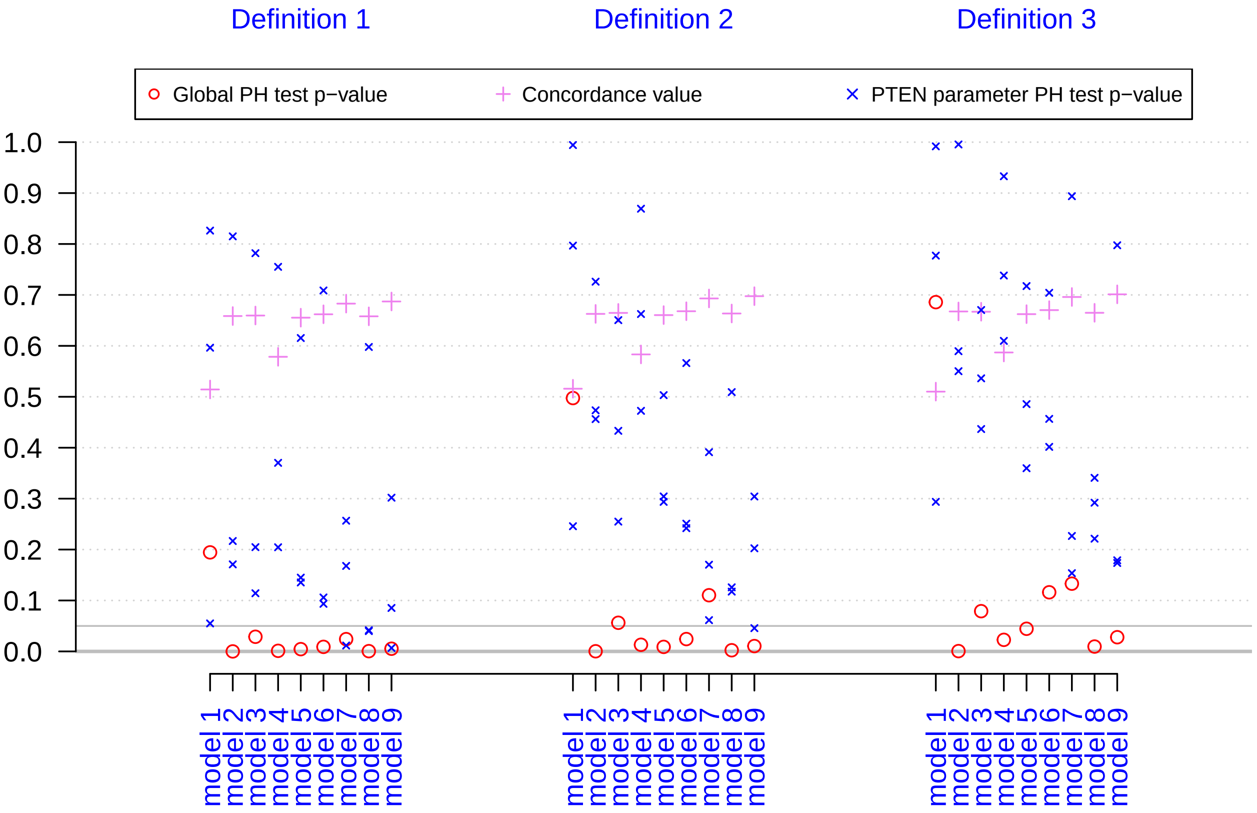

3.4.2 Influential observations

We have a quick look here at the deviance residuals for one of the most important models:

```r
pw = 1 # 1 = cytoplasmic PTEN
dw = 2 # 2 = selected definiation
cw = 1 # 1 = HCSOC
mw = 5 # 3 = model

# prepare (as above)
otta$survival.timew = otta[,paste0("survival.time_year_uc",dw)]
mainpredw = id.mainpred$id[pw]
```

```
nlevelw   = nlevels(otta[,mainpredw])
secpred_coxph = paste0(id.secpred$id[id.secpred[,id.model$id[mw]]],
                        collapse=" + ")
formula1 = as.formula(paste0("survival.timew ~ ",mainpredw,
                       if(secpred_coxph!=""){paste0("+",secpred_coxph,collapse="")}))
dataw = na.omit(otta[as.numeric(otta$cancer_type)==cw,
                       c("survival.timew",mainpredw,
                         id.secpred$var[id.secpred[,id.model$id[mw]]])])
if(any(colnames(dataw)=="site")){
    dataw$site = as.factor(as.character(dataw$site))
    contrasts(dataw$site) = contr.sum(nlevels(dataw$site))
    }
# fit
fit1 = coxph(formula=formula1,data=dataw)
# deviance residual plot
ggcoxdiagnostics(fit1, type = "deviance",
                 linear.predictions = FALSE,
                 ggtheme = theme_bw(),ylim=c(-3,3),
                 point.col=rainbow(nlevels(dataw$site))[as.numeric(dataw$site)])
```

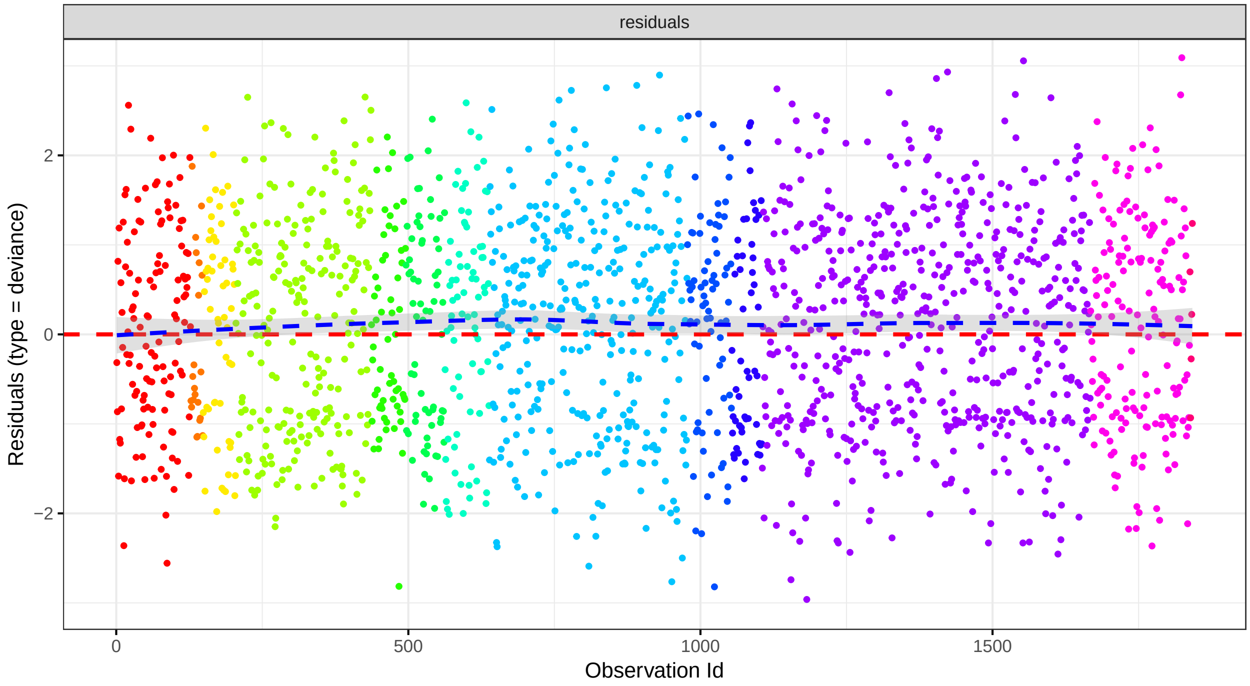

There do not seem to be obvious outliers according to this model check. The following residual plots, based on Schoenfeld residuals, suggests that some observations may be influential ones. When removing them, the same main predictor parameter estimates were obtained.

```
par(mfrow=c(3,3))
for(pw in 1:9){
plot(cox.zph(fit1,terms=FALSE,transform="rank")[pw],col="red");abline(h=0,col="blue")
}
```

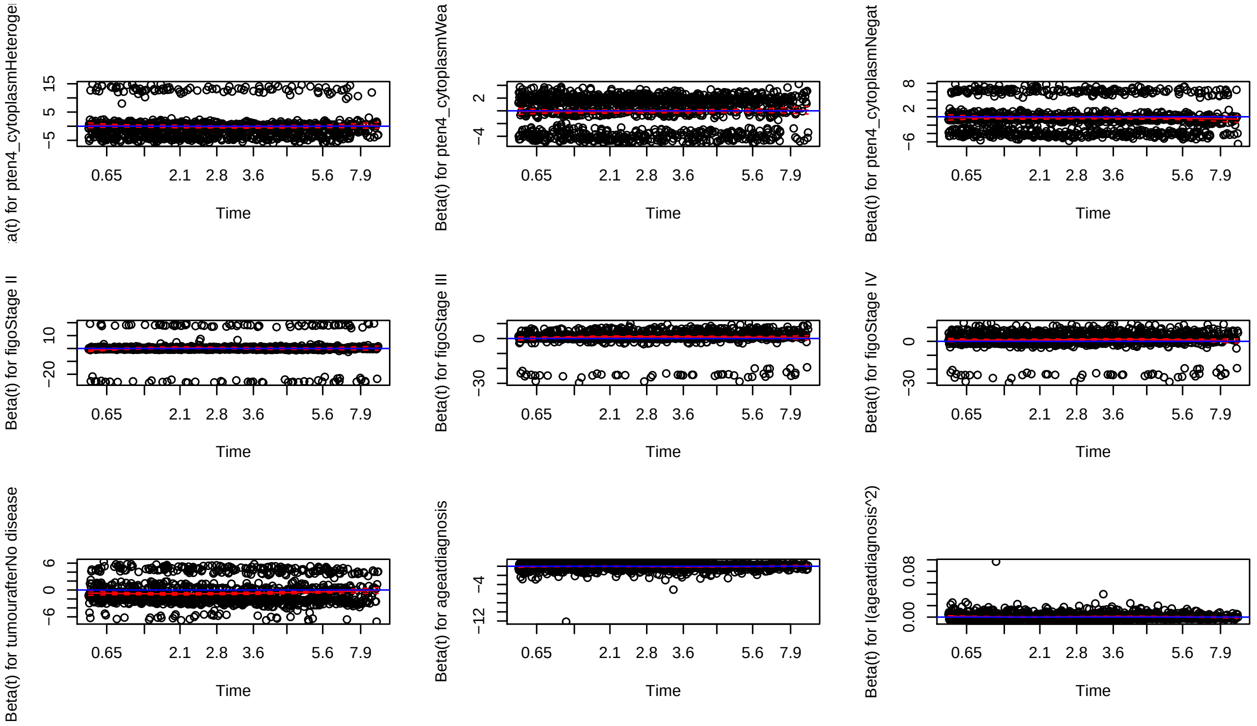

4 Cox regressions with multiple imputations of the missing predictors

In the previous section, we considered complete-case analyses. In this section, we impute the missing data of **survival predictors** (ie, we do not consider imputation of missing survival outcomes, defined in our case by the interview and last-follow-up times as well as the event status indicator) by means of multiple imputation chained equations (MICE) for the following case **stratified by cancer-type**:

- model 5 + survival outcome definition 2.

As the missing data pattern is strongly site-dependent, with some sites showing 100% missing data for some predictors, the imputation **can not be stratified by site**, as model 3 and 5 would ideally require.

In the right censored case, when imputing predictors of survival, it is common to use *survival time* and *event status* OR the *cumulative hazard* and the *event status* (White and Royston, 2009) as predictors of the explanatory variables showing missingness. Van Buuren (2012, section 7.1.8) notes that *survival time* and estimated *cumulative hazard* have a very strong correlation (0.997 in one of his example) so that results should not change much. Also, in the same section, he suggests choosing the scale of predictors in the imputation model based on the (observed pairwise-complete) correlation with variables showing missingness.

4.1 Imputation (stratified per cancer subtype)

In this section, we define our imputation model based on estimates of the correlation matrix between the variables of interest and prepare the data accordingly, proceed to the imputation and save the results.

4.1.1 Imputation model

Due to the left truncation, we considered variants of *survival times from diagnosis* and *survival times from interview* as predictors for the missing data.

```
# predictor list of model 5
temp       = c(id.mainpred$id,unique(id.secpred[id.secpred$model5,"var"]),
               "ageatdiagnosis2")
temp[temp=="ar2"] = "ar4"
n.imppred  = length(temp)
id.imppred = data.frame(pos=1:n.imppred,id=temp,stringsAsFactors=FALSE)
# create age squared as it is in the model
otta$ageatdiagnosis2 = otta$ageatdiagnosis^2
# create log of the treatment response ratio (refer to importation script)
otta$logtreatmentresponse = log(otta$treatmentresponse_ratio)
# temporary dataset
temp              = otta[,c(id.imppred$id,
                          "mutation_type","cancer.mother","priorcancer","priorrelatedcancer","logtreatm
temp$survtime         = otta$time.lastfollowup/365.25
temp$logsurvtime      = log(temp$survtime+2)
temp$sqrtsurvtime     = sqrt(temp$survtime)
temp$survtimerisk     = otta$time.lastfollowup/365.25-otta$time.interview/365.25
temp$logsurvtimerisk  = log(temp$survtimerisk+2)
temp$sqrtsurvtimerisk = sqrt(temp$survtimerisk)
temp$event            = otta$survival.time_year_uc2[,3]
temp$cumhazard        = nelsonaalen(temp, survtime, event)
temp$cumhazardrisk    = nelsonaalen(temp, survtimerisk, event)
temp$pten4_cytoplasm  = as.numeric(otta$pten4_cytoplasm)
temp$ar4              = c(1,2,2,3)[as.numeric(otta$ar4)]
for(cw in 1:ncol(temp)){temp[,cw] = as.numeric(temp[,cw])}
# crude correlation per cancer type
cumscore = NULL
for(cw in 1:nlevels(otta$cancer_type)){
    cor_sperman = cor(temp[as.numeric(otta$cancer_type)==cw,],
                  use="pairwise.complete.obs",method="spearman")
    cor_pearson = cor(temp[as.numeric(otta$cancer_type)==cw,],
                  use="pairwise.complete.obs",method="pearson")
    cor_pearson = cor_pearson[apply(!is.na(cor_pearson),2,any),apply(!is.na(cor_pearson),2,any)]
    heatmap(cor_pearson, col=col3.fun(50), symm=TRUE,cex.axis=.75,margin=c(10,10),
            ylab=levels(otta$cancer_type)[cw])
    posw = which(!is.na(match(colnames(cor_pearson),c(c("survtime","survtimerisk",
                 "sqrtsurvtime","sqrtsurvtimerisk",
                 "logsurvtime","logsurvtimerisk",
                 "cumhazard","cumhazardrisk","event")))))
    cumscore = rbind(cumscore,apply(cor_pearson[-(posw),posw],2,function(x)sum(abs(x))))

}
```

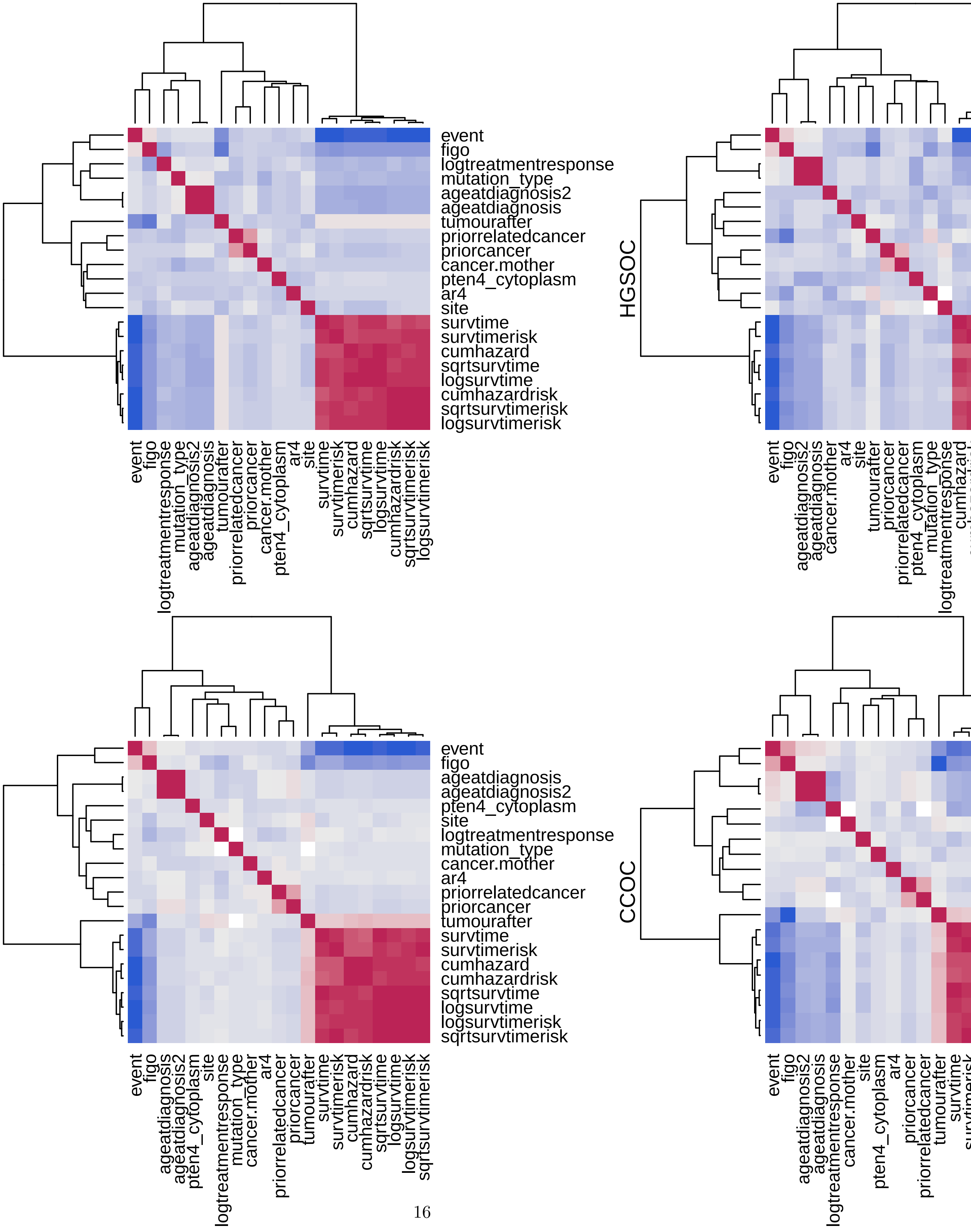
event
figo
logtreatmentresponse
mutation_type
ageatdiagnosis2
ageatdiagnosis
tumourafter
priorrelatedcancer
priorcancer
cancer.mother
pten4_cytoplasm
ar4
site
survtime
survtimerisk
cumhazard
sqrtsurvtime
logsurvtime
cumhazardrisk
sqrtsurvtimerisk
logsurvtimerisk
HGSOC
event
figo
ageatdiagnosis
ageatdiagnosis2
pten4_cytoplasm
site
logtreatmentresponse
mutation_type
cancer.mother
ar4
priorrelatedcancer
priorcancer
tumourafter
survtime
survtimerisk
cumhazard
cumhazardrisk
sqrtsurvtime
logsurvtime
logsurvtimerisk
sqrtsurvtimerisk
CCOC

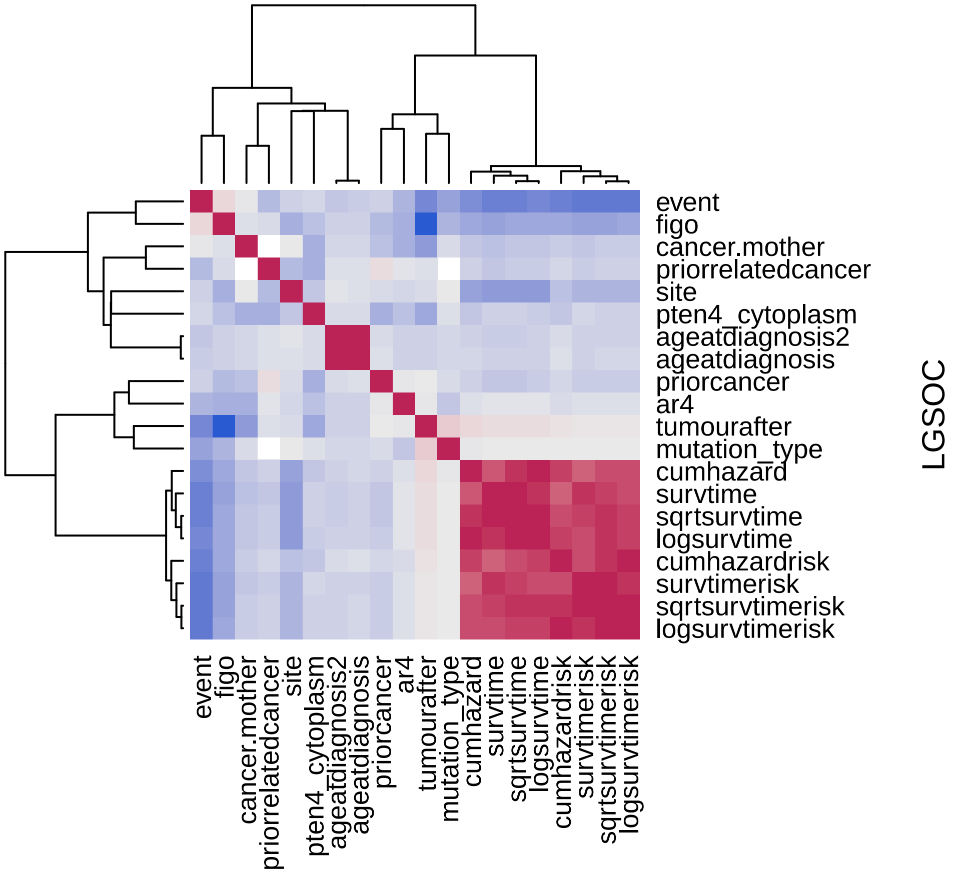


```r
cumscore = rbind(cumscore,apply(cumscore,2,sum))
rownames(cumscore) = c(levels(otta$cancer_type),"total")
as.data.frame(cumscore)
```

The last table shows the cumulative absolute Pearson correlation of 9 variables related to survival to their predictors (Results using Spearman are fairly similar). The results suggest that using the **log of the (shifted) total survival time** (or the square root of the total survival time) may be best to predict the variables of interest in our case. **All options are likely to lead to very similar results though** given the strong correlations observed above.

4.1.2 Preparation of the variables

Here, we adapt the class (continuous, categorical, ordinal) of the variables (plays a key role in the imputation).

```r
# modify class when required
sapply(otta[,id.imppred$id],class)
```

```
## pten4_cytoplasm            figo     tumourafter   ageatdiagnosis
##        "factor"        "factor"        "factor"        "numeric"
##            site ageatdiagnosis2
##        "factor"       "numeric"
```

```r
otta$cd84 = ordered(otta$cd84)
otta$pten4_nucleus = ordered(otta$pten4_nucleus)
otta$pr3  = ordered(otta$pr3)
otta$er3  = ordered(otta$er3)
otta$figo = ordered(otta$figo)
```

```
otta$logsurvtime = temp$logsurvtime
```

4.1.3 Model 5 + survival outcome definition 2

Here we impute the predictors of model 2 on the set of patients with available survival outcomes under definition 2 and store the results under results/rdata/4-imp_m3d2_m=25_it=25.rd (75 samples and 50 iterations for each cancer type). Running this code requires some time (a night).

```
if(!any(dir("results/rdata/")=="4-imp_m3d2_m=75_it=50_C.rd")){
    # survival outcome definition
    dw = 2
    otta$survival.timew = otta[,paste0("survival.time_year_uc",dw)]
    # required for post imputation estimation
    otta$event        = otta$survival.timew[,3]
    otta$time0        = otta$survival.timew[,1]
    otta$time1        = otta$survival.timew[,2]
    # per cancer-type stratification
    otta_cancertype = split(otta[!is.na(otta$survival.timew),
                                  c("time0","time1","logsurvtime","event",id.imppred$id,
                                    "mutation_type","cancer.mother","priorcancer",
                                    "priorrelatedcancer","logtreatmentresponse")],
                             otta[!is.na(otta$survival.timew),"cancer_type"])
    # initialise
    ini = mice(otta_cancertype[[1]], maxit=0, pri=FALSE, vis="monotone")
    # predictor
    pred = ini$pred
    pred[,c("ageatdiagnosis2","time0","time1")] = 0
    # method
    meth <- ini$meth
    meth[c("ageatdiagnosis","ageatdiagnosis2","logtreatmentresponse")] = c("pmm", "~I(ageatdiagnosis^2)
    # visiting scheme
    vis <- ini$vis

    #
    set.seed(30)
    imp_cancertype = list.fun(n.cancer,id.cancer$id)
    for(cw in 1:1){
        imp_cancertype[[cw]] = mice(otta_cancertype[[cw]], meth = meth,
                                    pred = pred,m=75,maxit=50)
        }
    save(dw,imp_cancertype,otta_cancertype,file="results/rdata/4-imp_m3d2_m=75_it=50_C.rd")
}
```

4.2 Combination of estimates and pooled parameter estimates

In this section, we combine the fits based on the imputed samples and define the final fit.

4.2.1 Model 5 + survival outcome definition 2

Here we focus on the main results of interest, ie, the effect of PTEN (pw=1) on survival of patients with HGSOC (cw=1) using model 3

```
# load imputed samples
print(dir("results/rdata/"))
```

```
## [1] "1-otta-amended.rd"          "1-otta-raw.rd"
## [3] "1-raters.rd"                "2-boot.rd"
## [5] "4-imp_m3d2_m=75_it=50_C.rd"
```

```
if(any(dir("results/rdata/")=="4-imp_m3d2_m=75_it=50_C.rd")){
    load("results/rdata/4-imp_m3d2_m=75_it=50_C.rd")
    }
# select case of interest
cw = 1  # HGSOC:  (for other cancer type, you may need to remove some predictors
        #          like 'logtreatmentresponse' for LGSOC as there was only one non-missing observation)
pw = 1  # PTEN:   do not change
dw = 2  # Def2:   do not change
mw = 5  # Model5: do not change

###
### COMBINE FITS
### model has to be typed (as.formula(), eval() not working in this context)
###
fit <- with(imp_cancertype[[cw]],
           coxph(Surv(time0, time1, event, type = "counting") ~
                pten4_cytoplasm + figo + tumourafter +
                ageatdiagnosis + I(ageatdiagnosis^2) + strata(site) +
                mutation_type + logtreatmentresponse + cancer.mother))
res = summary(pool(fit),type="all")
data.frame(Estimate=exp(res[,"estimate"]),
          Low =exp(res[,"estimate"]-qnorm(.975)*res[,"std.error"]),
          High=exp(res[,"estimate"]+qnorm(.975)*res[,"std.error"]),
          T = res[,"t"],pval=pval.fun(res[,"p.value"]),
          sig=sig.fun(res[,"p.value"]),lambda=res[,"fmi"],
          rownames=rownames(res))
```

```
###
### comparison to complete-case analysis
###
levelw = levels(otta[,id.mainpred$id[pw]])[-1]
n.levelw = length(levelw)
colw       = colnames(coxph_model_cancer_mainpred_def[[dw]][[pw]][[cw]][[mw]])
ar.res.lidm = array(NA,dim=c(nlevels(otta$pten4_cytoplasm)-1,
                      length(colw),n.def,n.model),
                 dimnames=list(levels(otta$pten4_cytoplasm)[-1],colw,
                         id.def$id,id.model$id))
for(dww in 1:n.def){
    for(mww in 1:n.model){
        ar.res.lidm[,,dww,mww] = as.matrix(coxph_model_cancer_mainpred_def[[dww]][[pw]][[cw]][[mww]][1:
    }
}
```

```
# PLOT
par(mfrow=c(1,1),omi=c(0,0,.2,0),mar=c(6,4,0,1))
## hazard ratios
plot(1,1,pch="",xlab="",ylab="Hazard ratio",ylim=c(0.5,1.25),xlim=c(.5,n.levelw+.5),axes=FALSE)
abline(h=seq(0,1.2,.1),col="light gray",lty=3)
abline(h=1,col="gray",lwd=1.5)
#
for(lw in 1:n.levelw){
    # complete case
    arrows(lw-.1,ar.res.lidm[lw,1,dw,mw],lw-.1,ar.res.lidm[lw,3,dw,mw],
           code=3,col="orange",angle=90,length=.05)
    points(lw-.1,ar.res.lidm[lw,2,dw,mw],col="orange",pch=1)
    # mice
    lmh = exp(res[lw,1]+res[lw,2]*c(-qnorm(.975),0,qnorm(.975)))
    arrows(lw+.1,lmh[1],lw+.1,lmh[3],
           code=3,col="blue",angle=90,length=.05)
    points(lw+.1,lmh[2],col="blue",pch=1)
    }
# axes
axis(2,las=2)
axis(1,1:n.levelw,levelw,tick=FALSE,las=2,cex.axis=.75)
# legend
legend("top",ncol=1,col=c("orange","blue"),lty=1,box.lwd=NA,
       legend=c("complete-cases","with imputation"))
# title
mtext(id.mainpred$name[pw],side=3,outer=TRUE,cex=1.25)
```

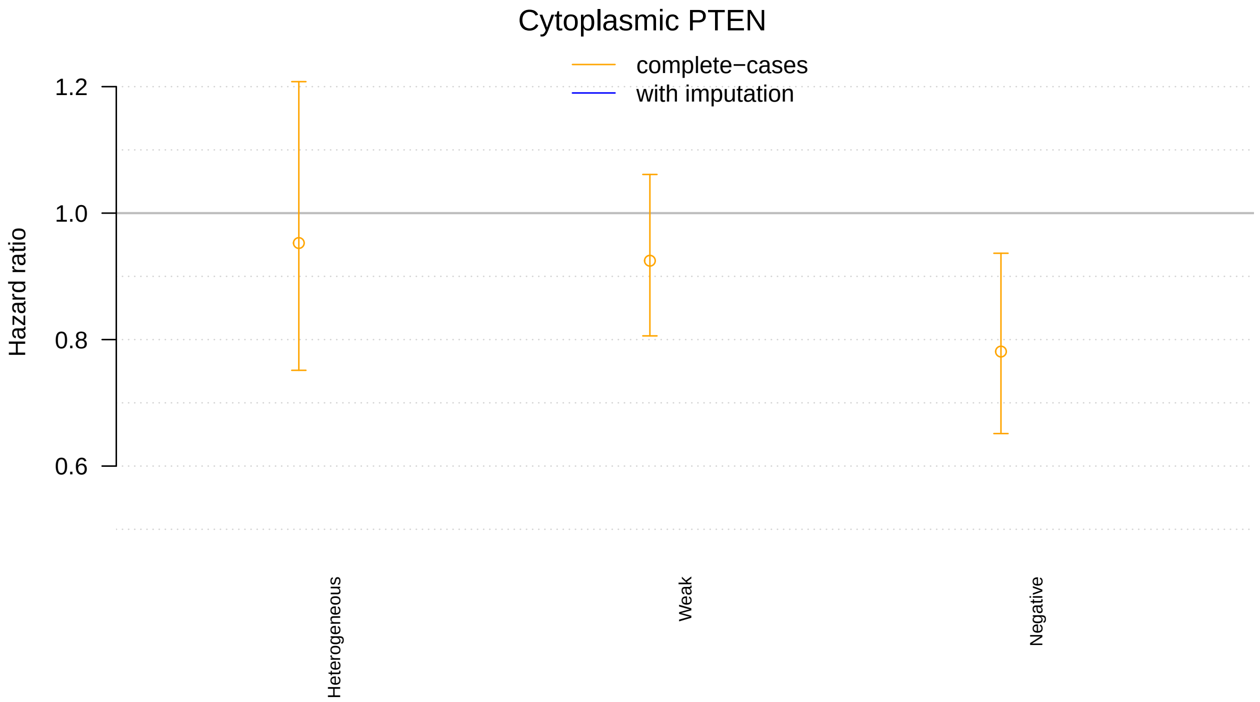

In this case, when using estimations based on multiple imputations, we can note

- an attenuation of the PTEN effects of interest with an effect which remains close to significance at the 5% level (p-value = 0.0517).
- a slight decrease in the variability of the estimator.

- that **mutation type** and **CA125 treatment response** have a significant impact of survival.

The fraction of missing information statistics of the variables *presence of residual disease post-surgery*, and *cancer in the mother* are fairly high.

4.2.2 Conclusions

Whilst still close to significance at the 5% level, the effects of PTEN negative compared to PTEN positive appears to be attenuated when compared to the one of the complete-case analysis.

Study	IRB Committee	Informed Consent
VAN	University of British Columbia - British Columbia Cancer Agency Research Ethics Board	Some cases Yes and some cases No / pathology material
AOV	Alberta Health Services, Research Ethics	No / pathology material
SEA	Cambridgeshire 4 Research Ethics Committee	Yes
MAY1	Institutional Review Board of Mayo Clinic	Yes
NOT	National Health Service National Research Ethics Service Derbyshire Research Ethics Committee	No / pathology material
MAY2	Institutional Review Board of Mayo Clinic	Yes
STA	Stanford University Administrative Panel on Human Subjects in Medical Research	Yes
LAX	Institutional Review Board 3 of Cedars-Sinai Medical Center	Yes
BAV	Ethics Committee of the Friedrich-Alexander-University Erlangen-Nuremberg	Yes
TUE	Ethics-Committee at the Medical Faculty and at the University Hospital of Tübingen	Yes
TVA	University of Calgary, Conjoint Health Research Ethics Committee	Yes
POC	Bioethical Committee of Pomeranian Medical University	Yes
HAW	University of Hawaii, Committee on Human Studies	Yes
CNI	Bioethics and Animal Welfare Committee of the Carlos III Health Institute	Yes
BRZ	Research Ethics Committee of Hospital das Clínicas of the Ribeirão Preto Medical School	No / pathology material
UKO	National Health Service Central Office for Research Ethics Committees (COREC) and The Joint University College London/University College London Hospital Committee on the Ethics of Human Research (Committee A)	Yes
CAL	University of Calgary, Faculty of Medicine, Office of Medical Bioethics	No / pathology material
AOC	Peter MacCallum Cancer Centre Human Research Ethics Committee	Yes
GER	Ethics Committee of the Heidelberg University Clinic	Yes
MAL	Scientific Ethics Committees for Copenhagen and Frederiksberg municipalities and the Danish Data Protection Agency	Yes
HOP	University of Pittsburgh Insititutional Review Board and Roswell Park Cancer Institute Insititutional Review Board	Yes

Institutional ethics approvals
