# Supplementary material for: Clinical and pathological associations of PTEN expression in ovarian cancer: a multicentre study from the Ovarian Tumour Tissue Analysis Consortium
Source: Br J Cancer. 2020 Jun 18;123(5):793–802. doi: 10.1038/s41416-020-0900-0 (PMC7463007; doi:10.1038/s41416-020-0900-0)
Supplement: Supplementary file 2 — Supplementary Figures and Tables [file 41416_2020_900_MOESM2_ESM.docx]

*Supplementary Figure 1* **Missing data pattern in the OTTA dataset.** Missing data per variable ordered by missingness level (y-axis) for participants ordered by site (x-axis). Available data appear in grey. Missing data are color coded according to the participant site, highlighting that missing data is site-dependent, with some sites having few or no AR, PR, ER or FIGO measures.

**

*Supplementary Figure 2* **Comparison of the cytoplasmic and nuclei PTEN scoring of 2 raters**

*Supplementary Figure 3* **Prevalence of different levels of PTEN expression across histotypes of ovarian cancer.** (A) Frequency of different scoring for PTEN expression using IHC per histotype, (B) Proportion of PTEN scoring per histotype.

|  |  | **Number of person-years (% per cancer subtype)** | | | | |  |
| --- | --- | --- | --- | --- | --- | --- | --- |
|  |  | **Cancer type** | | | | |  |
|  |  | **HGSOC** | **ENOC** | **CCOC** | **MOC** | **LGSOC** | **Total** |
|  | **Total** | 14252 (100) | 6227 (100) | 4871 (100) | 2616 (100) | 1264 (100) | 29230 (100) |
| **Status at follow up** | **Data available** | 14052 (100) | 6140 (100) | 4780 (100) | 2597 (100) | 1242 (100) | 28811 (100) |
|  | **Alive** | 6538 ( 47) | 4874 ( 79) | 3670 ( 77) | 2157 ( 83) | 808 ( 65) | 18047 ( 63) |
|  | **Dead (disease)** | 5326 ( 38) | 475 ( 8) | 454 ( 9) | 126 ( 5) | 300 ( 24) | 6681 ( 23) |
|  | **Dead (treatment)** | 11 ( 0) | 23 ( 0) | 15 ( 0) | 0 ( 0) | 1 ( 0) | 50 ( 0) |
|  | **Dead (other)** | 586 ( 4) | 322 ( 5) | 225 ( 5) | 126 ( 5) | 16 ( 1) | 1275 ( 4) |
|  | **Dead (unknown)** | 1591 ( 11) | 446 ( 7) | 416 ( 9) | 188 ( 7) | 117 ( 9) | 2758 ( 10) |
| **FIGO stage** | **Data available** | 12047 (100) | 4711 (100) | 3862 (100) | 1967 (100) | 985 (100) | 23572 (100) |
|  | **Stage I** | 1501 ( 12) | 2678 ( 57) | 2275 ( 59) | 1623 ( 83) | 299 ( 30) | 8376 ( 36) |
|  | **Stage II** | 1680 ( 14) | 1375 ( 29) | 1165 ( 30) | 166 ( 8) | 126 ( 13) | 4512 ( 19) |
|  | **Stage III** | 7723 ( 64) | 627 ( 13) | 396 ( 10) | 167 ( 8) | 520 ( 53) | 9433 ( 40) |
|  | **Stage IV** | 1143 ( 9) | 31 ( 1) | 26 ( 1) | 11 ( 1) | 40 ( 4) | 1251 ( 5) |
| **Differentiation** | **Data available** | 13128 (100) | 5956 (100) | 3120 (100) | 2468 (100) | 1234 (100) | 25906 (100) |
|  | **Well** | 0 ( 0) | 2652 ( 45) | 155 ( 5) | 1125 ( 46) | 1085 ( 88) | 5017 ( 19) |
|  | **Moderate** | 1588 ( 12) | 2021 ( 34) | 538 ( 17) | 1090 ( 44) | 8 ( 1) | 5245 ( 20) |
|  | **Poor/None** | 11540 ( 88) | 1283 ( 22) | 2427 ( 78) | 253 ( 10) | 141 ( 11) | 15644 ( 60) |
| **Residual tumour** | **Data available** | 9025 (100) | 3336 (100) | 2902 (100) | 1403 (100) | 682 (100) | 17348 (100) |
|  | **yes** | 3986 ( 44) | 301 ( 9) | 253 ( 9) | 143 ( 10) | 235 ( 34) | 4918 ( 28) |
|  | **no** | 5039 ( 56) | 3035 ( 91) | 2649 ( 91) | 1260 ( 90) | 447 ( 66) | 12430 ( 72) |
| **Cytoplasmic PTEN** | **Data available** | 12857 (100) | 5746 (100) | 4500 (100) | 2271 (100) | 1060 (100) | 26434 (100) |
|  | **negative** | 2770 ( 22) | 2174 ( 38) | 1462 ( 32) | 452 ( 20) | 131 ( 12) | 6989 ( 26) |
|  | **weak** | 6314 ( 49) | 2240 ( 39) | 2248 ( 50) | 881 ( 39) | 505 ( 48) | 12188 ( 46) |
|  | **positive** | 3043 ( 24) | 1131 ( 20) | 720 ( 16) | 808 ( 36) | 354 ( 33) | 6056 ( 23) |
|  | **heterogeneous** | 730 ( 6) | 201 ( 3) | 70 ( 2) | 130 ( 6) | 70 ( 7) | 1201 ( 5) |
| **Nuclear PTEN** | **Data available** | 12817 (100) | 5740 (100) | 4485 (100) | 2271 (100) | 1060 (100) | 26373 (100) |
|  | **0%** | 5475 ( 43) | 3749 ( 65) | 2168 ( 48) | 1246 ( 55) | 438 ( 41) | 13076 ( 50) |
|  | **]0,10]%** | 3227 ( 25) | 976 ( 17) | 1013 ( 23) | 422 ( 19) | 299 ( 28) | 5937 ( 23) |
|  | **]10,50]%** | 3101 ( 24) | 789 ( 14) | 875 ( 20) | 370 ( 16) | 272 ( 26) | 5407 ( 21) |
|  | **]50,100]%** | 1014 ( 8) | 226 ( 4) | 429 ( 10) | 233 ( 10) | 51 ( 5) | 1953 ( 7) |
| **CD8 count** | **Data available** | 12734 (100) | 5613 (100) | 4396 (100) | 2087 (100) | 842 (100) | 25672 (100) |
|  | **0 TIL** | 1791 ( 14) | 1428 ( 25) | 2120 ( 48) | 939 ( 45) | 220 ( 26) | 6498 ( 25) |
|  | **1-2 TIL** | 2006 ( 16) | 854 ( 15) | 825 ( 19) | 467 ( 22) | 229 ( 27) | 4381 ( 17) |
|  | **3-19 TIL** | 5619 ( 44) | 2392 ( 43) | 935 ( 21) | 595 ( 29) | 344 ( 41) | 9885 ( 39) |
|  | **20+ TIL** | 3318 ( 26) | 939 ( 17) | 516 ( 12) | 86 ( 4) | 49 ( 6) | 4908 ( 19) |
| **AR expression** | **Data available** | 10808 (100) | 4608 (100) | 3664 (100) | 1886 (100) | 900 (100) | 21866 (100) |
|  | **Negative** | 6772 ( 63) | 3111 ( 68) | 3454 ( 94) | 1825 ( 97) | 525 ( 58) | 15687 ( 72) |
|  | **Positive** | 4036 ( 37) | 1497 ( 32) | 210 ( 6) | 61 ( 3) | 375 ( 42) | 6179 ( 28) |
| **PR expression** | **Data available** | 7897 (100) | 4743 (100) | 3910 (100) | 1810 (100) | 535 (100) | 18895 (100) |
|  | **Negative** | 4904 ( 62) | 1169 ( 25) | 3645 ( 93) | 1563 ( 86) | 207 ( 39) | 11488 ( 61) |
|  | **1-50 pos** | 2213 ( 28) | 809 ( 17) | 168 ( 4) | 152 ( 8) | 145 ( 27) | 3487 ( 18) |
|  | **50+ pos** | 780 ( 10) | 2765 ( 58) | 97 ( 2) | 95 ( 5) | 183 ( 34) | 3920 ( 21) |
| **ER expression** | **Data available** | 6425 (100) | 3155 (100) | 2852 (100) | 1209 (100) | 446 (100) | 14087 (100) |
|  | **Negative** | 1519 ( 24) | 714 ( 23) | 2494 ( 87) | 986 ( 82) | 79 ( 18) | 5792 ( 41) |
|  | **1-50 pos** | 1524 ( 24) | 561 ( 18) | 126 ( 4) | 61 ( 5) | 72 ( 16) | 2344 ( 17) |
|  | **50+ pos** | 3382 ( 53) | 1880 ( 60) | 232 ( 8) | 162 ( 13) | 295 ( 66) | 5951 ( 42) |

*Table S1* Summary of the demographics of the study cohort stratified by cancer histotype.

|  | **Cytoplasmic PTEN** | | **Nuclei PTEN** | |
| --- | --- | --- | --- | --- |
|  | **Estimate** | **95% CI** | **Estimate** | **95% CI** |
| **Global** | 0.386 | [0.332;0.439] | 0.301 | [0.256;0.347] |
| **Ordinal (weighted kappa)** | 0.587 | [0.534;0.636] | 0.486 | [0.430;0.541] |
| **Heterogenous vs homogenous** | 0.157 | [0.024;0.305] |  |  |
| **Assessable versus non-assessable** | 0.665 | [0.561;0.763] | 0.665 | [0.561;0.763] |

*Table S2* Cohen’s κ agreement coefficients for the cytoplasmic and nucleus measures when considering PTEN as nominal, ordinal, or when focusing on a given characteristic (Heterogenous, Assessable).

| **Variable** | **Test** | **HGSOC** | **ENOC** | **CCOC** | **MOC** | **LGSOC** |
| --- | --- | --- | --- | --- | --- | --- |
| **Tumour after treatment** | **Nominal/Nominal** | 0.0521 | 1.0000 | 1.0000 | 0.1858 | 0.1983 |
|  | **Nominal/Ordinal** | 0.0536 | 1.0000 | 1.0000 | 0.2058 | 0.1658 |
|  | **Ordinal/Ordinal** | 1.0000 | 0.9175 | 0.8368 | 0.0978 | 0.1924 |
| **Differentiation level** | **Nominal/Nominal** | 0.0933 | 0.7317 | 1.0000 | 0.7987 | 1.0000 |
|  | **Nominal/Ordinal** | 0.1400 | 0.5854 | 1.0000 | 0.8827 | 1.0000 |
|  | **Ordinal/Ordinal** | 0.1051 | 0.6615 | 1.0000 | 1.0000 | 1.0000 |
| **FIGO staging** | **Nominal/Nominal** | 0.0521 | 1.0000 | 0.6228 | 0.7987 | 1.0000 |
|  | **Nominal/Ordinal** | 0.2522 | 1.0000 | 0.3459 | 0.8827 | 1.0000 |
|  | **Ordinal/Ordinal** | 1.0000 | 0.9175 | 0.1897 | 0.5556 | 1.0000 |
| **Age group at diagnosis** | **Nominal/Nominal** | 0.0933 | 0.0115 | 1.0000 | 0.7987 | 1.0000 |
|  | **Nominal/Ordinal** | 0.2522 | 0.0001 | 1.0000 | 1.0000 | 1.0000 |
|  | **Ordinal/Ordinal** | 0.7598 | <0.0001 | 1.0000 | 1.0000 | 1.0000 |
| **CD8** | **Nominal/Nominal** | 0.0521 | 0.6474 | 0.0098 | 0.7987 | 1.0000 |
|  | **Nominal/Ordinal** | 0.0052 | 0.1797 | 0.0001 | 1.0000 | 0.3630 |
|  | **Ordinal/Ordinal** | 0.3074 | 0.0581 | <0.0001 | 1.0000 | 1.0000 |
| **AR** | **Nominal/Nominal** | 0.0008 | 0.0587 | 1.0000 | 0.4956 | 1.0000 |
|  | **Nominal/Ordinal** | 0.0007 | 0.0587 | 1.0000 | 0.5782 | 1.0000 |
|  | **Ordinal/Ordinal** | 0.0008 | 0.0050 | 0.9603 | 0.5556 | 0.4657 |
| **PR** | **Nominal/Nominal** | 0.0114 | 1.0000 | 1.0000 | 0.1858 | 0.7482 |
|  | **Nominal/Ordinal** | 0.0330 | 1.0000 | 1.0000 | 0.8827 | 0.1605 |
|  | **Ordinal/Ordinal** | 0.0621 | 0.9175 | 0.8368 | 0.5556 | 0.0640 |
| **ER** | **Nominal/Nominal** | 0.0026 | 1.0000 | 1.0000 | 0.7987 | 0.1983 |
|  | **Nominal/Ordinal** | 0.0004 | 0.4250 | 0.4617 | 0.8827 | 0.0264 |
|  | **Ordinal/Ordinal** | 0.0002 | 0.4679 | 0.2290 | 0.5556 | 0.0125 |

*Table S3* For multiplicity adjusted p-values of generalised Cochran-Mantel-Haenszel tests analysing the association between Cytoplasmic PTEN and different (ordinal) factors. Such tests correspond to (i) Pearson's Chi-Square tests when analysing the association between two nominal variables, (ii) extended Cochran-Armitage tests when analysing the association between a nominal and an ordinal variable, (iii) linear-by-linear association tests when analysing the relationship between two ordinal variables. Cytoplasmic PTEN is considered as a nominal variable in the ‘nominal/nominal’ and ‘nominal/ordinal’ analyses and is considered as ordinal when discarding the category 'heterogenous' in the ‘ordinal/ordinal’ analyses. The used Holm multiplicity correction allows to achieve a 5% global type I error at the cancer-type and analysis (‘nominal/nominal’, ‘nominal/ordinal’ and ‘ordinal/ordinal’) level.

|  |  | **Hazard ratio** | | | **Inference** | |
| --- | --- | --- | --- | --- | --- | --- |
| **Histotype** | **PTEN Level** | **Low** | **Mid** | **High** | ***p*-value** | **Sig.** |
| **HGSOC** | **Heterogeneous** | 0.751 | 0.953 | 1.208 | 0.9628 |  |
|  | **Weak** | 0.806 | 0.925 | 1.061 | 0.5629 |  |
|  | **Negative** | 0.651 | 0.781 | 0.937 | 0.0219 | * |
| **ENOC** | **Heterogeneous** | 1.132 | 3.239 | 9.262 | 0.0733 | . |
|  | **Weak** | 0.636 | 1.176 | 2.174 | 0.9147 |  |
|  | **Negative** | 0.843 | 1.582 | 2.968 | 0.3411 |  |
| **CCOC** | **Heterogeneous** | 0.5 | 1.473 | 4.341 | 0.8183 |  |
|  | **Weak** | 0.544 | 0.867 | 1.382 | 0.8754 |  |
|  | **Negative** | 0.567 | 0.929 | 1.523 | 0.9822 |  |
| **MOC** | **Heterogeneous** | 0.521 | 1.604 | 4.942 | 0.7636 |  |
|  | **Weak** | 0.684 | 1.278 | 2.387 | 0.7965 |  |
|  | **Negative** | 0.195 | 0.472 | 1.145 | 0.2406 |  |
| **LGSOC** | **Heterogeneous** | 0.317 | 0.82 | 2.121 | 0.9635 |  |
|  | **Weak** | 0.529 | 0.863 | 1.409 | 0.9024 |  |
|  | **Negative** | 0.75 | 1.446 | 2.786 | 0.5886 |  |

*Table S4* Hazard ratio estimates, 95% CI and for-multiplicity-corrected p-values of Cox proportional hazard models fitted on the complete cases of the OTTA dataset per histotype. The outcome of interest is the disease-specific survival within 10 years of diagnosis with ovarian cancer. Survival times were considered as left-truncated due to delayed entries, as well as right-censored for patients still alive at time of last follow-up or dead due to other causes. For all histotypes except LGSOC, we controlled for the variables age, stage, grade and presence of residual disease post-surgery. Analyses were stratified by site. The used multiplicity correction takes the dependence between the PTEN parameters of interest into account and allows to get a global 5% type I error per histotype.

| **Study** | **IRB Committee** | **Informed Consent** |
| --- | --- | --- |
| VAN | University of British Columbia - British Columbia Cancer Agency Research Ethics Board | Some cases Yes and some cases No / pathology material |
| AOV | Alberta Health Services, Research Ethics | No / pathology material |
| SEA | Cambridgeshire 4 Research Ethics Committee | Yes |
| MAY1 | Institutional Review Board of Mayo Clinic | Yes |
| NOT | National Health Service National Research Ethics Service Derbyshire Research Ethics Committee | No / pathology material |
| MAY2 | Institutional Review Board of Mayo Clinic | Yes |
| STA | Stanford University Administrative Panel on Human Subjects in Medical Research | Yes |
| LAX | Institutional Review Board 3 of Cedars-Sinai Medical Center | Yes |
| BAV | Ethics Committee of the Friedrich-Alexander-University Erlangen-Nuremberg | Yes |
| TUE | Ethics-Committee at the Medical Faculty and at the University Hospital of Tübingen | Yes |
| TVA | University of Calgary, Conjoint Health Research Ethics Committee | Yes |
| POC | Bioethical Committee of Pomeranian Medical University | Yes |
| HAW | University of Hawaii, Committee on Human Studies | Yes |
| CNI | Bioethics and Animal Welfare Committee of the Carlos III Health Institute | Yes |
| BRZ | Research Ethics Committee of Hospital das Clínicas of the Ribeirão Preto Medical School | No / pathology material |
| UKO | National Health Service Central Office for Research Ethics Committees (COREC) and The Joint University College London/University College London Hospital Committee on the Ethics of Human Research (Committee A) | Yes |
| CAL | University of Calgary, Faculty of Medicine, Office of Medical Bioethics | No / pathology material |
| AOC | Peter MacCallum Cancer Centre Human Research Ethics Committee | Yes |
| GER | Ethics Committee of the Heidelberg University Clinic | Yes |
| MAL | Scientific Ethics Committees for Copenhagen and Frederiksberg municipalities and the Danish Data Protection Agency | Yes |
| HOP | University of Pittsburgh Insititutional Review Board and Roswell Park Cancer Institute Insititutional Review Board | Yes |

*Institutional ethics approvals*
